# Evaluating hearing performance with cochlear implants within the same patient using daily randomization and imaging-based fitting - The ELEPHANT study

**DOI:** 10.1186/s13063-020-04469-x

**Published:** 2020-06-23

**Authors:** L. J. G. Lambriks, M. van Hoof, J. A. Debruyne, M. Janssen, J. Chalupper, K. A. van der Heijden, J. R. Hof, C. A. Hellingman, E. L. J. George, E. M. J. Devocht

**Affiliations:** 1 Department of ENT/Audiology, School for Mental Health and Neuroscience (MHeNs), Maastricht University Medical Center, Maastricht, The Netherlands; 2 Department of Methodology and Statistics, School for Public Health and Primary Care (CAPHRI), Maastricht University Medical Center, Maastricht, The Netherlands; 3 Advanced Bionics European Research Centre (AB ERC), Hannover, Germany

**Keywords:** Cochlear implant, Daily randomization, Imaging based fitting, Tonotopy, Bimodal hearing

## Abstract

**Background:**

Prospective research in the field of cochlear implants is hampered by methodological issues and small sample sizes. The ELEPHANT study presents an alternative clinical trial design with a daily randomized approach evaluating individualized tonotopical fitting of a cochlear implant (CI).

**Methods:**

A single-blinded, daily-randomized clinical trial will be implemented to evaluate a new imaging-based CI mapping strategy. A minimum of 20 participants will be included from the start of the rehabilitation process with a 1-year follow-up period. Based on a post-operative cone beam CT scan (CBCT), mapping of electrical input will be aligned to natural place-pitch arrangement in the individual cochlea. The CI’s frequency allocation table will be adjusted to match the electrical stimulation of frequencies as closely as possible to corresponding acoustic locations in the cochlea. A randomization scheme will be implemented whereby the participant, blinded to the intervention allocation, crosses over between the experimental and standard fitting program on a daily basis, and thus effectively acts as his own control, followed by a period of free choice between both maps to incorporate patient preference. With this new approach the occurrence of a first-order carryover effect and a limited sample size is addressed.

**Discussion:**

The experimental fitting strategy is thought to give rise to a steeper learning curve, result in better performance in challenging listening situations, improve sound quality, better complement residual acoustic hearing in the contralateral ear and be preferred by recipients of a CI. Concurrently, the suitability of the novel trial design will be considered in investigating these hypotheses.

**Trial registration:**

ClinicalTrials.gov: NCT03892941. Registered 27 March 2019.

## Background

Although cochlear implants (CI) are now considered to be the main medical treatment for patients suffering from severe-to-profound sensory-neural hearing loss, their current-day performance relies mainly on technical improvements that were implemented in their early development [1]. CI performance appears to have reached a plateau in the last 30 years and despite an increase in scientific publications [2], a substantial number of challenges await future research. For example, a CI user’s individual gradient of improvement is still hard to predict [3], disappointing outcomes remain hard to explain [4], speech perception in difficult listening situations remains extremely challenging for most CI users [5], and the quality of sound generated by CI stimulation is often considered unnatural and robotic despite decades of CI development [4, 6].

### Current limitations in CI research

CI research is hampered by practical limitations such as the high costs of medical devices [7] and limited availability of potential research participants [8]. This inhibits innovation and experimental procedures. Moreover, due to high inter-individual variability in post-operative hearing performance [9], CI studies require large numbers of participants to obtain adequate statistical power. However, acquiring large numbers of participants is often not feasible due to competition between CI manufacturers for CI candidates. That is, cross-brand comparisons introduce additional variability in a study.

As a consequence of these difficulties in participant recruitment in CI studies, randomized controlled trials (RCTs) measuring CI hearing performance are rare. RCTs are considered to provide the highest level of evidence in terms of experimental design [10], yet require large sample sizes due to their between-subject design. In a traditional RCT, subjects are randomly allocated to one of two groups: one (the treatment group) receives the experimental intervention, and the other (the control group) is treated according to clinical routine. Between-group comparisons are made to test for differences in outcome [11]. Such designs are relatively scarce for measuring CI performance-related outcomes [12–15]. The few RCTs that have succeeded in including more than 30 participants recruited those subjects at more than one study center and used devices from multiple manufacturers, adding to study variability [16, 17]. RCTs that are limited to a monocentric study setup often contain much smaller sample sizes [18, 19].

In contrast, prospective studies with a crossover design (which require fewer participants) are the more commonly preferred alternative [20–22]. In this trial type, study subjects are allocated to a first treatment arm and then, possibly after a wash-out period, re-allocated to a second intervention phase. However, an eminent problem when implementing this design in order to evaluate CI rehabilitation performance is the occurrence of a first-order carryover effect [23] due to neural reorganization in the subcortical and cortical auditory system following CI implantation [24–26]. Specifically, neuroimaging studies demonstrate extensive neural plasticity in the auditory pathway with changes in sensory input [27, 28]. For example, it has been shown that after prolonged periods of deafness, other sensory modalities are able to activate auditory regions [29, 30]. Moreover, in patients with CI, it is evident that brain reorganization plays a crucial role in achieving benefit from the CI: after implantation there is an adaption period during which the auditory system learns to efficiently extract information from the CI stimulation [26]. Due to this learning effect and the underlying brain reorganization, there may be a bias towards an experimental intervention that is given first during the initial rehabilitation period. For example, it is conceivable that CI users receiving intervention A followed by intervention B will generally favor intervention A as a result of initial post-implantation brain plasticity rather than as a result of the beneficial properties of intervention A. This bias restricts the use of a conventional prospective crossover trial setup for CI research and effectively requires a parallel test-versus-control setup. As aforementioned, however, such an RCT design doubles the number of participants required to achieve reasonable statistical power, and also increases the risks of suboptimal treatment and outcomes in one of the two groups.

### Development of an alternative trial design

To counteract current limitations in CI research, we believe an alternative methodology that allows for experimental interventions in a prospective trial setup is necessary. We therefore propose a study design for a clinical trial that is feasible, takes a within-subject perspective, and in which the participants are their own control. This design is similar to the traditional crossover perspective, but uses daily crossover randomization as a method for simultaneous utilization of study conditions instead of a crossover in subsequent, consecutive periods. By using daily crossover randomization, participants may switch between control and intervention on a daily basis. This is followed by a period of free choice between both conditions to incorporate participant preference. It is proposed that this new approach will facilitate data collection from small sample sizes, reduce the impact of individual subject characteristic variability, decrease the number of subjects needed to find a moderate statistical effect, address initial brain plasticity, and prevent the occurrence of a first-order carryover effect.

### The ELEPHANT study

In the “Electrically place-pitched hearing achieves natural tonotopy” (ELEPHANT) study, the alternative trial design will be incorporated in a clinical trial focusing on tonotopical frequency allocation in people with CI, based on imaging. In current clinical practice, the frequencies assigned to CI electrodes follow a one-size-fits-all approach, assuming an average cochlear tonotopy. However, electrode locations within the cochlea are different in each patient. As a result, these fixed frequency allocation tables (FAT) cause a mismatch between electrical frequency information and acoustical tonotopical placement [31]. The experimental intervention in this study will involve mapping of electrical input aligned to the individual cochlea, based on post-operative cone beam computed tomography (CBCT). The frequency mapping of the CI will be adjusted to match the frequency distribution across the electrode array as closely as possible to the corresponding acoustic locations. Each study participant receives two CI processors, one of which will be programmed with the experimental FAT and the other with the standard FAT as in standard clinical practice. Participants will then switch between both processors according to a daily-randomized wearing schedule.

By using the method of daily crossover randomization we set out to measure the relative learning performance of the two fitting maps while preventing unwanted bias as a result of brain plasticity related to the map that was presented first. The resulting “learning curve” in hearing performance is a primary outcome measure that has received relatively little attention in the literature on CIs [32, 33]. Factors such as age, cognition, prior speech performance, pathology, and duration of hearing loss are all likely to influence the learning curve [34, 35]. In a daily-randomized setup, however, these factors can be expected to affect both the test and control intervention equally, thereby diminishing their effect at both the individual and group level.

Those participants that retain the use of a contralateral hearing aid (HA) will also receive an experimental bimodal fitting. Gain adjustments will be based on loudness scaling measurements, thereby aiming to match natural loudness perception as closely as possible.

### Current hypotheses

Here, it is hypothesized that hearing outcomes with a CI will improve when electrical stimulation is matched as closely as possible to the natural tonotopy of a normal hearing human brain, instead of relying on plasticity to adapt to an induced mismatch. The imaging-based, individual fitting strategy is thought to give rise to a steeper learning curve, and result in better performance in challenging listening situations, improve sound quality, and better complement residual acoustic hearing in the contralateral ear. The experimental hearing aid fit is expected to restore natural loudness perception as much as possible and to be preferred by research subjects. The suitability of the novel trial design will be considered concurrently with the investigation of these hypotheses.

## Methods/design

### Design, setting and recruitment

This study is a single blinded, controlled clinical trial with daily crossover randomization, which will be implemented directly from the start of the CI rehabilitation process. Its protocol has been approved by the ethics committee of the Maastricht University Medical Center (MUMC+)(NL64874.068.18 / METC 18–028) and has been registered at clinicaltrials.gov (NCT03892941).

**Fig. 1:**
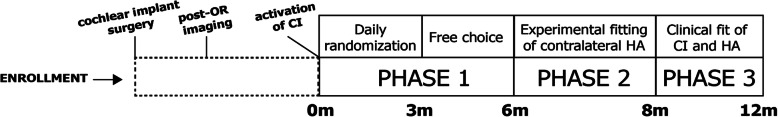
Study outline. During phase 1, participants combine their CI rehabilitation with exposure to both an experimental and a standard fitting program. This distribution is based on a daily randomized scheme in the first 3 months, after which an equal period of free choice is incorporated. Patients who continue to use a contralateral hearing aid will receive experimental hearing aid fitting in phase 2. During phase 3, a clinical fit will be performed for both CI and hearing aid based on indicated preferences obtained during the study period for either experimental or standard settings

A minimum of 20 and a maximum of 30 participants will be included, with a 1-year follow-up period after first fitting. The study outline can be classified in 3 different phases (Fig. 1). During phase 1, starting at first fit, CI optimization will take place during which subjects follow a randomization scheme whereby each individual crosses over between the experimental and standard fitting program. A period of free choice follows in which patients have the liberty of choosing whatever program they prefer, thereby incorporating patient preference as an outcome in the trial design. In phase 2, starting at 6 months after first fit, those participants who choose to retain a hearing aid in the contralateral ear will receive an experimental fitting of their HA based on loudness scaling. Participants that do not wear a contralateral hearing aid, or who have worn their hearing aid less than half of their CI wearing time, will receive no intervention during phase 2. During phase 3, starting at 8 months after first fit, participants will receive the final fit of their CI and their hearing aid based on the preferences they have obtained during phase 1 and phase 2.

Recruitment started March 2019 and takes place in the Maastricht UMC+, a tertiary university medical center. Subjects from the CI selection cohort of the CI-team South-East Netherlands are screened and included by informed consent at the latest 1 week before surgical placement of the CI. Eligible participants are as follows: (1) aged 18 years or older, (2) known to have post-lingual onset of profound deafness (> 4 years of age), (3) meet the Dutch criteria for CI implantation and (4) selected to receive a HIRes Ultra implant with HiFocus Midscala electrode array (Advanced Bionics, Sylmar, CA, USA). Exclusion criteria are (1) contraindications for magnetic resonance (MR) or computed tomography (CT) imaging, (2) cochlear or neural abnormalities that could compromise the placement of the electrode or affect outcome measures, (3) implementation of electric-acoustic stimulation (EAS) within the first year of follow up, (4) previous or bilateral implantation of a CI and (5) additional disabilities that could prevent active trial participation.

All relevant trial resources will be made available through the supplemental materials published with this article. These include the research protocol, the case report form and a complete overview of the study outline.

### Daily-randomization procedure

**Fig. 2:**
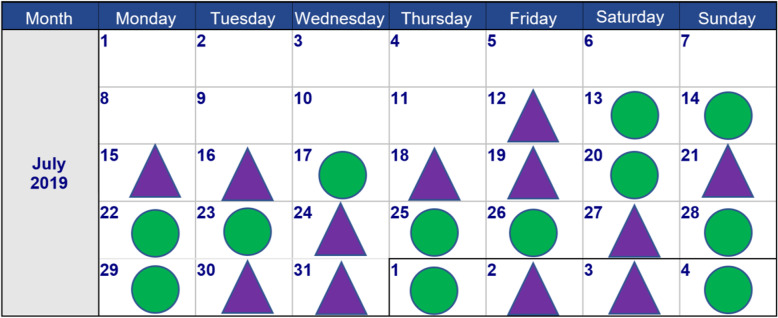
Example of a daily-randomization scheme. Green and purple labels indicate which cochlear implant (CI) processor to wear

In this new type of trial design subjects act as their own control and treatment allocation is based on daily randomization. Two speech processors (physically labeled with either a green circle or a purple triangle) will be given to each participant, one of which will be programmed with the experimental settings and the other with the standard settings. Each day, subjects will be allocated to wear one of the two processors for a period of 3 months. A scheme with corresponding labels will be provided for each participant (Fig. 2). By equipping participants with two different processors, instead of one processor with FAT settings on different program slots, it is thought that issues with compliance are less likely to occur.

To prevent subjects from developing a preference for one of both programs during the first crucial period of adaptation to the cochlear implant, several prerequisites have been included in the randomization procedure. First, the same processor will not be allocated for more than 2 days consecutively within the first 4 weeks of CI rehabilitation. This restriction will be broadened from 4 weeks on to a maximum of 4 days consecutively. Second, to aid blinding of the participant to the intervention and in order to prevent subjects habitually preferring one processor to the other, the assignment of the two fittings across the two processors is randomized by the fitting clinician. As a result, there is a 50% chance that the experimental program will be saved on either the green or purple processor at each fitting. Participants will be blinded in that they are unaware which CI processor contains the experimental setting at any point in time. During the fitting procedures, participants will not be given any visual or verbal cues that may lead to recognizing which processor contains which settings.

### Study interventions

#### Imaging-based tonotopic distribution

**Fig. 3:**
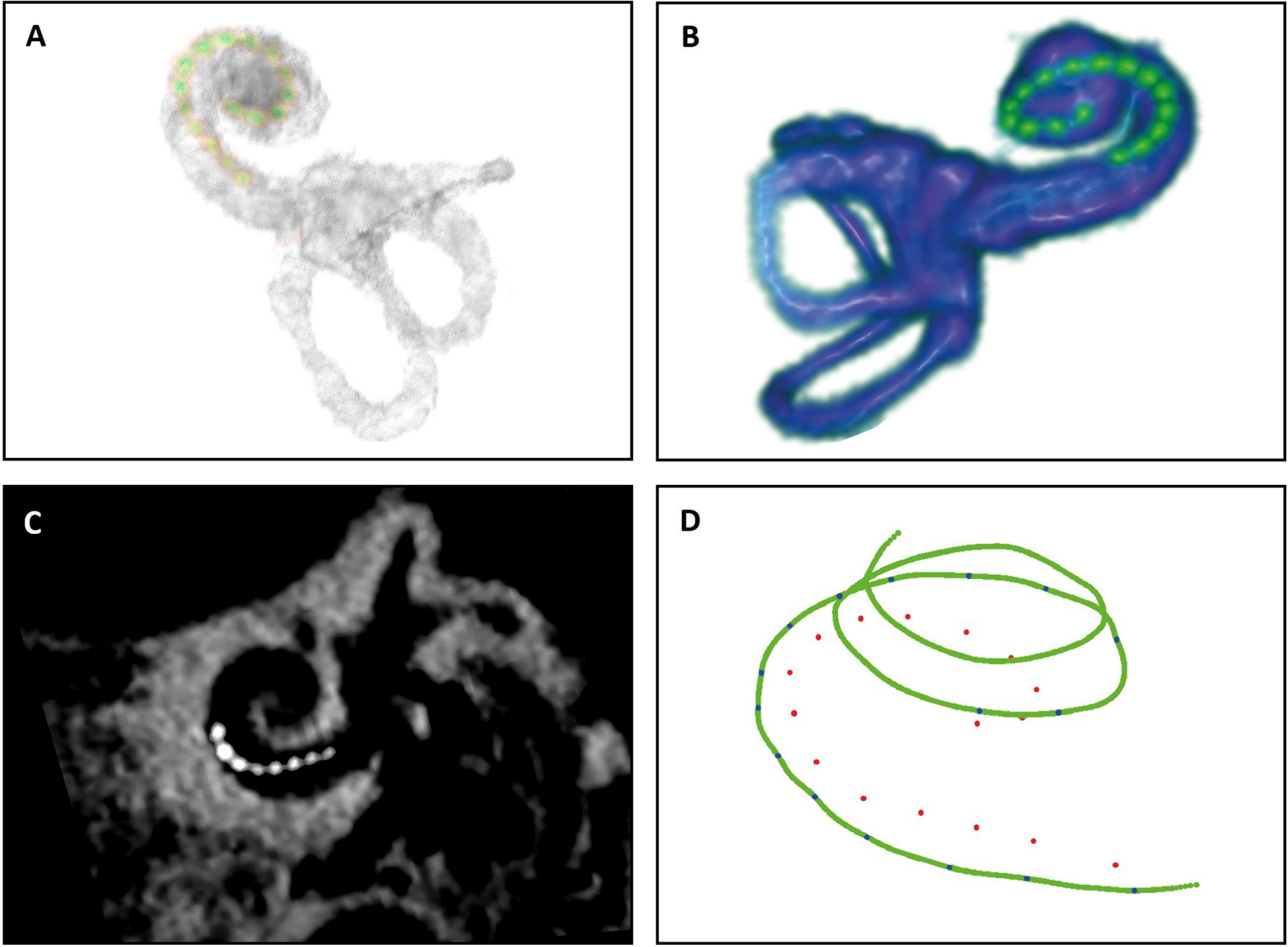
An example of imaging-based tonotopic measurements. **a**, **b** A 3D reconstruction of the cochlear labyrinth with inserted electrodes using a method of image fusion using computed tomography (CT) and cone-beam CT (CBCT) (**a**) and magnetic resonance imaging (MRI) and CBCT (**b**). Images were segmented and rendered with 3D Slicer. **c** Overview of cochlear basal turn in one CBCT slice. **d** Visualization of measurements of lateral wall (green line) and electrode contact positions (red dots) with blue dots representing the closest points on the lateral wall for each electrode

A clinical CT and magnetic resonance imaging (MRI) (if clinically indicated) scan will be acquired prior to CI implantation, as part of the clinical routine, and participants will undergo CBCT 1 week after surgical placement of the CI. The images will then be fused using 3D Slicer [36] and BRAINSFit software [37]. As validated in a previous routine, this procedure generates the high-quality imaging needed for detailed intra-cochlear electrode assessment [38, 39]. Important markers, such as the extent of the lateral and medial wall and the positions of the round window and implant electrodes, are identified. A 3D image of the individual cochlear duct and electrode positions can then be visualized (Fig. 3) and exported to Mathematica software 11.3 (Wolfram Research, Champaign, USA). Identification of the cochlear lateral wall, in contrast to the medial wall or the organ of Corti, is most feasible in this imaging setup. Therefore, the closest point on the lateral wall for each electrode will be calculated. The Advanced Bionics Midscala electrode array used in this study population consists of 16 electrode contacts, and the resulting 16 coordinates that are determined resemble the tonotopical projections of each CI electrode to the neighboring lateral wall. Using the Greenwood function [40], the tonotopical place-pitch alignment of the electrodes in each individual subject can be estimated for the lateral wall.

#### CI frequency mapping

The calculated frequency distribution is used to create the experimental frequency allocation table (FAT), which pursues individualized tonotopical alignment. In this setting, the Advanced Bionics Phantom functionality is enabled [41], which delivers low-frequency information beyond the most apical electrode of the array by operating as a virtual channel created using partial bipolar stimulation [41–43]. By adding a low-frequency channel, the Phantom feature reduces the gap between the actual and tonotopical place of low-frequency stimulation and at the same time increases the number of channels that can be tonotopically matched for the remainder of the array. For details on Phantom stimulation, see Nogueira et al. [42].

The imaging-based frequency-to-place distribution and the Phantom feature are applied to create the experimental FAT by overlapping the tonotopical frequency distribution with its corresponding electrode channels (Table 1). Ideally, this setting should be constructed as such that each position in the cochlea should merely be innervated by frequency inputs that align with its tonotopy. However, full tonotopical alignment is not always appropriate, as there are electrode contacts that fall out of the 250–8000 Hz frequency range that is covered by a CI processor. Due to shallow insertions or irregular lateral wall lengths, it is also possible that the most apical electrodes reach a tonotopical alignment that falls right within the speech spectrum. Leaving out all acoustic input below this point is expected to be detrimental for overall hearing performance. To address these issues, a set of rules will be applied as follows: (1) at least two channels (including Phantom) have to stimulate 1000 Hz or lower, (2) at least four channels have to stimulate 2000 Hz or lower, (3) at least seven channels have to stimulate 4000 Hz or lower and (4) the most basal channel has to be stimulated by 8598 Hz or lower. Participants will be excluded from study participation when it is not possible to assign a minimum of 8 channels [44] within the CI frequency spectrum while applying the rules stated above. This will be monitored throughout the study, with excluded participants taken out of the study and being transferred to standard clinical follow up.

**Table 1:** Frequency mapping of an Advanced Bionics CI speech processor with the standard clinical FAT (left) and an example of an experimental FAT as set up in this study (right) where x = disabled and - = not applicable

Standard FAT				Experimental FAT			
Electrode pair	Lower bound	Upper bound	Bandwidth	Electrode pair	Lower bound	Upper bound	Bandwidth
–	–	–	–	*Phantom*	238	640	402
*1–2*	238	442	204	*1–2*	640	862	222
*2–3*	442	578	136	*2–3*	862	1055	193
*3–4*	578	646	68	*3–4*	1055	1383	328
*4–5*	646	782	136	*4–5*	1383	1770	387
*5–6*	782	918	136	*5–6*	1770	2124	354
*6–7*	918	1054	136	*6–7*	2124	2544	420
*7–8*	1054	1257	203	*7–8*	2544	3155	611
*8–9*	1257	1529	272	*8–9*	3155	3720	565
*9–10*	1529	1801	272	*9–10*	3720	4412	692
*10–11*	1801	2141	340	*10–11*	4412	5176	764
*11–12*	2141	2549	408	*11–12*	5176	6244	1068
*12–13*	2549	3025	476	*12–13*	6244	7239	995
*13–14*	3025	3568	543	*13–14*	7239	8598	1359
*14–15*	3568	4248	680	*14–15*	x	x	–
*15–16*	4248	8054	3806	*15–16*	x	x	–

#### Hardware and software

All participants receive unilateral implantation with a HiRes Ultra 3D implant and will be provided with two Naída CI Q70 speech processors. Those participants with sufficient residual hearing in the contralateral ear will also receive a Naída Link hearing aid (Phonak, Stäfa, Switzerland) from the start of CI rehabilitation. All equipment will be provided by Sonova Holding AG (Stäfa, Switzerland) as loaned devices. CI processors will be programmed as research devices to provide compatibility with research software BEPS+, which will be used for the fitting of both experimental and standard maps. This software package has been developed specifically by Advanced Bionics for research purposes and is enclosed with an Investigational Medical Device Dossier and additional safety checks. The Naída Link hearing aids will be programmed with the clinical Target software (Phonak, Stäfa, Switzerland). In the last part of the study, participants will exchange the loaned CI and hearing aid for their own devices, which will be programmed with clinical software.

#### Loudness fitting of contralateral hearing aid

Most clinically available hearing aid fitting prescriptions are based on hearing thresholds and focus on those frequencies that are important for speech understanding [45, 46]. However, most participants with bimodal hearing only have aidable residual hearing up to 1 kHz [47]. In order to make use of the residual, low-frequency hearing, it is hypothesized that a gain prescription based on restoring natural loudness perception may be better to capture the functions of the low-frequency acoustic hearing. Thus, this so-called bimodal loudness fitting of the hearing aid may be better in complementing the CI and match the natural loudness perception as close as possible.

At the start of the study, all participants who have the intention to wear a contralateral hearing aid during the course of the study, will be fitted using the standard hearing aid fitting for bimodal Advanced Bionics and Phonak users. It can be expected that the majority of participants who wear a contralateral hearing aid will continue to do so after implantation [47]. Those subjects that wear their hearing aid for at least 50% of their CI wearing time during phase 1 of the study will receive the experimental bimodal loudness fitting at the beginning of phase 2. As a starting point for this procedure, loudness perception will be measured with the standard fitting using adaptive categorical loudness scaling (ACALOS) [48]. Differences in loudness perception between hearing with the standard HA fitting and reference values of normal hearing will then be calculated using Matlab R2018B (MathWorks, MA, USA). Gain adjustments will be presented in order to compensate for these discrepancies thereby aiming at a loudness perception that is as close as possible to normal hearing perception. These gain prescriptions will then be imported in Phonak Target software and fitted to the participants’ Naída Link hearing aid. Sound acceptance will be evaluated and broadband gain adjustments up to 6 dB may be made based on patients’ wearing time and preferences. At the end of phase 2, participants will indicate their preference for either the experimental or standard HA fit, which will then be transferred to their final fit at the beginning of phase 3.

### Outcomes

Extensive outcome testing will be performed over the course of the study period (Table 2). Listening conditions vary per visit and per test, but include unilateral hearing with either standard or experimental CI settings, unilateral hearing with hearing aid using standard HA settings or loudness fitting measurements with CI plus hearing aid and unaided measurements. Order of testing will be determined according to a randomization schedule. This is except for CI testing, for which measurements will always start with the processor labeled with a purple triangle, as its allocated settings are already randomized.

**Table 2:** Schedule of enrollment, interventions and assessments

Timepoint	Description	Weeks after CI activation																			
		#	−4	−3	0	1	2	3	4	5	6	7	8	10	12	16	20	26	30	34	52
**Enrollment**																					
Clinical pre-assessment	CI screening selection	X																			
Informed consent		X																			
Surgery			X																		
CBCT scan				X																	
**Interventions**																					
CI experimental phase					X	X	X	X	X	X	X	X	X	X	X	X	X	X			
HA experimental phase																			X		
Clinical fitting phase																				X	X
**Assessments**																					
**Primary outcomes**																					
Patient preference	10-point VAS				X	X	X	X	X	X	X	X	X	X	X	X	X	X		X	X
Word recognition	CNC	X			X	X	X	X	X	X	X	X	X	X	X	X	X	X	X	X	X
Sentence recognition in quiet	Dutch Matrix Sentence Test				X	X	X	X	X	X	X	X	X	X	X	X	X	X	X	X	X
**Secondary outcomes**																					
Sentence recognition in noise	Dutch Matrix Sentence Test				X	X	X	X	X	X	X	X	X	X	X	X	X	X	X	X	X
Spatial hearing	SSPIN													X				X	X	X	
Listening effort									X						X			X	X	X	X
Loudness scaling	ACALOS								X						X			X	X	X	
Frequency selectivity	SMRT								X						X			X		X	
Questionnaires	SSQ-12, HUI-3, ICECAP-A			X														X		X	X
	Sound quality							X							X			X		X	X

*CBCT* cone-beam computed tomography, *CI* cochlear implant, *HA* hearing aid, *VAS* visual analogue scale, *CNC* consonant-nucleus-consonant, *SSPIN* spatial speech perception in noise, *ACALOS* Adaptive Categorical Loudness Scaling, *SSQ-12* Speech, Spatial and Qualities of Hearing, *HUI-3* Health Utilities Index-3, *ICECAP-A* Icepop Capability Measure for Older People

#### Word and sentence recognition

Word recognition will be evaluated with phoneme scoring over the levels 55, 65 (test-retest) and 75 dB sound pressure level (SPL) on a Dutch monosyllabic consonant-nucleus-consonant (CNC) speech recognition test. The average of test-retest values on 65 dB SPL will be the primary outcome values [49]. In addition, the Dutch Matrix Sentence test, which has been validated for repeated measures in CI recipients [50, 51], will be used through 3 listening conditions: (1) quiet, (2) with background noise and (3) with spatially separated background noise. The tests will be administered using the Oldenburg measurement applications software package developed by Hörtech gGmbH Germany. Speech stimuli will be presented at a fixed level of 65 dB SPL from a speaker at ear level at a distance of 1 m in the front (0°). Participants are asked to reconstruct sentences by selecting perceived words from a closed set using a touch screen. Target sentences are selected from a matrix of 50 words (5 columns, 10 words) with a fixed sentence order (name, verb, numeral, adjective, object) with subjects being forced to answer all columns. Each list consists of 10 sentences. When testing in the noise condition, an adaptive procedure is applied whereby stationary-speech-shaped noise is fixed at a level of 65 dB SPL while speech level is varied according to scoring performance using a logistic function [52]. Both speech and noise are presented by the same speaker. The speech reception threshold (SRT), which is defined as the signal-to-noise ratio (SNR) corresponding to a 50% correct score, will be determined. Scores will be excluded from analysis when fewer than two reversals occur or if the SNR ratio is higher than 15 dB [53]. To determine head shadow and squelch effect, and assess benefits of contralateral hearing aid use in particpants with bimodal devices, noise will also be presented from spatially separated loudspeakers as in Devocht et al. [54] with the exception of using 10 sentence-word lists instead of 20. To address potential learning effects [51] and familiarize participants with each task, two training lists will be administered in the condition to be tested first prior to each of the three matrix tests. The mean value of test and retest will be included as an outcome measure.

#### Listening effort

A listening effort test is applied using the same matrix as for sentence recognition. Participants are asked to rate the amount of effort involved in attempting to understand sentences in the presence of noise at different SNRs. During this procedure, as explained in more detail by Devocht et al. [54], sound is presented from a speaker and subjective rating is monitored on a touch screen using a 13-point scale ranging from no effort to extreme effort. The noise level is fixed at 65 dB SPL, while speech level is varied in order to create different SNRs according to the individual SRT of each participant, as determined in the corresponding listening condition during sentence intelligibility in noise. Overall, six levels were set below and above the participant’s individual SRT (SRT − 6, SRT − 3, SRT, SRT + 3, SRT + 6, SRT + 9).

#### Loudness scaling

ACALOS [48] will be performed to estimate the course of loudness perception between minimal audible level and maximum comfortable level with the CI and hearing aid alone. During this procedure, loudness levels are automatically adjusted to the participant’s individual auditory dynamic range without employing any pre-measurement. Participants will be presented with a one third octave band-noise from a speaker (center frequencies 250, 500, 1000, 2000, 4000 Hz) at different loudness levels (range 0–95 dB SPL with upper level depending on frequency), coming from a loudspeaker at the front (0°). After each stimulus, subjects are asked to rate loudness on an 11-point scale ranging from inaudible to too loud on a touch screen. As mentioned above, loudness scaling will be used to perform experimental HA fitting during phase 2.

#### Frequency selectivity

The spectral-temporally modulated ripple test (SMRT, available at www.ear-lab.org/smrt.html) will be used to measure the capability to spectrally resolve frequency information, which is known to be related to performance in understanding speech [55, 56]. This adaptive forced-choice test is used to measure the participant’s ability to discriminate stimuli that are modulated in the frequency domain [57]. During the test, participants are presented with two intervals: one contains a reference stimulus with 20 ripples per octave, the other contains the target stimulus, which has a varying ripple rate. The target stimulus initially has 0.5 ripples per octave, but is modified using a 1-up/1-down procedure with a step size of 0.2 ripples per octave, until the subject can no longer distinguish between reference and target stimulus [57]. Stimuli will be presented from a speaker and participants will be asked to select which stimulus is different. The test is completed after 10 reversals: thresholds are calculated as the average scores of the last 6 reversals. The average values of test-retest are recorded as outcome measures.

#### Questionnaires

Participants will be asked to rate their satisfaction at every visit in a short questionnaire to determine their overall preference for either standard or experimental CI and hearing aid settings in everyday life. Satisfaction will be rated on a 10-point visual analogue scale on the subtopics of speech understanding, sound recognition and sound quality. Other questionnaires that will be administered include the Speech-Spatial-Qualities of Hearing Scale (SSQ-12) [58, 59], the Health Utility Index Mark 3 (HUI-3) [60], the translated [61] Icepop Capability measure for Adults questionnaire (ICECAP-O) [62] and a sound quality questionnaire by Devocht et al. [54] based on the descriptions of Boretzki [63].

### Compliance

In the first 3 months of the study, participants are required to wear their CI processors according to their personal randomization schedule. They should also wear their contralateral hearing aid for at least 50% of the CI wearing time over the course of the first 6 months, in order for the subject to be included for the experimental hearing aid fitting. Participants are asked to report the wearing time of both their CI processors and their hearing aid in daily diaries. By comparing these values to the ratios in the randomization schedule, differences in compliance will be calculated at regular intervals over each time window and compared to a set of cut-off points (see Additional file 1: research protocol). Since data logging features are not currently available in the CI research fitting software BEPS+, the only check on reliability of patient diary reporting is through the data logging of the hearing aid in Phonak Target. In the case of minor randomization deviations, participants will be treated as protocol deviators and will be removed from the per-protocol analysis population. In the case of severe non-compliance with randomization procedures, participants will be removed from the intention-to-treat analysis population.

### Sample size

Complementary to the new study design for clinical trials presented in this paper, thought has also been given to a different method for a priori calculation of sample size and power. Instead of calculating the estimated number of participants needed for a given research study, one could also work the other way around. After all, many clinical trials depend on the supply of patients within a selective pool and do not have any additional recruiting possibilities. This study, which is no exception to that point, will aim to include a minimum sample of 20 participants during an inclusion period of 18–24 months. A maximum of 30 participants will be included if recruitment is prosperous.

Sample size calculation is usually based on the primary study outcome, which in this study is the difference in CNC word recognition (test-retest at 65 dB SPL) between the experimental and standard CI setting after 6 months of rehabilitation. Using the concept of effect size as interpreted by Cohen’s d [64, 65], the following formula could be used to calculate sample size for a paired *t* test:

$$ \mathrm{N}=2+\left({\left({\mathrm{Z}}_{1-\upalpha /2}+{\mathrm{Z}}_{1-\upbeta}\right)}^2/{\mathrm{d}}^2\right) $$

where N = sample size, *α* = type 1 error, *β* = type 2 error, d = effect size*.*

Given the idea of a restricted sample size, it can be argued that it is more valuable to rearrange this formula as to calculate effect size.

$$ \mathrm{d}=\surd \left(2+\left({\left({\mathrm{Z}}_{1-\upalpha /2}+{\mathrm{Z}}_{1-\upbeta}\right)}^2\right)/\mathrm{N}\right) $$

As a result, different outcomes in effect size can be calculated with a sample size range of 20–30 participants, an alpha range of 0.01–0.08 and a given power of 0.08. When considering a type 1 error of 0.05 and a sample size of 20, an effect size of 0.66 or larger can be detected. If 30 study participants are recruited and the alpha level is kept similar, the resulting detectable effect size is at least 0.53.

### Statistical methods, data reporting and analysis

The outline of this study can be categorized in multiple phases, each concerning different statistical outcomes, listening conditions and endpoints. The statistical analysis plan will be finalized before the database lock. In general, analyses will be carried out for each treatment option (experimental fitting versus standard fitting for both CI and hearing aid), for different outcomes (e.g. word recognition, subject preference, quality of life) and different listening conditions (with CI, hearing aid or bimodal). The main statistical analysis will focus on the question of whether the experimental fitting, based on individual imaging and tonotopical fit, will give rise to improved outcomes in word recognition compared to the standard fitting. Learning curves will be analyzed by calculating steepness and area under the curve. Normality will be assessed by examining histograms, Q-Q plots, and performing the Shapiro-Wilk test. Depending on whether normality can be established, either nonparametric or parametric statistical comparison tests will be performed. Analysis will be performed in all participants in the intention-to-treat population with separate reporting of participants who are included on a per-protocol basis. Results of these different outcomes will be presented as mean, standard deviation and percentile distribution. In the case of non-parametric testing, results will also be presented as median and interquartile range.

### Ethics

Health risks specifically associated with study participation are limited to exposure to ionizing radiation from cone beam CT. However, participants will be required to devote their time, effort and attention to the study. Specifically, during rehabilitation participants will be trained in two different CI fitting programs instead of one. It is unclear whether this will be a disadvantage or a benefit, as it most likely depends on individual brain plasticity mechanics. From an ethical perspective, however, it can be noted that the possible burden of adapting to two CI fittings simultaneously will be limited to a time window of 12 weeks, after which the effects of wearing two fittings at the same time can be expected to wash out.

## Discussion

The ELEPHANT study combines an individualized cochlear implant fitting strategy with new ideas on clinical trial setups. The study design presented may be regarded as a suitable alternative to the RCT that resolves challenges related to this conventional trial setup (e.g. the number of participants) in this specific setting. By using a daily-randomized crossover setup, our approach aims to remove bias introduced by age, cognition, prior speech performance, pathology and duration of hearing loss, that are present in a normal RCT. Compared to traditional crossover designs, the proposed approach is expected to obtain more information from smaller sample sizes, and to prevent possible first-order carryover effects. In the current protocol, both the efficacy of the trial design and, as an experimental object, a CI fitting strategy based on post-operative imaging will be evaluated. Also, an experimental HA fitting based on loudness perception will be evaluated.

### Study design

Here we propose daily crossover randomization as a strategy to prevent first-order carryover effects due to initial brain plasticity related to the intervention that was given first. Although there is limited neuroimaging research on the cortical reorganization after CI implantation because of technical challenges and safety concerns [66], results of behavioral studies indicate that plasticity occurs predominantly during the first critical period of rehabilitation: the largest performance gain with a CI occurs in the first months of use [33, 67]. Thus, as initial plasticity related to the first intervention has already occurred by the time the second intervention is presented, it is likely that participants are biased towards the first intervention.

One limitation of using daily randomization as proposed here, is that the distribution of exposure to either control or intervention will differ according to each randomization scheme. However, it has been shown that this is not expected to significantly affect the end result as the total duration of exposure is more relevant than the distribution of exposure over time [68]. One could also argue that the absolute learning speed of either mapping approach at the same time will be lower because the exposure is distributed over twice the amount of time, leading to a prolongation of the rehabilitation phase. This is certainly not a given, and the opposite might even be true. Effects of transfer of information from two different maps may increase learning rates in both maps [69]. Interestingly, it has been shown that different transfer functions can be represented simultaneously within the auditory system. In a sound localization experiment, the spectral spatial cues of subjects were disrupted by altering the shape of their pinnae with molds [70]. Although sound localization was initially disrupted, subjects were able to reacquire localization skills while wearing their molds and reach performance levels close to normal. Despite this adaptation, localization accuracy was unaffected in undisturbed ears. Thus, it is apparently possible for the human auditory system to acquire new representations of sound location without interfering with an already existing set. Furthermore, on a neural level it has been shown that task performance can induce rapid plasticity in primary auditory cortex [71–73], indicating that auditory neural processing is flexible and can quickly adapt to changing circumstances. It seems conceivable that similar effects will occur while learning to hear with two different CI fittings.

Importantly, in our design, subjects will not be allocated to the same program for more than 2 days consecutively in the first time-window and no more than 4 days consecutively in the second time-window. The aim of this was to prevent the development of a fixed preference to any program that is given most during the first period of rehabilitation (i.e. either the experimental or standard program). Specifically, based on an analysis of clinical data within Maastricht UMC+ and published studies [32, 35] two distinct time periods have been roughly defined in the learning curve of patients with CI. The first 4 weeks of CI rehabilitation constitute the first time-window as they appear to be characterized by a large improvement in word recognition. Thus, this first period of adaptation can be considered crucial in the learning process. Therefore, a predominance of either the standard or experimental program during this time window will have a major impact on the preference for one or the other. CI rehabilitation tends to show a more flattened learning curve after 4 weeks (second time-window). Thus, by limiting the allocation to the same program to a maximum of 2 days consecutively in the crucial first 4 weeks, and no more than 4 days consecutively thereafter, we avoid the development of a fixed preference based on exposure time.

### Imaging-based fitting

In the experimental condition of this study, frequency allocation settings of the processor will be based on post-operative imaging. Aligning frequency allocations to individual cochlear morphology and electrode positioning has the potential to match electrical stimulation more closely with what the human brain has learned to cope with. Previous attempts to match frequency information to acoustical tonotopical placement have been performed using vocoder setups in people with normal hearing [74] and in patients with experience of wearing CI who had already experienced long-term adaptation to their standard CI settings [75, 76]. To the best of our knowledge, this current study is the first controlled attempt to immediately provide imaging-based frequency mapping to patients with CI at the start of rehabilitation.

Visualization of anatomic structures in the inner ear with clinical imaging methods is a challenging procedure. Although the method of fusing pre-operative and post-operative scans in this study provides relatively high-quality imaging, it remains difficult to determine cochlear landmarks with high precision. Since this is especially the case for the extent of the medial wall, we decided to use the lateral wall to calculate frequency alignment. It can be argued that calculating tonotopical distribution over the cochlear medial wall would lead to a more sensible frequency allocation, as spiral ganglion cells are also located on this side. On the other hand, relative distribution of the lateral wall can be expected to match its projection on the medial wall due to the spiral shape of the cochlea [77].

The imaging-based calculations for each participant will be translated to an experimental CI FAT. In a pilot dataset of 13 patients with CI, it was found that a mean shift of 1.36 octaves (SD 0.56) had to be applied to reach tonotopical alignment (not published). It can be expected that the experimental frequency distributions applied in the current study will deviate to a similar extent from their clinical counterparts (Table 1), with respect to both composition and bandwidth. Since the experimental mapping follows the tonotopical distribution of the cochlea, its composition comes close to a logarithmic accumulation. This is in contrast to the clinical approach, where frequency channels are likely to be partitioned due to their contribution to speech intelligibility. As a result, the general experimental FAT is characterized by broader bandwidths in the low frequencies and narrower bandwidths in the high frequencies compared to most clinical FATs. However, based on current literature it is unclear how these changes in CI frequency tables might affect speech intelligibility and sound perception [76, 78, 79].

A subsequent challenge might arise in case of shallow CI insertion depths and limited cochlear lengths, as this is likely to result in a tonotopical FAT that underrepresents the lowest frequencies. By applying the set of rules as presented in “Methods”, we are attempting to include the full frequency window while still maintaining the philosophy of tonotopical mapping. Since the primary benefit of frequency mapping is to improve speech intelligibility, this rule set is based on the relative contribution of each frequency spectrum to speech understanding [80].

### Bimodal loudness fitting

As a sub intervention in this study, an experimental HA fitting based on loudness perception will be applied in those patients that retain the use of a contralateral HA. Although not the primary focus of this study, the current setup provides an interesting opportunity for experiments in people with bimodal devices. That is, in terms of uniformity, all study participants will be in the same time-window of CI rehabilitation, thus have the same level of bimodal experience, and will also be using the same HA device. By refitting the HA gain in people with bimodal devices based on loudness scaling, we are attempting to match natural loudness perception as closely as possible. Fitting procedures based on individual loudness scaling measurements have been performed previously [81–83], but not in people with bimodal devices. It can be hypothesized that for these patients, a gain prescription based on loudness scaling may make better use of low-frequency hearing thereby be more effective in complementing the CI. However, gain settings based on loudness perception in people with normal hearing might also be experienced as too loud by some patients. Also, as is the case in most CI users, patients have already been accustomed to certain settings for a long period and might have trouble getting acquainted to a fitting based on loudness perception.

### Trial status

At the time of submission of this paper (protocol version number 1.0, 20-01-2020), subject recruitment is still ongoing. Inclusion started in March 2019 and study completion is expected March 2021.

## Supplementary information

**Additional file 1.** Complete research protocol as approved by the ethics committee of the Maastricht University Medical Center (MUMC+).

**Additional file 2.** Case report form.

**Additional file 3.** Time and events schedule.

**Additional file 4.** SPIRIT 2013 checklist: Recommended items to address in a clinical trial protocol and related documents.

## Data Availability

The datasets generated during and/or analyzed during the current study will be made available in a public repository after publication of the primary manuscript.

## Abbreviations

ACALOS: Adaptive categorical loudness scaling
CBCT: Cone beam computed tomography
CI: Cochlear implant
CNC: Consonant-nucleus-consonant
FAT: Frequency allocation table
HA: Hearing aid
HUI-3: Health Utilities Index 3
ICECAP-O: ICEpop CAPability measure for Older people
MUMC: Maastricht University Medical Center
RCT: Randomized controlled trial
SD: Standard deviation
SMRT: Spectral-temporally Modulated Ripple Test
SNR: Signal-to-noise ratio
SPL: Sound pressure level
SRT: Speech reception threshold
SSPIN: Spatial speech perception in noise
SSQ-12: Speech, Spatial and Qualities of Hearing

## References

[1] Wilson BS, Finley CC, Lawson DT, Wolford RD, Eddington DK (1991). Rabinowitz WM. Better speech recognition with cochlear implants. Nature.

[2] Zeng F-G (2017). Challenges in improving cochlear implant performance and accessibility. IEEE Trans Biomed Eng.

[3] Cosetti MK, Waltzman SB (2012). Outcomes in cochlear implantation: variables affecting performance in adults and children. Otolaryngol Clin North Am.

[4] Pisoni DB, Kronenberger WG, Harris MS, Moberly AC (2017). Three challenges for future research on cochlear implants. World J Otorhinolaryngol Neck Surg.

[5] Dorman MF, Gifford RH (2017). Speech understanding in complex listening environments by listeners fit with cochlear implants. J Speech, Lang Hear Res.

[6] Peters JPM, Wendrich AW, van Eijl RHM, Rhebergen KS, Versnel H, Grolman W (2018). The sound of a cochlear implant investigated in patients with single-sided deafness and a cochlear implant. Otol Neurotol.

[7] Nadège C, Valérie G, Laura F, Hélène D-B, Vanina B, Olivier D, et al. The cost of cochlear implantation: a review of methodological considerations. Int J Otolaryngol. 2011;2011:1–13. 10.1155/2011/210838 PMC3199048 22028715

[8] Agabigum B, Mir A, Arianpour K, Svider PF, Walsh EM, Hong RS (2018). Evolving trends in cochlear implantation: a critical look at the older population. Otol Neurotol.

[9] Peterson NR, Pisoni DB, Miyamoto RT (2010). Cochlear implants and spoken language processing abilities: review and assessment of the literature. Restor Neurol Neurosci.

[10] Burns PB, Rohrich RJ, Chung KC (2011). The levels of evidence and their role in evidence-based medicine. Plast Reconstr Surg.

[11] Kendall J (2003). Designing a research project: randomised controlled trials and their principles. Emerg Med J.

[12] Ah-See KW, Molony NC, Maran AGD (1997). Trends in randomized controlled trials in ENT: a 30-year review. J Laryngol Otol.

[13] Gaylor JM, Raman G, Chung M, Lee J, Rao M, Lau J (2013). Cochlear implantation in adults: a systematic review and meta-analysis. JAMA Otolaryngol Neck Surg.

[14] Kraaijenga VJC, Ramakers GGJ, Smulders YE, van Zon A, Free RH, Frijns JHM, et al. No difference in behavioral and self-reported outcomes for simultaneous and sequential bilateral cochlear implantation: evidence from a multicenter randomized controlled trial. Front Neurosci. 2019;13:1–17. 10.3389/fnins.2019.00054 PMC6391354 30842721

[15] van Schoonhoven J, Sparreboom M, van Zanten BGA, Scholten RJPM, Mylanus EAM, Dreschler WA (2013). The effectiveness of bilateral cochlear implants for severe-to-profound deafness in adults: a systematic review. Otol Neurotol.

[16] Cohen NL, Waltzman SB, Fisher SG (1993). A prospective, randomized study of cochlear implants. The Department of Veterans Affairs Cochlear Implant Study Group. N Engl J Med.

[17] Smulders YE, van Zon A, Stegeman I, Rinia AB, Van Zanten GA, Stokroos RJ (2016). Comparison of bilateral and unilateral cochlear implantation in adults: a randomized clinical trial. JAMA Otolaryngol Neck Surg.

[18] Buchman CA, Dillon MT, King ER, Adunka MC, Pillsbury HC, Adunka OF (2014). Influence of cochlear implant insertion depth on performance: a prospective randomized trial. Otol Neurotol.

[19] Dillon MT, Buss E, King ER, Deres EJ, Obarowski SN, Anderson ML (2016). Comparison of two cochlear implant coding strategies on speech perception. Cochlear Implants Int.

[20] Koch DB, Quick A, Osberger MJ, Saoji A, Litvak L (2014). Enhanced hearing in noise for cochlear implant recipients: clinical trial results for a commercially available speech-enhancement strategy. Otol Neurotol.

[21] Riss D, Hamzavi J-S, Selberherr A, Kaider A, Blineder M, Starlinger V (2011). Envelope versus fine structure speech coding strategy: a crossover study. Otol Neurotol.

[22] Willeboer C, Smoorenburg GF (2006). Comparing cochlear implant users’ speech performance with processor fittings based on conventionally determined T and C levels or on compound action potential thresholds and live-voice speech in a prospective balanced crossover study. Ear Hear.

[23] Cleophas TJM (1990). Underestimation of treatment effect in crossover trials. Angiology.

[24] Middlebrooks JC, Bierer JA, Snyder RL (2005). Cochlear implants: the view from the brain. Curr Opin Neurobiol.

[25] Syka J (2002). Plastic changes in the central auditory system after hearing loss, restoration of function, and during learning. Physiol Rev.

[26] Strelnikov K, Marx M, Lagleyre S, Fraysse B, Deguine O, Barone P (2015). PET-imaging of brain plasticity after cochlear implantation. Hear Res.

[27] Fallon JB, Irvine DRF, Shepherd RK (2008). Cochlear implants and brain plasticity. Hear Res.

[28] Kral A, Tillein J. Brain plasticity under cochlear implant stimulation. Cochlear Brainstem Implant. 2006;64:89–108. 10.1159/000094647 16891838

[29] Bottari D, Heimler B, Caclin A, Dalmolin A, Giard M-H, Pavani F (2014). Visual change detection recruits auditory cortices in early deafness. Neuroimage.

[30] Finney EM, Fine I, Dobkins KR (2001). Visual stimuli activate auditory cortex in the deaf. Nat Neurosci.

[31] Landsberger DM, Svrakic Svrakic J, Svirsky M (2015). The relationship between insertion angles, default frequency allocations, and spiral ganglion place pitch in cochlear implants. Ear Hear.

[32] Frijns JHM, Briaire JJ, de Laat JAPM, Grote JJ (2002). Initial evaluation of the Clarion CII cochlear implant: speech perception and neural response imaging. Ear Hear.

[33] Tyler RS, Parkinson AJ, Woodworth GG, Lowder MW, Gantz BJ (1997). Performance over time of adult patients using the Ineraid or Nucleus cochlear implant. J Acoust Soc Am.

[34] Robinson K, Summerfield AQ (1996). Adult auditory learning and training. Ear Hear.

[35] Tyler RS, Summerfield AQ (1996). Cochlear implantation: relationships with research on auditory deprivation and acclimatization. Ear Hear.

[36] Fedorov A, Beichel R, Kalpathy-Cramer J, Finet J, Fillion-Robin J-C, Pujol S (2012). 3D Slicer as an image computing platform for the Quantitative Imaging Network. Magn Reson Imaging.

[37] Johnson H, Harris G, Williams K (2007). BRAINSFit: mutual information rigid registrations of whole-brain 3D images, using the insight toolkit. Insight J.

[38] Dees G, Smits JJ, Janssen AML, Hof JR, Gazibegovic D, van Hoof M (2018). A mid-scala cochlear implant electrode design achieves a stable post-surgical position in the cochlea of patients over time—a prospective observational study. Otol Neurotol.

[39] Dees G, van Hoof M, Stokroos R (2016). A proposed method for accurate 3D analysis of cochlear implant migration using fusion of cone beam CT. Front Surg.

[40] Greenwood DD (1990). A cochlear frequency-position function for several species—29 years later. J Acoust Soc Am.

[41] Macherey O, Deeks JM, Carlyon RP (2011). Extending the limits of place and temporal pitch perception in cochlear implant users. J Assoc Res Otolaryngol.

[42] Nogueira W, Litvak LM, Saoji AA, Büchner A (2015). Design and evaluation of a cochlear implant strategy based on a “phantom” channel. PLoS One.

[43] Saoji AA, Litvak LM (2010). Use of “phantom electrode” technique to extend the range of pitches available through a cochlear implant. Ear Hear.

[44] Friesen LM, Shannon RV, Baskent D, Wang X (2001). Speech recognition in noise as a function of the number of spectral channels: comparison of acoustic hearing and cochlear implants. J Acoust Soc Am.

[45] Byrne D, Dillon H, Ching T, Katsch R, Keidser G. NAL-NL1 procedure for fitting nonlinear hearing aids: characteristics and comparisons with other procedures. J Am Acad Audiol. 2001;12:37–51. 11214977

[46] Scollie S, Seewald R, Cornelisse L, Moodie S, Bagatto M, Laurnagaray D (2005). The desired sensation level multistage input/output algorithm. Trends Amplif.

[47] Devocht EMJ, George ELJ, Janssen AML, Stokroos RJ (2015). Bimodal hearing aid retention after unilateral cochlear implantation. Audiol Neurotol.

[48] Brand T, Hohmann V (2002). An adaptive procedure for categorical loudness scaling. J Acoust Soc Am.

[49] Bosman AJ, Smoorenburg GF (1995). Intelligibility of Dutch CVC syllables and sentences for listeners with normal hearing and with three types of hearing impairment. Audiology.

[50] Houben R, Dreschler WA (2015). Optimization of the Dutch matrix test by random selection of sentences from a preselected subset. Trends Hear.

[51] Theelen-van den Hoek FL, Houben R, Dreschler WA (2014). Investigation into the applicability and optimization of the Dutch matrix sentence test for use with cochlear implant users. Int J Audiol.

[52] Brand T, Kollmeier B (2002). Efficient adaptive procedures for threshold and concurrent slope estimates for psychophysics and speech intelligibility tests. J Acoust Soc Am.

[53] Kaandorp MW, Smits C, Merkus P, Goverts ST, Festen JM (2015). Assessing speech recognition abilities with digits in noise in cochlear implant and hearing aid users. Int J Audiol.

[54] Devocht EMJ, Janssen AML, Chalupper J, Stokroos RJ, George ELJ (2017). The benefits of bimodal aiding on extended dimensions of speech perception: Intelligibility, listening effort, and sound quality. Trends Hear.

[55] Anderson ES, Nelson DA, Kreft H, Nelson PB, Oxenham AJ (2011). Comparing spatial tuning curves, spectral ripple resolution, and speech perception in cochlear implant users. J Acoust Soc Am.

[56] Zhang T, Spahr AJ, Dorman MF, Saoji A (2013). The relationship between auditory function of non-implanted ears and bimodal benefit. Ear Hear.

[57] Aronoff JM, Landsberger DM (2013). The development of a modified spectral ripple test. J Acoust Soc Am.

[58] Gatehouse S, Noble W (2004). The speech, spatial and qualities of hearing scale (SSQ). Int J Audiol.

[59] Noble W, Jensen NS, Naylor G, Bhullar N, Akeroyd MA (2013). A short form of the Speech, Spatial and Qualities of Hearing scale suitable for clinical use: The SSQ12. Int J Audiol.

[60] Furlong WJ, Feeny DH, Torrance GW, Barr RD (2001). The Health Utilities Index (HUI®) system for assessing health-related quality of life in clinical studies. Ann Med.

[61] van Hoof M, Jeuring SFG, Jonkers DMAE, Masclee AAM, Pierik MJ, Stokroos RJ (2016). De Nederlandse vertaling en indruksvalidatie van de ICECAP-A: meten van kwaliteit van leven volgens de capability-benadering. Tijdschr voor gezondheidswetenschappen.

[62] Al-Janabi H, Flynn TN, Coast J (2012). Development of a self-report measure of capability wellbeing for adults: the ICECAP-A. Qual Life Res.

[63] Boretzki M (1999). Quantification of significant sound quality attributes in the context of hearing instrument fine tuning. Phonak Hear Syst Focus.

[64] Cohen J (1992). Quantitative methods in psychology: a power primer. Psychol Bull.

[65] Lerman J (1996). Study design in clinical research: sample size estimation and power analysis. Can J Anaesth.

[66] Stropahl M, Chen L-C, Debener S (2017). Cortical reorganization in postlingually deaf cochlear implant users: intra-modal and cross-modal considerations. Hear Res.

[67] van der Jagt MA, Briaire JJ, Verbist BM, Frijns JHM (2016). Comparison of the HiFocus Mid-Scala and HiFocus 1J electrode array: angular insertion depths and speech perception outcomes. Audiol Neurotol.

[68] Nogaki G, Fu Q-J, Galvin JJ (2007). The effect of training rate on recognition of spectrally shifted speech. Ear Hear.

[69] Li T, Galvin JJ, Fu Q-J (2009). Interactions between unsupervised learning and the degree of spectral mismatch on short-term perceptual adaptation to spectrally-shifted speech. Ear Hear.

[70] Hofman PM, Van Riswick JGA, Van Opstal AJ (1998). Relearning sound localization with new ears. Nat Neurosci.

[71] Fritz J, Shamma S, Elhilali M, Klein D (2003). Rapid task-related plasticity of spectrotemporal receptive fields in primary auditory cortex. Nat Neurosci.

[72] Lee C-C, Middlebrooks JC (2011). Auditory cortex spatial sensitivity sharpens during task performance. Nat Neurosci.

[73] van der Heijden K, Rauschecker JP, Formisano E, Valente G, de Gelder B (2018). Active sound localization sharpens spatial tuning in human primary auditory cortex. J Neurosci.

[74] Ali H, Noble JH, Gifford RH, Labadie RF, Dawant BM, Hansen JHL, et al. Image-guided customization of frequency-place mapping in cochlear implants. In: IEEE International Conference on Acoustics, Speech and Signal Processing (ICASSP); 2015 Apr 19-24; Brisbane (QLD). Piscataway (NY): IEEE; 2015. p. 5843–7.

[75] Başkent D, Shannon RV (2005). Interactions between cochlear implant electrode insertion depth and frequency-place mapping. J Acoust Soc Am.

[76] Fu Q-J, Shannon RV, Galvin JJ (2002). Perceptual learning following changes in the frequency-to-electrode assignment with the Nucleus-22 cochlear implant. J Acoust Soc Am.

[77] Devocht EMJ, Dees G, Arts RAGJ, Smits JJ, George ELJ, van Hoof M (2016). Revisiting place-pitch match in CI recipients using 3D imaging analysis. Ann Otol Rhinol Laryngol.

[78] Faulkner A, Rosen S, Norman C (2006). The right information may matter more than frequency-place alignment: simulations of frequency-aligned and upward shifting cochlear implant processors for a shallow electrode array insertion. Ear Hear.

[79] Fu Q-J, Shannon RV (1999). Effects of electrode configuration and frequency allocation on vowel recognition with the Nucleus-22 cochlear implant. Ear Hear.

[80] Studebaker GA, Sherbecoe RL (1991). Frequency-importance and transfer functions for recorded CID W-22 word lists. J Speech, Lang Hear Res.

[81] Humes LE, Pavlovic C, Bray V, Barr M (1996). Real-ear measurement of hearing threshold and loudness. Trends Amplif.

[82] Kiessling J, Schubert M, Archut A (1996). Adaptive fitting of hearing instruments by category loudness scaling (ScalAdapt). Scand Audiol.

[83] Pastoors AD, Gebhart TM, Kiessling J (2001). A fitting strategy for digital hearing aids based on loudness and sound quality. Scand Audiol.

