# Supplementary material for: Evaluating hearing performance with cochlear implants within the same patient using daily randomization and imaging-based fitting - The ELEPHANT study
Source: Trials. 2020 Jun 23;21:564. doi: 10.1186/s13063-020-04469-x (PMC7310427; doi:10.1186/s13063-020-04469-x)
Supplement: Supplementary file 1 — Additional file 1. Complete research protocol as approved by the ethics committee of the Maastricht University Medical Center (MUMC+). [file 13063_2020_4469_MOESM1_ESM.pdf]

ELEPHANTstudy

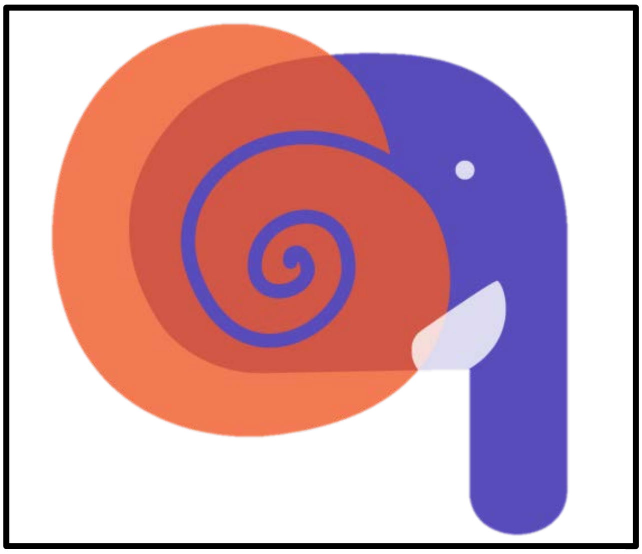

ELEctric

Place-pitched

Hearing

Achieves

Natural

Tonotopy

Illustration designed by Joren Devocht (www.thisisjoren.com)

A single-blinded, daily randomized, internally controlled, cochlear implant intervention trial

(version 3.1, March 2019)

Drs. L.J.G. Lambriks, Master of Human Movement Sciences
Drs. E. M. J. Devocht, Master of Audiology
Drs. J. Debruyne, Master of Audiology
Drs. M. van Hoof, ENT-physician in training
Dr. E. L. J. George, PhD, Medical Physicist-Audiologist

Maastricht University Medical Center+
P. Debyelaan 25
6229 HX Maastricht
The Netherlands

STUDY INFORMATION

**Title**	**"Electric Place-pitched Hearing Achieves Natural Tonotopy"**
**ABR-number**	64874
**METC-number**	
**Short title**	"ELEPHANT"
**Version/Date**	3.1/March 2018
**Coordinating investigator**<br>**Project leader**<br>**Principal investigator**	*Dr. E. L. J. George, PhD, Medical Physicist-Audiologist*<br>Maastricht UMC+<br>ENT/Audiological Centre<br>PO Box 5800<br>6202 AZ Maastricht<br>(T) +31-43-387 79 47<br>erwin.george@mumc.nl
**Researcher**	*Drs. L. J. G. Lambriks, Master of Human Movement Sciences*<br>Maastricht UMC+<br>ENT/Audiological Centre<br>PO Box 5800<br>6202 AZ Maastricht<br>(T) +31-43-387 72 47<br>lars.lambriks@mumc.nl
**Researcher**	*Drs. J. Debruyne, Master of Audiology*<br>Maastricht UMC+<br>ENT/Audiological Centre<br>PO Box 5800<br>6202 AZ Maastricht<br>(T) +31-43-387 27 66<br>joke.debruyne@mumc.nl
**Researcher**	*Drs. E. M. J. Devocht, Master of Audiology*<br>Maastricht UMC+<br>ENT/Audiological Centre<br>PO Box 5800<br>6202 AZ Maastricht<br>(T) +31-43-387 72 47<br>elke.devocht@mumc.nl
**Researcher**	*Drs. M. van Hoof, ENT-physician in training*<br>Maastricht UMC+<br>ENT/Audiological Centre<br>PO Box 5800<br>6202 AZ Maastricht<br>(T) +31-43-387 28 86<br>marc.hoofvan@mumc.nl
**Sponsor**	*azM*<br>P. Debyelaan 25<br>6229 HX Maastricht<br>Nederland<br>(T) +31-43-387 65 43

**Independent physician**	*L.W.J. Baijens, PhD, MD, ENT-physician/surgeon*<br>Maastricht UMC+<br>ENT-department<br>PO Box 5800<br>6202 AZ Maastricht<br>(T) +31-43-387 75 90<br>laura.baijens@mumc.nl
**Statistician**	*A. Miranda L. Janssen, MSc, Assistant Statistical Analyst*<br>Maastricht University<br>Department Methodology & Statistics<br>PO Box 616<br>6200 MD Maastricht<br>(T) +31-43-388 29 54<br>miranda.janssen@maastrichtuniversity.nl
**Collaborating Investigator of Financing party:**	*Dr. Josef Chalupper, PhD, Research Scientist*<br>Advanced Bionics<br>European Research Centre<br>Karl-Wiechert-Allee 3<br>30625 Hannover, Germany<br>(T) +49 511 5700882<br>josef.chalupper@advancedbionics.com

PROTOCOL SIGNATURE SHEET

Name	Signature	Date
**Sponsor or legal representative:**<br>*Maastricht UMC+*<br>P. Debyelaan 25<br>6229 HX Maastricht<br>Nederland<br>(T) +31-43-387 65 43<br><br>Represented by:<br>*Prof. dr. B. Kremer, ENT-physician/surgeon*		
**Coordinating Investigator**<br>**Project leader**<br>**Principal investigator :**<br>*Dr. E. L. J. George, PhD, Medical Physicist-Audiologist*		

TABLE OF CONTENTS

STUDY INFORMATION 2
PROTOCOL SIGNATURE SHEET 3
TABLE OF CONTENTS....................4
LIST OF ABBREVIATIONS AND RELEVANT DEFINITIONS 7
SUMMARY 8
1. INTRODUCTION AND RATIONALE....................11
1.1 Cochlear implantation and bimodal hearing....................11
1.2 Frequency representation: electrical vs. acoustic hearing 11
1.2.1 The relevance of pitch alignment....................11
1.2.2 Tonotopy....................12
1.2.3 Frequency allocation table and mismatch 12
1.2.4 Perceptual pitch matching tasks....................14
1.3 Previous research....................15
1.3.1 Bimodal projects 15
1.3.2 Imaging projects 15
1.3.3 Limitations of current CI study trial designs....................16
1.4 Current proposal 17
1.4.1 General goal 17
1.4.2 Key element 1: Imaging based frequency mapping....................18
1.4.3 Key element 2: Learning curve estimated for test and control by single blinded daily randomization 18
1.4.4 Key element 3: Bimodal acoustic fitting in a CI-like manner based on loudness growth 20
2. OBJECTIVES....................21
3. STUDY DESIGN 22
4. STUDY POPULATION....................25
4.1 Population (base)....................25
4.2 Inclusion criteria....................25
4.3 Exclusion criteria....................25
4.4 Criteria for early termination....................25
4.5 Sample size calculation 26
4.5.1 General principles....................26
5. TREATMENT OF SUBJECTS 29
5.1 Investigational product/treatment....................29
5.1.1 Imaging methods 29
5.1.2 Natural mapping 29
5.1.3 Phantom stimulation 30
5.1.4 Bimodal fitting 30
5.2 Use of co-intervention....................30
6. INVESTIGATIONAL PRODUCT 31

6.1 Name and description of investigational product(s) 31
6.1.1 Hardware 31
6.1.2 Software 32
6.2 Summary of known and potential risks and benefits 32
7. METHODS 34
7.1 Study parameters/endpoints 34
7.1.1 Phase 1: CI fitting 34
7.1.2 Phase 2: bimodal adjustment 35
7.1.3 Phase 3: clinical fit 35
7.2 Randomization, blinding and treatment allocation 36
7.3 Study procedures 38
7.3.1 General information 38
7.3.2 Imaging procedure 38
7.3.3 CI Fitting procedure 39
7.3.4 HA fitting procedure 39
7.3.5 Further clinical rehabilitation 39
7.3.6 Shared decision making and clinical follow-up 40
7.3.7 Test procedures 40
7.4 Withdrawal of individual subjects 42
7.5 Replacement of individual subjects after withdrawal 42
7.6 Follow-up of subjects withdrawn from treatment 42
7.7 Premature termination of the study 42
8. SAFETY REPORTING 43
8.1 Temporary halt for reasons of subject safety 43
8.2 AEs, SAEs and SUSARs 43
8.2.1 Adverse events (AEs) 43
8.2.2 Serious adverse events (SAEs) 43
8.2.3 Suspected unexpected serious adverse reactions (SUSARs) 43
8.3 Annual safety report 43
8.4 Follow-up of adverse events 44
8.5 [Data Safety Monitoring Board (DSMB) / Safety Committee] 44
9. STATISTICAL ANALYSIS 45
9.1 Primary study parameter(s) 45
9.2 Secondary study parameter(s) 45
9.3 Interim analysis (if applicable) 45
10. ETHICAL CONSIDERATIONS 46
10.1 Regulation statement 46
10.2 Recruitment and consent 46
10.3 Objection by minors or incapacitated subjects (if applicable) 46
10.4 Benefits and risks assessment, group relatedness 46
10.5 Compensation for injury 47
10.6 Incentives (if applicable) 47
11. ADMINISTRATIVE ASPECTS, MONITORING AND PUBLICATION 48

11.1 Handling and storage of data and documents 48
11.2 Monitoring and Quality Assurance 48
11.3 Amendments 48
11.4 Annual progress report 48
11.5 Temporary halt and (prematurely) end of study report 48
11.6 Public disclosure and publication policy 49
12. STRUCTURED RISK ANALYSIS 50
12.1 Potential issues of concern - synthesis 50
13. REFERENCES 51

LIST OF ABBREVIATIONS AND RELEVANT DEFINITIONS

**AB**	Advanced Bionics corporation (CI manufacturer)
**AE**	Adverse Event
**Bimodal hearing**	Hearing by two modi in both ears: electrically (through a cochlear implant) in one ear and acoustically (through an acoustic hearing aid) in the contralateral ear.
**BTE**	Behind The Ear: hearing device worn behind the ear
**CBCT**	Cone Beam Computed Tomography
**CI**	Cochlear Implant
**dB**	deciBel
**DSMB**	Data Safety Monitoring Board
**EAS**	Electro-acoustical stimulation in the same ear: combining electrical stimulation (through a cochlear implant) and acoustical hearing (through an acoustic hearing aid) in the same ear.
**ECAP**	The Electrically Evoked Compound Action Potential (ECAP) reflects the response of auditory nerve fibers to stimulation of CI electrodes
**EU**	European Union
**ES**	Effect Size
**FAT**	Frequency Allocation Table
**GCP**	Good Clinical Practice
**HA**	Hearing Aid
**HAP**	Hybrid Analog Pulsatile: coding strategy, available for Advanced Bionics cochlear implants, that delivers temporal fine structure at apical electrodes
**IC**	Informed Consent
**METC**	Medical Ethical Committee (in Dutch: Medisch Ethische Toetsing Commissie)
**NRT**	Neural Response Telemetry
**Phantom**	Sound coding strategy available for Advanced Bionics cochlear implants that is based on current steering and is able to produce a “phantom” pitch percept that extends beyond the pitch range available with the most apical physical electrode.
**(S)AE**	(Serious) Adverse Event
**SOE**	Spread Of Excitation
**SPAN**	Sound coding strategy available for Advanced Bionics cochlear implants to replace inefficient or faulty physical electrodes by using current steering
**SPL**	Sound Pressure Level
**Sponsor**	The sponsor is the party that commissions the organization or performance of the research, for example a pharmaceutical company, academic hospital, scientific organization or investigator. A party that only provides funding for a study is not regarded as the sponsor, but referred to as a financing party
**SUSAR**	Suspected Unexpected Serious Adverse Reaction

SUMMARY

Rationale:

In search of the best possible outcome for the severe hearing impaired who have regained the ability to hear by means of a cochlear implant (CI), electrical stimulation and the information it carries should match as closely as possible to what the human brain naturally has evolved to cope with and learned to process instead of relying on plasticity to adapt to an induced mismatch. At the moment, however, CI's are fitted with a 'one size fits all' principle. This is known to cause a mismatch between the frequencies presented by the CI electrode array and the frequencies represented at the corresponding natural acoustic location in an individual cochlea.

Hypothesis:

In this study it is hypothesized that an individual imaged based fitting that pursues natural hearing alignment and is implemented from the start of the rehabilitation process, will improve the individual outcomes of electric hearing. The natural fitting strategy is thought to give rise to a steeper learning curve, result in a better performance in challenging listening situations, improve sound quality, complement better with residual acoustic hearing in the contralateral ear and win the preference of CI-recipients.

Objective:

To evaluate a new imaging based natural mapping strategy for CI fitting to optimize patient-related outcomes. Compared to conventional clinical fitting, it is proposed that this new method will improve speech understanding and sound perception in adult CI patients during a follow-up period of 6 months.

Study population:

20-30 adults with severe bilateral hearing loss who have previously been approved to receive a cochlear implant in regular care and are willing to use a contralateral hearing aid for the time of the study.

Study design:

This study has multiple phases. The primary part is set up as a prospective single blinded, daily randomized cross-over clinical trial. In this phase electrical hearing will be optimized. When patients retain the use of a contralateral hearing aid, a second phase aims to optimize acoustic hearing. During the third phase, patients receive their clinical fit, which will be based on the preferences they have obtained during the study period. More in detail, the study outline can be summarized as follows.

- Phase 1. During the intensive CI-rehabilitation phase, mapping of the electrical input will be based on an individualized natural frequency alignment as estimated with imaging methods. This natural fitting will be compared to the standard frequency alignment. A daily randomization scheme will be applied whereby the subject crosses over between CI fitting programs and thus effectively acting as his own control, followed by a period of free choice

between both maps to incorporate patient preference. Outcome measures will be assessed at several single points, to address the difference between both CI maps, as well as over time, to address the learning curve with both CI maps.

- Phase 2. After a period of 6 months a stable outcome with CI is expected. When patients retain the use of a contralateral hearing aid up to this time point, the fitting of the acoustic hearing aid will be optimized and compared to the standard fitting. Outcome measures will be assessed acutely and at the end of a take-home period.
- Phase 3. At this time point, patients have indicated their final preference for either the conventional or bimodal HA fitting. In combination with the preferred CI settings, as indicated at the end of phase 1, a clinical fit will be performed for both CI and HA.

Primary study parameter:

To evaluate the effect of natural place-pitched electric mapping, the following outcome measures will be compared between the new fitting strategy under investigation (Test) and the standard clinical fitting (Control):

- Objective primary outcome: degree of speech understanding (words in quiet, sentences in quiet, sentences in noise) with CI during the first 6 months of rehabilitation.
- Subjective primary outcome: patient preference in daily life for either the natural fitting or clinical fitting during the first 6 months of rehabilitation.

Secondary study parameters:

Secondary outcomes include objective and subjective measures to reflect biological response, performance, preference and sound quality.

- Telemetric data on the function of the implant and the response of the auditory nerve.
- Speech understanding with the contralateral HA (acoustic input) and bimodally (CI+HA)
- Extended dimensions of sound perception (spatial masking, listening effort, sound quality, spectral resolution, loudness scaling).
- Quality of life in relation to hearing ability.

Nature and extent of the burden and risks associated with participation, benefit and group relatedness:

There are no substantial health risks specifically associated with study participation. There is one additional cone beam CT scan associated with a low radiation dosage. CE-marked hearing equipment (CI and HA) is used within the scope of standard care. Participation however takes time, effort and attention from subjects. Specifically, patients will train two CI fitting programs over one in standard clinical care. This may either be a disadvantage or a benefit. In case it would be a disadvantage, the risk and burden would be limited to 6 months, after which the effects of the double fitting can be expected to wash out. Patients have to come to the hospital for extra test sessions. As a result of the study, subjects may or may not choose the continued use of the natural fitting strategy of their hearing equipment to improve their performances as far as the standard clinical sound processors support it. In the case of promising results, manufacturer Advanced Bionics might implement the concept of natural fitting to clinical use thereby improving patient-related care in CI rehabilitation.

Recruitment will be performed in a standard pool of CI-patients, thereby making study results directly applicable to clinical care.

1. INTRODUCTION AND RATIONALE

1.1 Cochlear implantation and bimodal hearing

Cochlear implantation (CI) (Figure 1) makes it possible for adults and children with severe sensorineural hearing loss and limited benefit of conventional hearing aids (HA), to regain hearing abilities. By inserting an electrode array in the inner ear, the impaired hair cells of the cochlea are functionally bypassed and direct multi-channel electrical stimulation of the auditory nerve can once again provide information to the auditory system.

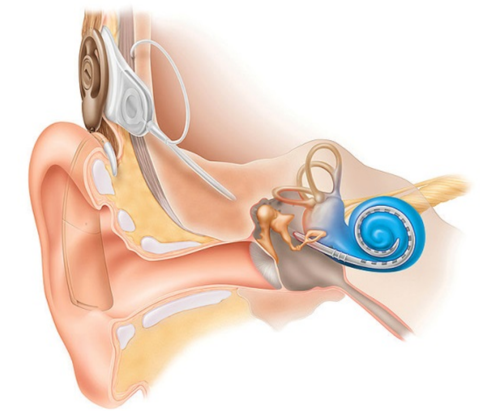

Figure 1. The Cochlear Implant
The cochlear implant is composed out of two parts: an external sound processor and an internal electrode which is placed within the cochlea.

Courtesy of Wikimedia Commons (https://commons.wikimedia.org/wiki/File:Cochleaimplantat.jpg).

The technology of cochlear implants (CI) has been applied successfully since the 1980s. Since the 1990s it is considered to be *standard care* for the severely hearing impaired. In 2016 alone, 527 cochlear implants have been placed in the Netherlands, of which 56 patients received a CI in Maastricht University Medical Center [1].

Box 1. Bimodal hearing
*The combination of a CI and a conventional HA is referred to as '**bimodal hearing**' when combined across two ears.*

In view of the successful results of cochlear implantation in profoundly hearing-impaired people and through technical advances, the indications for a CI have expanded to include severe or sometimes even moderate hearing losses [2,3]. As inclusion criteria for cochlear implantation have become less strict over the years, a trend may be observed towards more residual hearing in the non-implanted ear. This means that in many cases unilaterally implanted patients may benefit from amplification delivered by an acoustical device in the other ear, taking advantage of combining multiple modes: electrical hearing through the CI and acoustic hearing through a contralateral HA (Box 1). These patients have a recent history and on-going utilization of natural acoustic hearing which is known to improve performance and deliver good sound quality [4–7].

1.2 Frequency representation: electrical vs. acoustic hearing

1.2.1 The relevance of pitch alignment

The human brain is known to be an excellent, adaptive and flexible entity in analyzing and combining sound information [8]. In order to achieve a high performance, sound quality and music appreciation [9], a natural and accurate perception of pitch is indispensable. Not only in each ear but also a good pitch alignment between ears seems essential for the binaural system to optimize speech discrimination in noise [10,11] and sound source localization [12] by combining the inputs of both

ears. This binaural cooperation is essential for severely hearing impaired implantees, who have regained the ability to hear by means of a cochlear implant uni- or bilaterally [13], and even more for those who combine electric and acoustic hearing (bimodal listeners) [14]. In the current study proposal, it is hypothesized to be essential to pursue the 'gold standard' of natural hearing if one aims to achieve the best possible outcome. It should thus be a primary requirement that electrical stimulation and the information it carries matches as closely as possible to what the human brain has evolved to cope with and learned to process instead of relying on plasticity to adapt to an induced mismatch which is present for current day CI users [15–17].

1.2.2 Tonotopy

The cochlear position of CI electrodes is important for the perception of pitch. The electrode contacts as displayed in Figure 1 lie at distinct locations within the cochlea as shown in Figure 2. Tonotopic organization specifically relates location within the cochlea to perceived pitch, from the cochlea (Figure 2A) to the brain (Figure 2B). Tonotopy extends to the cortex and is important in auditory perception [18].

Using empirical evidence, Greenwood has quantified endocochlear tonotopy for humans and other species from which place pitch correlation along the basilar membrane can be derived [19]. In order to extend this natural frequency map to be applied in CI users, Stakhovskaya further introduced a spiral ganglion map since this is believed to be the site of excitation in the case of electrical hearing [20]. Nevertheless, today there still exists considerable uncertainty about how tonotopy and brain plasticity are related in the perception of sound, in the physiological situations and in CI users.

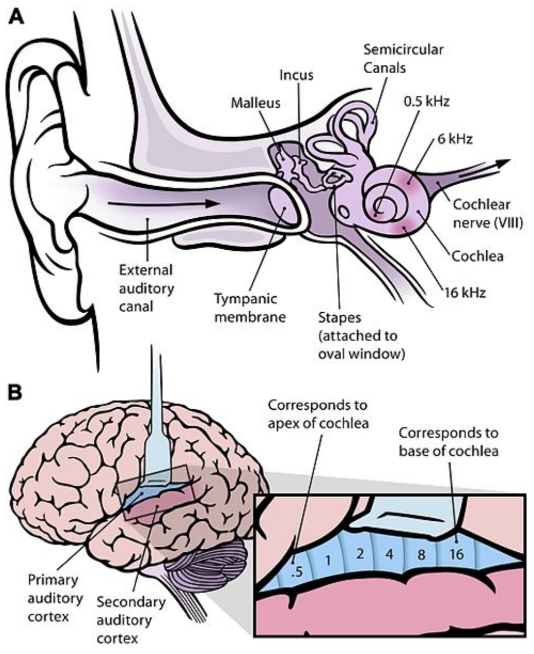

Figure 2. Tonotopy

Locations in a functioning cochlea (place) as shown in ***A*** *(0.5 kHZ – 16 kHz) are related to distinct pitch perceptions. The tonotopy can even be found on a cortical level as displayed in* **B**.

Courtesy of Wikimedia Commons (https://commons.wikimedia.org/wiki/File:Frequency_mapping_in_human_ear_and_brain_-_10.1371_journal.pbio.0030137.g001-L.jpg).

1.2.3 Frequency allocation table and mismatch

In CI sound coding, each electrode on the array is assigned a frequency range in an attempt to deliver tonotopic information, that is, matched as closely as possible to the frequency that is physiologically coded at that location within the cochlea. The map relating a frequency filter of the sound processor's filter bank to an electrode location is known as the frequency allocation table (FAT – Box 2). There are two major differences that limit achieving a theoretical best case scenario.

Box 2. Frequency Allocation Table in CI

*The frequency allocation table (**FAT**) in CI fitting couples the pitch of an input sound to a location of stimulation in the cochlea. In general, the FAT is left at default. During standard fitting no adjustments are made for individual subjects.*

Firstly, since none of the CI electrode arrays are able to reach the most apical regions of the cochlea, there is known to be an offset between the location stimulated by a CI and the location stimulated by the same frequency in a normal cochlea [21].
Secondly, a "one size fits all" fitting principle is applied in commercially available CI systems, using a standard FAT in every CI recipient without adapting the FAT to the individual cochlea. However, it is known that a large variability between cochlear dimensions and resulting CI insertion angles exists between humans [22–24].
Overall this illustrates that with the current standard CI fitting a close alignment of the natural frequency map and the FAT is not yet pursued.

A previous study (manuscript in preparation) performed by our research group was able to shed more light on the extent of induced place-pitch mismatch in the CI-implanted ear. In vivo Cone Beam CT imaging (CBCT), a low radiation variant of CT imaging, was used to calculate the place-pitch estimation per electrode according to Greenwood [19] in individual cochlea's (n=15). This was then compared to the frequency allocation table used to fit the CI electrodes in each patient. As a result we could demonstrate a mismatch (due to both 'offset' and 'non-individual approach') between fitted frequency and natural cochlear place frequency, which ranged between 0 to 3 octaves across patients and electrodes in this population (Figure 3).

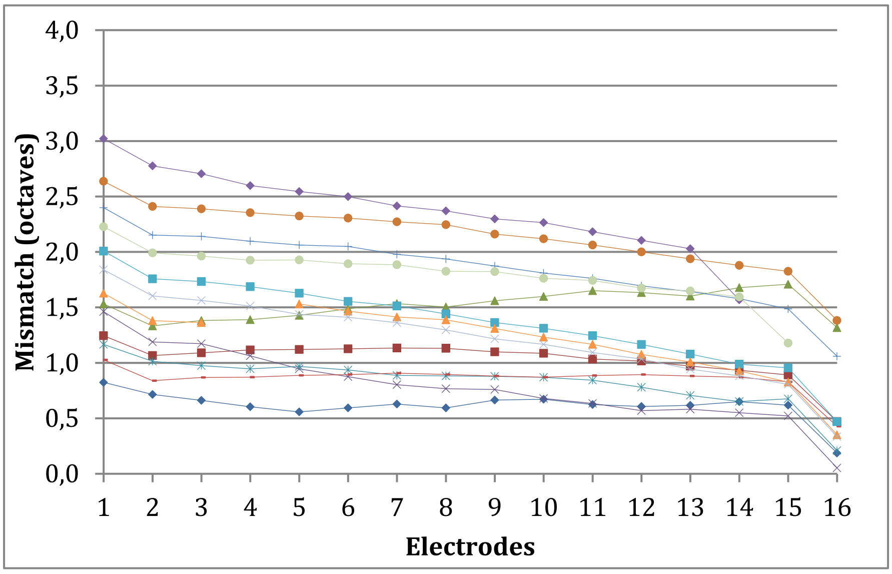

Figure 3 . Electrode mismatches in octaves.

In vivo CBCT imaging was used to calculate the place-pitch estimation per electrode and was compared to the frequency allocation table used to fit the CI electrodes in each patient. As demonstrated, a mismatch between fitted frequency and natural cochlear place frequency differed across patients and ranged from 0 to 3 octaves.

These results along with other data from literature [e.g. 20] strongly advocate for an individual adjustment of the FAT in each CI-recipient. As the cochlea is not a simple standard tube, but a complex individualized 3D structure in which an electrode is placed, it is necessary to adapt the individual pitch allocation to every electrode for each individual and pursue ways to stimulate beyond the most apical electrode. This is illustrated by the example in Figure 4.

**1.** 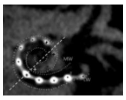 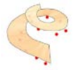	**Phantom Contact**	**Contact 1**	**Contact 2**	**Contact 3**	**Contact 4**	**Contact 5**
**2.** Natural tonotopy	**.25 kHz**	**.5**	**1**	**2.5**	**4**	**7**
**3.** Standard FAT (control)		**.25**	**.5**	**1**	**2**	**4**
**4.** Natural FAT (test)	**+ .25 ←**	**+ .25 →**	**+ .5 →**	**+ 1.5 →**	**+ 2 →**	**+ 3 →**

Figure 4. An example of study methods and intervention for the individual patient

*Using medical imaging (**1.**), the natural tonotopic organization of the cochlea will be quantified for the individual subject (**2.**), the standard FAT (**3.**) will then be adapted (**4.**) to reflect the natural organization of the system.*

Methods for delivering low-frequency information by the CI beyond the maximum insertion depth were recently developed as to partially overcome the fact that CI electrode arrays are unable to reach the most apical region of the cochlea which conveys low frequency information known to improve performance. An example of such a strategy is "Phantom stimulation" [25–27]. This method is based on the current steering capabilities of the Advanced Bionics device. The sound coding strategy uses partial bipolar stimulation, in which current is distributed to two intracochlear electrodes and one extracochlear electrode, to produce a "phantom" pitch percept that extends beyond the pitch range available with the most apical physical electrode. Recent studies in AB CI recipients have investigated the settings of this strategy and demonstrated that the pitch perceived by this phantom electrode is indeed ranked to be lower than the most apical electrode [26,27]. A trend towards better speech recognition was mentioned and questionnaire results showed patient preference when listening to music [28]. This strategy therefore seems promising to be combined with an individualized FAT when fitting CI patients aiming at a natural frequency alignment.

> *Box 3. Phantom stimulation*
>
> *A stimulation strategy beyond the most apical electrode is able to create a "**Phantom**" pitch percept that extends to natural frequencies lower than physically can be reached by the cochlear implant*

1.2.4 Perceptual pitch matching tasks

The perceptual place-pitch correlation in CI-recipients has been investigated in subjective pitch matching experiments asking single-sided deaf patients to match the pitch evoked by a CI electrode to the pitch of an acoustic stimulus played to the contralateral ear with (near) normal hearing.

Most studies only found limited proof of the place-pitch correlation in CI recipients: pitch matching results were generally situated one to two octaves below calculated pitch estimations [28–30] and showed large variability especially beyond the first cochlear turn [31,32]. In an attempt to explain this discrepancy, several theories are suggested, relating to methodological issues with the pitch-

matching procedure [33–35], tonotopic reorganization after CI experience [15,17], the degree of contralateral residual hearing [36] and cross-turn stimulation [28,32,37]. Furthermore it has been demonstrated that when reliable pitch matching results were indeed obtained, they did not deviate consistently from the predictions of the widely-used cochlear frequency-to-place formula of Greenwood [38]. This means that current subjective pitch matching procedures thus do not seem to be a useful tool to reliably estimate the individual place-pitch function and individualize the CI FAT. Therefore, formula based objective methods are warranted and imaging based CI fitting seems to be a promising solution.

The idea for imaging based CI fitting is not new [39]. But the implementation in the clinic, applying imaging to optimize fitting on an individual basis, not only requires the technical know-how, it needs experience with surgery, prospective clinical trials, imaging, neuroscience and modeling to be able to achieve success as there are many factors which might have strongly biased previous results.

1.3 Previous research

1.3.1 Bimodal projects

From 2012 to 2016, our research team has carried out several studies focusing on bimodal patients, both by retrospective reviews and prospective cohort studies. The main goals were to assess the reasons of CI-users whether or not to retain the contralateral hearing aid and to investigate the variation in bimodal benefit across subjects.

First, a retrospective chart review showed that more than 60% of CI-recipients were bimodal users one year after receiving their CI [40]. Hearing aid retention was shown to be associated with more residual speech recognition ability in the non-implanted ear and a smaller difference in speech recognition abilities across both ears. A second study, focusing on self-reported experiences of CI-recipients, showed that bimodal listeners consistently report a bimodal benefit across daily life listening situations [manuscript in progress]. Finally, a test battery was validated and applied assessing speech-in-noise performance, listening effort and sound quality [41]. It was shown that listening effort and sound quality can be regarded as extended dimensions of speech perception, shedding more light on between-patient variability of the benefits provided by complementing a CI with a contralateral HA.

> *Box 4. Previous key findings*
> *If subjects have the ability to use a contralateral HA in combination with a CI, they often do so. The additional HA which acoustically amplifies environmental sounds, improves speech understanding, decreases listening effort and ameliorates the experienced sound quality for bimodal users.*

1.3.2 Imaging projects

Within our research group, we are able to use 3D CI-imaging techniques to estimate a place-pitch map for individual CI recipients. We are able to establish the electrodes in relation to cochlear structures in great detail over the full length of the electrode array. In an exploratory pilot study [42], we have validated this method in a single-sided deaf (SSD) subject who could provide reliable pitch matching results. The electrically evoked pitch percept matched well with the calculated frequency. The mean mismatch in octaves was 0.04 (SD 0.52) in our method in comparison to 0.63 (SD 0.41) using the conventional Stenvers view.

Moreover, we have previously conducted imaging studies on cadavers to study the nervous innervation of the cochlea in micro-grained detail in three dimensions (Figure 5). This allows us to estimate which nervous structures will be electrically stimulated and assess the appropriateness of current models for tonotopy.

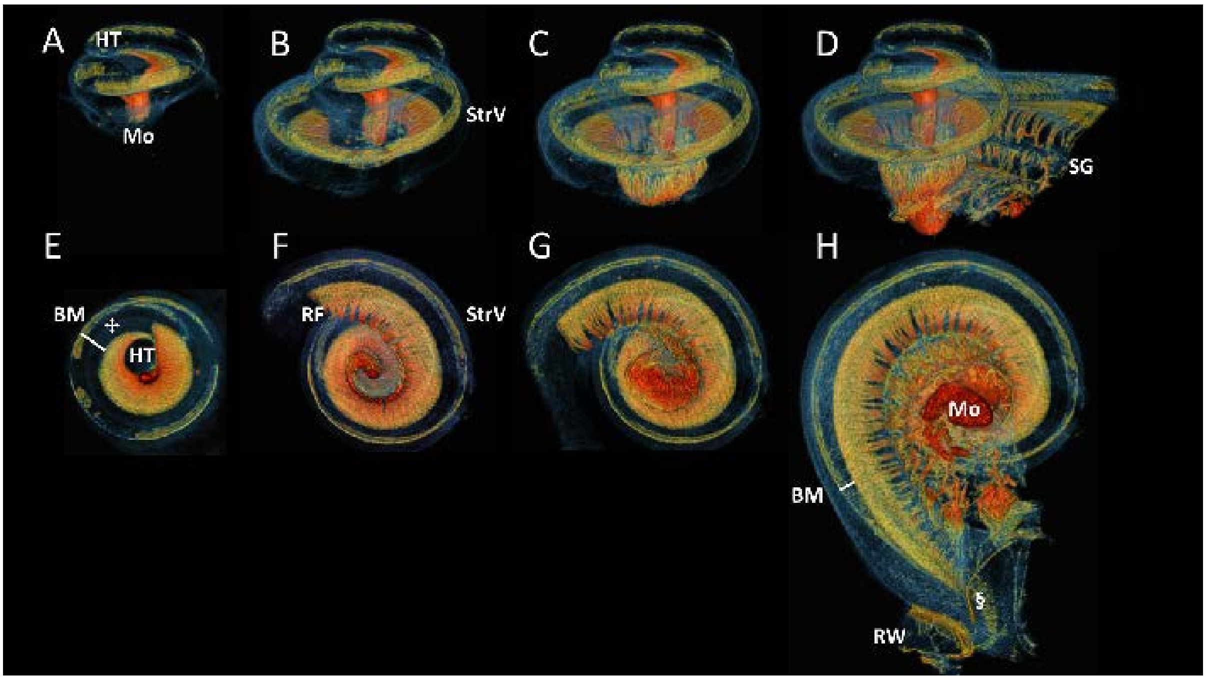

Figure 5. 3D Neural innervation of the human cochlea
A-D show a side view of a cochlea as the volume of interest is iteratively increased. E-H show a corresponding top-down rendering of an increasing volume of interest. Nerve thickness is colored by a red-yelow-green gradient. HT Helicotrema, StrV Stria Vascularis, SG Spiral Ganglion, BM Basement Membrane, Mo Modiulus, RF Radiating Fibers, RW Round Window.
Courtesy of Thomas Van Den Boogert et al.[87]

1.3.3 Limitations of current CI study trial designs

Most audiological related research with CI users is either retrospective in nature or is conducted in a pre-existing population of patients [43]. Those patients have become accustomed to their CI fitting after a process of intensive rehabilitation and have mostly reached a stable steady-state. As mentioned before, the rehabilitation process makes use of standard FATs and relies of the plasticity of the patient’s brain to optimize electrical hearing. After this rehabilitation phase, interventions that change the CI-fitting compete in terms of performance with standard CI fittings to which subjects have already been accustomed.

In the past 25 years that cochlear implantation has become a common clinical intervention, there have been approximately less than 10 prospective RCTs performed [44–46]. Several explanations exist for this low number. Due to the wide range of eligibility criteria for CI users, there is a high variability amongst CI users. Subsequently, CI RCTs need a large number of subjects and hence a multi-center setup is necessary. The technical expertise that is necessary in local sites complicates

performing truly innovative experimental procedures. It is estimated that the rate of progress for CI in terms of performance and sound quality improvements has stagnated [47].

Most importantly, there is a strong irreversible learning effect due to brain plasticity associated with hearing rehabilitation programs. This strongly favors any intervention which is given first during the initial rehabilitation period ('crossover effect' or 'crossover bias'). For example, any Intervention A followed by Intervention B would strongly favor Intervention A as a result of brain plasticity. This restricts the use of a conventional prospective cross-over trial setup and effectively requires a test vs. control setup (Intervention A vs. Intervention B in this example), doubling the amount of necessary participants. A group size for such a setup is often logistically unfeasible with multiple manufacturers competing for the same group of subjects at any center. This also increases the risks for suboptimal treatment and outcomes in a single group.

Hence the challenge in this project was to develop a new trial design to:

1. *Address initial brain plasticity and the critical period for hearing rehabilitation*
2. *Reduce the impact of individual subject characteristic variability*
3. *Decrease the amount of subjects needed to find a moderate statistical effect*
4. *Reduce the duration of a trial*
5. *Allow single-blinding for intervention*

1.4 Current proposal

1.4.1 General goal

This prospective trial will include new CI-recipients, with the primary goal to approach a more natural way of hearing, starting directly at first fit. A stepped individual fitting approach will be applied, with the first months after surgery being dedicated towards optimizing electrical hearing in the CI itself, and afterwards optimizing the acoustical component of the contralateral hearing aid if applicable.

General hypothesis:
An individual fitting that pursues natural hearing alignment and is implemented from the start of the rehabilitation process, will improve the individual outcomes of electric hearing. Natural mapping, based on individual imaging, will enhance performance in challenging listening situations, improve sound quality and therefore win the preference of CI-recipients. Moreover, when natural electric hearing is combined with optimized acoustic hearing in the contralateral ear, additional benefit is expected due to bimodally combined information across ears.

The trial design will feature a single group of CI users which will be prospectively followed from pre-surgery until 12 months post-implantation. The primary test intervention will consist of a change in the FAT fitting which will be compared to the conventional FAT program. As a result of an innovative within-subject randomization scheme, subjects can serve as their own controls. Subjects will be unaware of which fitting is the test intervention and which fitting is the control fitting.

Trial setup
The primary part of the study is set up as a prospective single blinded, daily randomized cross-over clinical trial. In this phase electrical hearing will be optimized. When patients retain the use of a

contralateral hearing aid, acoustic hearing optimization will be performed in a second phase. During the third phase, patients receive their clinical fit, which will be based on the preferences they have obtained during the study period.

1.4.2 Key element 1: Imaging based frequency mapping

One key element for the optimization of natural hearing is thought to be the improvement of frequency mapping by objective imaging methods. It is hypothesized that when CI frequency mapping better approaches natural frequency alignment and coheres more with residual hearing, it will also be easier for patients to adapt to electrical stimulation, the electric and acoustic modalities will be optimally combined, individual performance will be better and the learning curve of their results steeper.

The optimized imaging based method will calculate the individual frequency alignment (Bredberg[48], Greenwood[19], Kawano[49], Escudé[23], Stakhovskaya[20], Kalkman[32], Alexiades[50]) based on postoperative CI-imaging of the scalar location, basal turn diameter, insertion depth and insertion angle. Specific CI fitting features made available by the CI manufacturer Advanced Bionics will be used to implement the natural frequency allocation table (FAT): current steering by HiRes120 [51,52], extended low frequency input dynamic range, Phantom [25–27] and SPAN [53].

Hypothesis 1: *An imaging based frequency allocation that pursues natural frequency alignment can improve processing of auditory information compared to the conventional standard CI fitting in adult CI patients*

1.4.3 Key element 2: Learning curve estimated for test and control by single blinded daily randomization

A topic that has received relatively little attention in literature is the 'learning curve' in CI-recipients, usually as a result of lack of time or power. The problem is that factors such as age, cognition, prior speech performance, pathology and duration of hearing loss all can be expected to influence the learning curve, and specifically its steepness, to a great extent [54,55]. To correct for these factors, we propose to use the subject as its own control in a prospective study whereby two kind of fitting maps are randomized in a daily manner. This daily randomisation scheme will be predefined in an instructional schedule for the subject.

With this unique trial design we set out to measure the relative learning performance of two fitting maps while preventing unwanted bias as a result of whatever map has been given first. This can also be considered to be the most ethical approach since subjects are not expected to have to deal with a suboptimal map for a longer consecutive amount of time once cortical organisation has already occurred [56]. Within CI literature, the proposed study has a unique trial design which to our best search efforts has never been performed before.

An implication of daily randomisation is that subjects are exposed to a map for a minimum of one consecutive day. It has been well established that this is sufficient for attaining a steady learning curve, indeed training sessions of one hour [57] are sufficient to evoke sufficient learning effects.

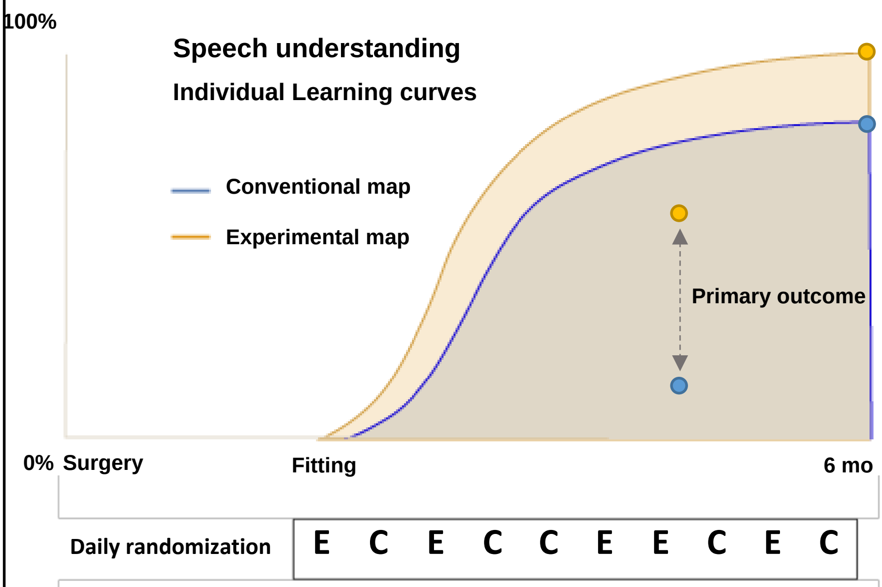

Figure 6. Daily randomization and the difference in learning curve
Study participants will be fitted with two different maps, based on the intervention (conventional vs. experimental FAT, Figure 4), which will be randomized in a daily manner. The difference of performance on speech understanding will be assessed frequently in order to construct the individual learning curves over time. Hereby each subject acts as his own control and subject specific variability is reduced.

Another implication is that the distribution of exposure to a map will differ according to the randomization scheme. However it has been shown [58] that this can't be expected to significantly affect the end result as the total duration of exposure is more relevant than the distribution of exposure over time. One could argue that the absolute speed of learning two maps at the same time will be less because the exposure is distributed over twice the amount of elapsed time leading to a prolongation of the rehabilitation phase. However, this is certainly not a given; indeed the opposite might even be true. Transfer effects of information from the two different maps may increase the learning rates in both maps [59].

Outcome measures will be assessed at several single points, to address the difference between both fitting maps, as well as over time, to address the learning curve with both fitting maps. This daily randomization scheme is unique in its nature and can be regarded as an equivalent, if not better, alternative for the conventional randomized controlled trial (RCT) in this specific setting. The within-patient randomization removes biases due to age, cognition, prior speech performance, pathology and duration of hearing loss, which would still be present in a normal RCT.

Hypothesis 2: *The learning curve is to a great extend affected by the amount of similarity between natural hearing and electrical hearing. When natural hearing is optimized, this is expected to give rise to a steeper learning curve and possibly a higher outcome overall.*

1.4.4 Key element 3: Bimodal acoustic fitting in a CI-like manner based on loudness growth

As our earlier research has shown, over 60% of CI-recipients opt to retain a conventional hearing aid in the contralateral ear [40]. The combination of a CI and a HA in contralateral ears gives access to bimodal benefits on speech understanding, localization, listening effort, balance and sound quality [4–7,60]. There is however no evidence-based consensus on how to optimally fit this bimodal combination [61]. In many cases, the two systems are fitted separately by two independent care providers. As a result, literature shows that the clinically fitted gain in the contralateral hearing aid is not always well tuned [62].

Most CI-fittings are based on finding threshold (T) and most comfortable (M) current levels. In contrast, contralateral hearing aids are often fitted by applying rules which only optimize thresholds. To deliver the best possible naturally individualized outcomes, it may be essential to more closely match CI and HA fitting methods for those subjects who retain a contralateral hearing aid. Specifically, if the T and M level of the HA are fitted similar to CI sound processors procedures, a better loudness match and thus more balanced bimodal fitting might be achieved.

Hypothesis 3: *When fitting of the hearing aid is performed based on the loudness growth experience by taking into account the dynamic range, in a CI-like manner with regard to T- and M-level, an augmented bimodal experience can be achieved.*

2. OBJECTIVES

We propose the evaluation of a natural bimodal fitting rehabilitation program aimed at augmenting individual audiological outcomes in CI-recipients. The program is based on optimizing the electrical component as well as the contralateral acoustic component and their combination within the frequency as well as the loudness domain by using imaging information, extended audiological measurements and unique AB fitting features.

Main objective:

- To evaluate a place-pitched CI fitting method that aims to achieve more natural tonotopic organization in comparison to a conventional CI fitting

***Secondary objectives**:*

- To develop and report on the employed novel trial design to mitigate common CI study limitations and to evaluate the learning curve associated with common parameters as outcome measures

- To develop and report on a new acoustic CI-like fitting approach to augment bimodal performance in the CI + HA combination

These objectives each relate in full to the projects main hypothesis, focusing on an individual fitting that pursues natural hearing alignment and is implemented from the start of the rehabilitation process. We think that natural bimodal mapping will enhance performance in challenging listening situations, improve sound quality and therefore win the preference of multimodal CI-recipients. The new single-blinded, daily randomized, internally controlled electroacoustic intervention trial setup will greatly increase the ability to find a moderate statistical effect of the primary intervention.

3. STUDY DESIGN

Each subject involved in the project will be treated according to the outline below from the moment they receive their implant until 12 months follow-up. This outline can be classified in three different phases. During phase 1, patients will combine their CI rehabilitation with exposure to both the conventional and experimental program based on a daily randomized scheme. This time window is followed by a period of free choice in which patients have the liberty of choosing whatever program they prefer. Those subjects who retain a hearing aid in the contralateral ear will also be enrolled in phase 2, in which optimization of HA fitting will be performed. During phase 3, patients will receive a clinical fit which will be based on the preferences they have obtained during the study period. Patients that will not receive the HA fit in phase 2 will still follow the regular study outline. Data which will be collected during the CI selection procedure will also be included in the study data set. A full overview of the study outline is shown in Figure 6. A graphical representation of the study, which is included in the patient information letter, is shown in Figure 7.

The study design is assessed to be a prospective controlled intervention design with crossover randomization. In a traditional crossover randomized controlled trial, study subjects are allocated to a first treatment arm and then, possibly after a wash-out period, re-allocated to a second intervention phase [63]. However, in this study a new trial design is implemented in which subjects switch between control and intervention phase on a daily basis.

Table 1. Basic outline of the study protocol.

Month	Phase	CI	HA	Intervention	Research question	Method	Result
0-3	Phase 1: CI fitting	Naida CI (research processor)	Naida Link (standard fit)	Standard[1] vs. BEPS+ optimized[2] FAT	Learning curve with CI Benefit optimized FAT	Daily randomization	
3-6					Preference optimized FAT	Free choice	Most used FAT
7-8	Phase 2: bimodal adjustment		Naida Link (M-level fit)	Optimize bimodal HA[3]	Benefit/preference optimized HA	Constant use	Preferred HA-fit
8-12	Phase 3: clinical fit	Naida CI	Naida Link / own HA	Soundwave FAT[4]	Clinical fit just as good?	Constant use	Continue

[1] *Standard FAT:* *frequency allocation table fitted as default based on one size fits all*

[2] *BEPS+ optimized FAT:* *frequency allocation table based on individual imaging fitted per electrode by using BEPS+ software*

[3] *Optimized HA:* *bimodal fitting formula whereby gain is optimized based on M-levels*

[4] *Soundwave FAT:* *frequency allocation table programmed in clinical software Soundwave matched as closely as possible with BEPS+ optimized FAT*

							FIRST PHASE															SECOND PHASE	THIRD PHASE	
**CI**			candidacy	preop	OR	ENT	Daily randomization											Free Choice		Shared decision		Preferred	Clinical fit	Final fit
**HA (contra)**							first fit		REM													Optimize	Choice	
Timeline		# visit code	-	-	-	-	F1	F2	F3	F4	F5	F6	F7	F8	F9	F10	F11	F12	F13	F14	F15	S1	T1	T2
		# weeks			-4	-3	0	1	2	3	4	5	6	7	8	10	12	16	20	26A	26B	30	34	52
		# months									1		1,5				3			6	6	7	8	12
		relative time cfr. previous visit	var	-1M	-	+9D	+4W	+1W	+1W	+1W	+1W	+1W	+1W	+1W	+1W	+2W	+2W	+4W	+4W	+6W		+4W	+4W	+18W
		window cfr. previous visit	var	±1M	-	±3D	±3W	±5D	±5D	±5D	±5D	±5D	±5D	±5D	±5D	±1W	±1W	±2W	±2W	±2W		±2W	±2W	±4W
Clinical rehabilitation		Audiologist (fitting CI/HA)					**150[1]**	**75[2]**	**90[3]**	**75[2]**			**75[2]**				**60[2]**			**45[4]**		**60[5]**	**60[6]**	**60[7]**
		Speech therapist (rehab)						45	45	45	45	45	45	45	45	45	45							
		Social worker (consult)									60									60				60
Audiometry	unaided	Thresholds + CNC	20	20			**20**													**20**				20
	HA	Aided thresholds	10[E]						**5[E]**											**5[E]**			**5[C]**	5[D]
	CI	Aided thresholds							**10[A]**				**10[A]**							**5[A*]**				5[D]
REM	HA	Aided	15						**15**															
Imaging		CT/MRI	x																					
		CBCT				**x**																		
CI-data		M-T levels					0	0	0				0				0			0				
		Datalogging						0	0	0	**2**	**2**	0	**2**	**2**	**2**	**0**	**2**	**2**	0		**0**	0	0
		Impedances			x		0	0	0	0	**2**	**2**	0	**2**	**2**	**2**	**0**	**2**	**2**	0		**0**	0	0
		Cross-impedances			**x**			**0**							**0**					**0**				
		ECAP			**x**			**20**							**20**					**20**				
Basic tests	CI	CNC					**10[A]**	**10[A]**	**10[A]**	**10[A]**	**10[A]**	**10[A]**	**10[A]**	**10[A]**	**10[A]**	**10[A]**	**10[A]**	**10[A]**	**10[A]**		**5[A*]**	**5[B]**	**10[C,D]**	**5[D]**
	CI	Matrix quiet					**20[A]**	**20[A]**	**20[A]**	**20[A]**	**20[A]**	**20[A]**	**20[A]**	**20[A]**	**20[A]**	**20[A]**	**20[A]**	**20[A]**	**20[A]**		**10[A*]**	**10[B]**	**20[C,D]**	**10[D]**
	CI	Matrix noise					**20[A]**	**20[A]**	**20[A]**	**20[A]**	**20[A]**	**20[A]**	**20[A]**	**20[A]**	**20[A]**	**20[A]**	**20[A]**	**20[A]**	**20[A]**		**10[A*]**	**10[B]**	**20[C,D]**	**10[D]**
	HA	CNC	15[E]				**5[E]**		**5[E]**	**5[E]**						**5[E]**					**5[E]**	**5[B]**	**5[C]**	**5[D]**
	CIHA	CNC								**10[A]**						**10[A]**					**5[A*]**	**5[B]**	**5[C]**	**5[D]**
	CIHA	Matrix noise								**20[A]**						**20[A]**					**10[A*]**	**10[B]**	**10[C]**	**10[D]**
Summed up estimated time basic tests			*15*				*55*	*50*	*55*	*85*	*50*	*50*	*50*	*50*	*50*	*85*	*50*	*50*	*50*		*45*	*45*	*70*	*45*
Extended tests	CI + CIHA	Matrix noise spatial														**80[A]**					**40[A*]**	**40[B]**	**40[C]**	
	CI + CIHA	Listening effort									**24[A]**						**24[A]**				**12[A*]**	**12[B]**	**12[C]**	**12[D]**
	CI + HA	Loudness scaling									**18[A]**						**18[A]**				**12[A*]**	**12[B]**	**12[C]**	
	CI	SMRT									**20[A]**						**20[A]**				**20[A*]**		**10[C]**	
Questionnaires		SSQ-12		x																A*			C	D
		HUI-3		x																A*			C	D
		ICECAP-A		x																A*			C	D
		Preference scales					A	A	A	A	A	A	A	A	A	A	A	A	A	A*			C	D
		Sound quality								A							A			A*			C	D
Compliance checks		CI								x			x				x							
		HA											x							x				
Trial administration		Adverse events			x	x	x	x	x	x	x	x	x	x	x	x	x	x	x	x	x	x	x	x
		Extended adverse events					x											x				x	x	x
		Data management check-up					x											x				x	x	x
		Device deficiencies			x	x	x	x	x	x	x	x	x	x	x	x	x	x	x	x	x	x	x	x
Invoice travel expences																					x			x
Total estimated time (in minutes) per visit (excluding questionnaires and imaging)			60	20	NA	NA	225	190	220	205	217	99	180	99	119	214	217	54	54	119	165	169	199	207
Reference time standard clinical rehabilitation			*60*	*20*	*NA*	*NA*	*120*	*105*	*120*	*105*	*105*	*45*	*120*	*45*	*45*	*45*	*45*	*0*	*0*	*195*		*0*	*0*	*195*

var = variable moment
extra visit (not combined with clinical visit)
deviant from clinical rehabilitation (e.g. additional test, visit takes more time)

Figure 6. Full overview of study outline.

LEGEND

Test conditions

A = measure outcome with standard AND natural FAT (HA = bimodal fit)
A*= measure with preferred FAT (standard OR natural (HA = bimodal fit)
B = measure with HA loudness fit in acute setting (CI=preferred FAT)
C = measure with HA loudness fit after acclimatisation (CI=preferred FAT)
D = measure with CI and HA in clinical fit
E = *candidacy* **:** measure both hearing aids seperately and in case of a bilateral HA user also bilateral for CNC; *postop* **:** measure HA contra
0 = time included in fitting CI

Test	testing properties	# administrations per testing property	Estimated time
**Thresholds**	250-8000Hz	1x	5 min per ear/condition
**CNC**	55 dB, 65 dB, 75dB	1x (retest 65 dB)	5 min per ear/condition
**Matrix Quiet**	65dB	2x (test-retest)	10 min per condition
**Matrix Noise**	N65dB, Svariable	2x (test-retest)	10 min per condition
**Matrix noise spatial**	S0NCI, S0NHA	2x (test-retest)	20 min per condition
**Listening effort**	6 SNR	5x per SNR	6 min per condition
**Loudness scaling (Acalos)**	4 noise frequencies	1x	6 min per condition
**SMRT**	Frequency selectivity test	2x (test-retest)	10 min per condition

Fitting legend

1 Initial CI-fitting in week 1 according to clinical practices, including impedance measurement and determination of M-T levels, subsequently initial fitting of HA contra. In case of ipsilateral residual hearing: try-out EAS live and decide whether or not to continuate study.
2 CI-fitting according to clinical practices, including impedance measurement, datalogging and determination of M-T levels, followed by fitting of HA contra
3 CI-fitting according to clinical practices, including impedance measurement, datalogging and determination of M-T levels, followed by extensive fitting of HA contra based on REM
4 CI evaluation fitting according to clinical practices, including impedance measurement, datalogging and determination of M-T levels, followed by evaluation of HA fitting
5 HA optimization fitting, CI fitting limited to check of impedances and datalogging
6 Programming of preferred HA fitting at the end of testing, programming of preferred CI fitting with Soundwave instead of BEPS+
7 CI evaluation fitting according to clinical practices, including impedance measurement and datalogging, followed by evaluation of HA fitting

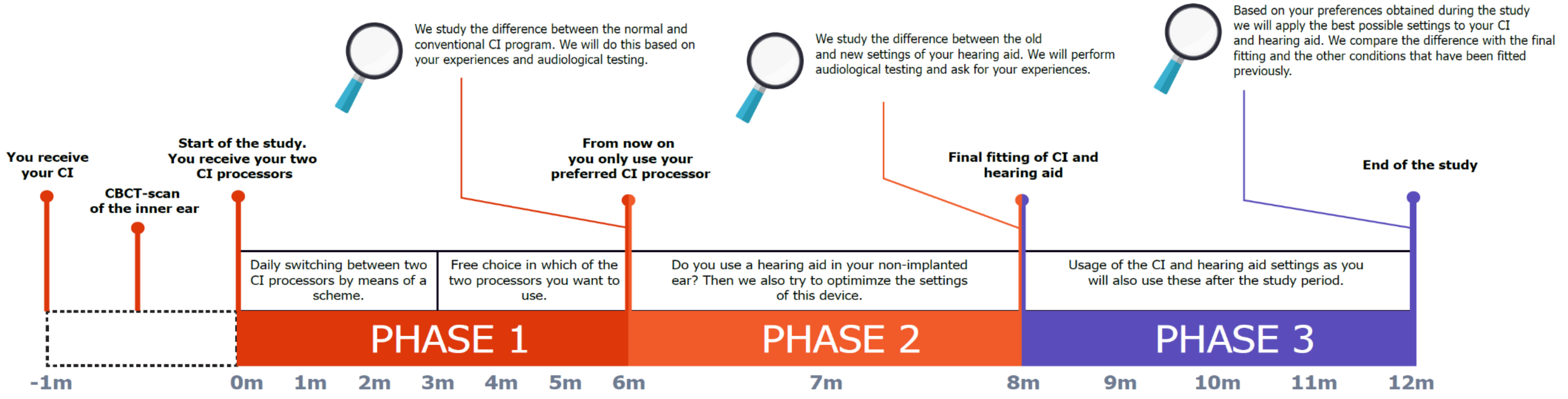

Figure 7. Graphical overview of study outline as enclosed in patient information (translated from Dutch).

4. STUDY POPULATION

4.1 Population (base)

Subjects who meet the inclusion criteria will be recruited from the adult patient, CI candidate population at the Maastricht University Medical Center+. All patients included in the study undergo a thorough selection procedure at the Maastricht University Medical Center+ in which they will be qualified for receiving a unilateral cochlear implant, based on the medical, audiological and psychosocial expertise of our CI team. During and at the end of the selection procedure patients will be informed about the possibility of participating in the study, as will be explained in section 10.2.

4.2 Inclusion criteria

In order to be eligible to participate in this study, subjects must meet all of the following criteria:

- Adult (18y or older) and meeting the conventional Dutch CI criteria;
- Proficient speaker of Dutch language;
- Post-lingual onset of profound deafness (> 4 years of age);
- Subject receives an Advanced Bionics implant with Midscala electrode and an Advanced Bionics sound processor;
- Prepared to use study specific hearing aid (Phonak) for the duration of the study (in case of HA-use in the non-implanted ear);
- Rehabilitation at MUMC+ for the first year after surgery regarding CI as well as HA;
- Active participation in trial related procedures such as daily randomization and regular testing.

4.3 Exclusion criteria

A potential subject who meets any of the following criteria will be excluded from participation in this study:

- Physical or non-physical contraindications for MRI or CT imaging;
- Additional disabilities that may prevent active participation and testing as per protocol. If there are indications that the mental abilities to comply with the study procedures are insufficient, additional screening will be performed with the Mini-Mental State Examination. Patients will be excluded from the study when the resulting score is lower than 24;
- Cochlear or neural abnormalities that could affect outcome measures and/or compromise the placement of the electrode as assessed by the CI surgeon;
- Active participation in another prospective clinical trial;
- Pregnancy at time of imaging;
- Requirement for electric-acoustic activation prior to the first year follow-up;
- Having received a cochlear implant earlier (e.g. explantation or bilateral implantation).

4.4 Criteria for early termination

In the first three months of the study, patients have to wear their CI according to their randomization schedule. Also, over the course of the first six months the contralateral HA should be worn sufficient in order to be included for the experimental HA fitting. Whether subjects comply to these demands will be monitored and then compared with cut-off points.

In case of severe non-compliance with randomization procedures or appearance of exclusion criteria during the study, subjects will be transferred to standard clinical care and outstanding adverse events will be reported to the responsible audiologist and/or clinician. These subjects will be removed from the intention to treat population. In case of less severe randomization deviations, subjects will be treated as major protocol deviators and will be removed from the per protocol population. Subjects will also be removed from the study population when no more than 8 channels (excluding the phantom channel) can be mapped below 8598 Hz. This may be due to shallow insertion of the CI electrode array or other complications.

Patients will report the amount of time during which each they has used either the standard or the natural program. By comparing these values to the ratios in the randomization schedule, a compliance difference can be calculated over each time window. In Table 2 it is shown to what extent subjects can show non-compliance to the randomization schedule before consequences take effect.

Table 2. Criteria for early termination in case of non-compliance with randomization procedures.

Classification of non-compliance	Cut-off value		
Compliance	≤15%		
Minor non-compliance	>15%		
Severe non-compliance	>25%		
Extreme non-Compliance	>40%		
	Visit F4	Visit F7	Visit F11*
Study continuation			
Removed from per protocol population		When 2x >15%	When 2x >15%
Transferred to clinical care	When >40% x 1	When 2x >25% or >40% x 1 or 1x total usage time <360 min/d	When 2x >25% or >40% x 1 or 1x total usage time <360 min/d

* The compliance result that is calculated on visit F11, and includes the time window between F7 and F11, counts double since its time window is also double as long as compared to the results calculated on visits F4 and F7.

4.5 Sample size calculation

4.5.1 General principles

Complementary to the new study design for clinical trials presented in this paper, the research group also has given thought on a different method for a priori sample size and power calculation. Instead of calculating the estimated amount of study subjects needed, one could also work the other way around. After all, many clinical trials depend on the supply of patients within a selective pool and don't have any additional recruiting possibilities. This study is no exception on that point.

The project will aim to include a minimum sample of 20 subjects. If study inclusion is prosperous, up to 30 subjects will be included. For analysis of further a priori calculation the following factors need to be in consideration:

- Significance level. A frequently used alpha level (type I error) in determining the sample size in academic research studies is 0.05 [64] with a corresponding Z-score of 1.645 (one-sided) or 1.96 (two-sided).
- Power. Many studies aim for a power of 80% with a corresponding Z-score of 0.84.
- Variance. The variance or standard deviation is often obtained either from previous studies or from a pilot study.
- Effect size. As a value for the expected difference (minimum detectable difference) it is useful to use the effect sizes found in prior studies. Where no previous study exists, the expected difference can be determined from literature review, logical assertion, and conjecture.
- Type of statistical test. Sample size calculations are different for specific types of statistical tests.

Sample size calculation is based on the primary study outcome, which in this study corresponds to the difference in speech understanding with CI between the conventional and experimental settings after 6 months of rehabilitation. Since there is no data available for power calculation, a number of assumptions must be made using the concept of Cohen's d [65,66]. The proposed statistical test concerns a paired t-test. The required sample size is:

$$N = ((z_{\alpha/2} + z_{1-\beta})^2 * \sigma^2_{difference}) / \mu_{difference}^2$$

The above formula is based on a normal z-distribution [67]. To adapt to a t-distribution the required sample size has to be increased by 2 [68]:

$$N = 2 + ((z_{\alpha/2} + z_{1-\beta})^2 * \sigma^2_{difference}) / \mu_{difference}^2$$

Since Cohen's d is defined as: $\sigma_{difference} / \mu_{difference}$, the sample size formula can be rewritten as:

$$N = 2 + ((z_{\alpha/2} + z_{1-\beta})^2 / d^2)$$

From this formula the ES can be calculated as:

$$d = \sqrt{}(2 + ((z_{1-\alpha/2} + z_{1-b})^2)/n)$$

Using these formulas, it is possible to construct multiple tables using variations of all the different factors. For example, Table 3 shows the different variations in effect size with a sample size range of 20-30 subjects, a significance level range of 0.01-0.08 and a given power of 80%. These results show that with a type 1 error of 5% and a minimum sample size of 20 an effect size of 0.70 can be detected. If 30 study subjects will be recruited and alpha level is kept similar, effect size is 0.57.

Table 3. Calculated effect sizes for variations of sample size and significance level for a given power of 80%.

	Power	80%	80%	80%	80%	80%	80%	80%	80%
		**Significance level**							
		0,08	0,07	0,06	0,05	0,04	0,03	0,02	0,01
**Sample size**	20	0,66	0,67	0,69	0,70	0,72	0,74	0,78	0,73
	21	0,64	0,66	0,67	0,68	0,70	0,73	0,76	0,71
	22	0,63	0,64	0,65	0,67	0,69	0,71	0,74	0,69
	23	0,62	0,63	0,64	0,65	0,67	0,69	0,72	0,68
	24	0,60	0,61	0,63	0,64	0,66	0,68	0,71	0,66
	25	0,59	0,60	0,61	0,63	0,64	0,67	0,69	0,65
	26	0,58	0,59	0,60	0,62	0,63	0,65	0,68	0,64
	27	0,57	0,58	0,59	0,60	0,62	0,64	0,67	0,62
	28	0,56	0,57	0,58	0,59	0,61	0,63	0,66	0,61
	29	0,55	0,56	0,57	0,58	0,60	0,62	0,64	0,60
	30	0,54	0,55	0,56	0,57	0,59	0,61	0,63	0,59

5. TREATMENT OF SUBJECTS

5.1 Investigational product/treatment

Patients will be treated according to the outline in section 3 (Figure 6) from the moment they receive their implant until 12 months after their processor has been activated for the first time. For those subjects who retain a hearing aid in the contralateral ear the experimental HA fitting will be performed as well. The devices used in this study are recognized as standard clinical instruments in hearing rehabilitation care. Technical specifications and product information on hardware and software is provided in section 6.1 of this document and section D of the research dossier.

5.1.1 Imaging methods

To accurately identify the positioning of implanted CI electrodes in the human cochlea, it is necessary to subject patients to high quality imaging. In this study a fusion method of pre-operative CT and/or MRI and post-operative CBCT imaging will be used. Patients will have a standard clinical CT and/or MRI before their CI will be implanted and one week after implantation a CBCT scan will be made. Images will then be fused using 3D Slicer and BRAINSFit software. As validated in previous routine [69], this procedure generates the high quality imaging needed for detailed intra-cochlear electrode assessment.

5.1.2 Natural mapping

Based on pre- and postoperative imaging, it will be possible to identify intra-cochlear electrode positioning in each individual patient. By comparing the position of the electrode array with the natural tonotopic organization of the cochlea, an experimental frequency allocation program will be created which pursues natural frequency alignment. In Soundwave (latest version), the standard clinical fitting software for CI's manufactured by Advanced Bionics, frequency allocation tables are set to default and there are limited possibilities for adjustment. Also, phantom stimulation is not possible. Therefore, the research software BEPS+ will be made available by Advanced Bionics to enable natural mapping. Since BEPS+ is not a CE-marked product, more details of this software package are described in section 6 and a full Investigational Medical Device Dossier is enclosed in section D of the dossier.

Patients will receive two processors, one containing the conventional FAT and one with the experimental FAT, at their first CI fitting consultation and they will be instructed to use both programs based on a daily randomization scheme. Compared to conventional standard CI fitting, it is proposed that natural mapping will enhance processing of auditory information and improve outcomes of electrical hearing. Further CI rehabilitation will be in correspondence with regular clinical routine, as can be assessed from the outline in Figure 6. In phase 3 of the study patients will exchange their loaner processor (section 6.1) for their own processor, which will then be programmed with CE-marked clinical fitting software (Soundwave). If the patient preferred the settings of the conventional map, these can be easily transferred to the new processor without any differences. When the experimental map is preferred, as created by BEPS+ during the first stage of the project, the map will be translated as closely as possible to the clinical software mapping in Soundwave. The difference between both research and clinical map will be monitored and evaluated during the last visit at 12 months.

5.1.3 Phantom stimulation

Phantom stimulation is a method to deliver low-frequency information beyond the most apical electrode of a cochlear implant [25–27]. This method can overcome the fact that CI electrodes are unable to reach the low frequency apical region of the cochlea. Since this strategy seems promising to be used in fitting CI patients when aiming at a natural frequency alignment, it will be enabled in the experimental FAT. Phantom stimulation is not possible in the standard Soundwave software; therefore BEPS+ will be used.

5.1.4 Bimodal fitting

For those subjects who have aidable residual hearing in the contralateral ear (Fletcher Index <100) the hearing aid Naída Link (by Phonak) will be fitted to that ear according to normal clinical routine. Six months after initial activation, the subgroup of patients that chooses to retain the hearing aid will enter phase 2 of the study and receive experimental bimodal fitting. This means that the hearing devices of these patients will be fitted in a CI-like manner with regard to T- and M-level. It is proposed that an augmented bimodal experience can be achieved, thereby improving naturally individualized outcomes. This fitting procedure will be performed with Target (latest version), which is the regular clinical software for Phonak's hearing aids.

In phase 3 of the study patients get to decide whether to retain the Naída Link hearing aid or return to their own hearing aid which they had prior to receiving the CI. If they prefer to retain the Naída Link, the clinical procedure for renewing the hearing aid by the health insurance reimbursement (each 5 years) will be started. If the renewing period is not expired yet, an alternative deal with the manufacturer will meanwhile be established in order to continue the preferred audiological treatment in relation to ethical and regulatory requirements.

5.2 Use of co-intervention

Patients are requested not to let any other party (hearing aid dispenser or audiological centre) change the settings of their CI and hearing aid during the course of the study. Due to possible interactions on the study outcomes it is also not allowed to use electric acoustic stimulation (EAS).

6. INVESTIGATIONAL PRODUCT

6.1 Name and description of investigational product(s)

As described in section 5.1, all devices used in this study are CE-marked and recognized in standard clinical practice (Table 4). The instruments will be used within their indication in normal clinical hearing rehabilitation and not in combination with other products. For CI fitting, non-CE marked research software will be used in conjunction with regular clinical software. HA fitting will be performed exclusively with clinical software. Technical specifications, CE-mark information and a hazard-analysis for hardware and software can be consulted as part of the product information in section D of the dossier.

Table 4. CI = cochlear implant, HA = hearing aid, * = CE marked

Product category		Product name	Manufacturer
CI	Hardware	HiRes Ultra 3D implant*	Advanced Bionics
		Naida CI processor*	Advanced Bionics
	Software	Soundwave (latest version)*	Advanced Bionics
		BEPS+	Advanced Bionics
HA	Hardware	Naida Link*	Advanced Bionics
	Software	Target (latest version)*	Phonak

6.1.1 Hardware

All patients included in the study will receive unilateral implantation with a HiRes Ultra 3D implant after a standard CI operational procedure. This implant is developed by the manufacturer Advanced Bionics and is currently used in clinical practice by the Maastricht University Medical Center+. It is the successor of the HiRes 90K implant, which was used previously. To avoid bias, there will not be used any other implant than the Ultra 3D within the study population, as also corresponds to current clinical practice. One month after implantation, patients will receive a Naída processor as a loaner. This device is connected to the implant through a magnetic coil. Patients will be provided with the newest device in the Naída product line, as would be the case with patients in regular clinical routine choosing for an Advanced Bionics CI. However, since research software is needed to apply experimental frequency conditions and phantom stimulation (section 6.1.2), the processors used as loaners in the study will be programmed as research processors. This provides the additional features necessary for the study.

In the contralateral ear patients will be equipped with a loaner Naída Link hearing aid from Phonak. The Naída Link is a behind-the-ear hearing aid which is used in regular clinical routine. This hearing aid is designed to deliver the optimal bimodal hearing situation in combination with the Naída CI speech processor. Both CI and HA can be matched in terms of sound processing, volume behavior

and programming alerts. If patients choose to retain their Naída Link after the study, both conventional and experimental settings can be programmed according to the patients' preference.

6.1.2 Software

The regular clinical software package for fitting of Advanced Bionics' CI's is Soundwave (latest version). However, since Soundwave does not provide the full range of possibilities to adjust frequency allocation tables and activate phantom stimulation, BEPS+ research software will be used for these purposes. Advanced Bionics will provide a specific research laptop which includes BEPS+ and has instructed researchers on how to properly use the software. Since BEPS+ is not CE-marked, an Investigational Medical Device Dossier of this software will be included in section D of the dossier. Furthermore, the research team will contact instrumental services of the Maastricht UMC+ and establish a software licence agreement. Hearing aid fitting will be done with Target (latest version), which is the clinical and CE marked software of the manufacturer Phonak. During phase 3 of the study, patients exchange their loaner processor for their own Naída CI processor. At this point, Soundwave will be used instead of BEPS+ to match clinical routine.

6.2 Summary of known and potential risks and benefits

It is hypothesized that an individual fitting pursuing natural hearing alignment which is implemented from the start of the rehabilitation process, will have potential benefits for CI patients. The natural fitting strategy is thought to improve outcomes of electrical hearing, giving rise to a steeper learning curve, result in a better performance in challenging listening situations, improve sound quality and complement better with residual acoustic hearing in the contralateral ear.

There are no known health risks associated with participation in this study. CE-marked hearing equipment (CI and HA) is used within the scope of standard care. Participation however takes time, effort and attention from subjects. Patients have to come to the hospital for extra test sessions. They also need to comply to a daily randomization scheme and need to be prepared to use a research processor for their CI, which may have impact on the connectivity with assistive listening devices.

Another implication may be that the distribution of exposure to either the conventional map or experimental settings will differ according to the randomisation scheme. However, it has been shown [58] that this can't be expected to significantly affect the end result as the total duration of exposure is more relevant than the distribution of exposure over time. One could argue that the absolute speed of learning two maps at the same time will be less because the exposure is distributed over twice the amount of elapsed time leading to a prolongation of the rehabilitation phase. However, this is certainly not a given; indeed the opposite might even be true. Transfer effects of information from the two different maps may increase the learning rates in both maps [59].

Also, if a patient happens to prefer the experimental CI fitting, it may not be possible to provide the same settings in the speech processor that will be used during phase 3 and after the study. Patients will then exchange their research processor for their own commercial processor and fitting will be done with Soundwave (latest version) instead of research software. Since it is impossible to alter frequency allocation tables with the same resolution and activate phantom stimulation in regular processors and software, patients may be confronted with a difference in hearing performance

when the study is finished. If this is the case, the experimental fitting settings will be mimicked as closely as possible with regular software. Implementation in clinical software will be explored by the manufacturer in case of substantial benefits found in this study.

7. METHODS

7.1 Study parameters/endpoints

7.1.1 Phase 1: CI fitting

Main study parameter/endpoints

The main premise of this study is that an individual CI fitting with natural hearing alignment will improve the outcomes of electrical hearing. It is thought that natural fitting will win the preference of CI-recipients, give rise to a steeper learning curve and result in better speech understanding. These effects will be evaluated in phase 1, of which the primary outcome measures are shown in Table 5. Comparisons will be made between the new fitting strategy under investigation and the standard clinical fitting.

Table 5. Phase 1: primary outcome measures.

Research question	Outcome measure	Measurement method	Timing of measurement(s)
Fitting preference in daily life	Patients satisfaction	Satisfaction scale	At every fitting consultation
Speech understanding	Word score in quiet	Dutch Consonant Nucleus Consonant test	See overview in section 3 (Figure 6)
	Sentence score in quiet	Dutch matrix sentence test in quiet condition	See overview in section 3 (Figure 6)
	Sentence score in noise	Dutch matrix sentence test in noise condition	See overview in section 3 (Figure 6)

Secondary study parameters/endpoints

It is hypothesized that natural fitting will also improve sound perception, hearing quality of life and cochlear implant functioning. These outcome measures have been defined as secondary parameters and are shown in Table 6.

Table 6. Phase 1: secondary outcome measures.

Research question	Outcome measure	Measurement method	Timing of measurement(s)
Function of the implant and the response of the auditory nerve	Telemetric data	Electrically evoked Compound Action Potential (ECAP)	During implantation and at fitting sessions in week 1, 8 and 20
Sound perception	Spatial masking	Spatial Speech Perception In Noise (SSPIN)	At week 10 and 26
	Listening effort	Listening effort scaling	At week 4, 12 and 26
	Sound quality	Sound quality questionnaire	At week 3, 12 and 26
	Spectral resolution	Spectral-temporally Modulated Ripple Test (SMRT)	At week 4, 12 and 26
	Loudness scaling	Adaptive Categorical Loudness Scaling (ACALOS)	At week 4, 12 and 26
Hearing quality of life	Hearing abilities	Speech-Spatial-Qualities of hearing scale (SSQ)	Before implantation and at week 26
	Quality of life	Health Utility Index Mark 3 (HUI-3)	Before implantation and at week 26
	Capability of self-development	ICEpop CAPability measure for Adults questionnaire (ICECAP-O)	Before implantation and at week 26

7.1.2 Phase 2: bimodal adjustment

Main study parameter/endpoints

At the end of phase 1, patients indicate their final preference for either the conventional or the experimental CI program and will be instructed to only this program for the remainder of the study. When patients choose to retain a hearing aid in the non-implanted ear, bimodal fitting will be performed in phase 2. It is thought that by matching CI and HA in a similar manner, an augmented bimodal experience can be achieved, of which the primary outcome measures are shown in Table 7. Comparisons will be made between the HA under standard clinical fitting, as achieved during phase 1, and under bimodal fitting.

Table 7. Phase 2: primary outcome measures.

Research question	Outcome measure	Measurement method	Timing of measurement(s)
Fitting preference in daily life	Patients satisfaction	Satisfaction scale	At every fitting consultation
Speech understanding	Word score in quiet	Dutch Consonant Nucleus Consonant test	See overview in section 3 (Figure 6)
	Sentence score in quiet	Dutch matrix sentence test in quiet condition	See overview in section 3 (Figure 6)
	Sentence score in noise	Dutch matrix sentence test in noise condition	See overview in section 3 (Figure 6)

Secondary study parameters/endpoints

It is hypothesized that bimodal fitting will also improve sound perception and hearing quality of life. These outcome measures have been defined as secondary parameters and are shown in Table 8.

Table 8. Phase 2: secondary outcome measures.

Research question	Outcome measure	Measurement method	Timing of measurement(s)
Sound perception	Spatial masking	Spatial speech Recognition In Noise (SPIN)	At week 30 and 34
	Listening effort	Listening effort scaling	At week 30 and 34
	Sound quality	Sound quality questionnaire	At week 34
	Loudness scaling	Adaptive Categorical Loudness Scaling (ACALOS)	At week 30 and 34
Hearing quality of life	Hearing abilities	Speech-Spatial-Qualities of hearing scale (SSQ)	At week 34
	Quality of life	Health Utility Index Mark 3 (HUI-3)	At week 34
	Capability of self-development	ICEpop CAPability measure for Adults questionnaire (ICECAP-O)	At week 34

7.1.3 Phase 3: clinical fit

Main study parameter/endpoints

At the end of phase 2, patients indicate their final preference for either the conventional or bimodal HA fitting. In combination with the preferred CI settings, as indicated at the end of phase 1, a clinical fit will be performed for both CI and HA. In phase 3, patients will change their loaner CI processor for their own Naída device and corresponding settings will be transferred with Soundwave. When patients want to retain a contralateral HA, they can keep the Naída Link (with either the

conventional or bimodal settings) or switch back to the device they used before. This clinical fit will then be compared with previous conditions when patients come back to the Maastricht UMC+ at week 52. Primary outcome measures are shown in Table 9.

Table 9. Phase 3: primary outcome measures.

Research question	Outcome measure	Measurement method	Timing of measurement(s)
Fitting preference in daily life	Patients satisfaction	Satisfaction scale	At every fitting consultation
Speech understanding	Word score in quiet	Dutch Consonant Nucleus Consonant test	See overview in section 3 (Figure 6)
	Sentence score in quiet	Dutch matrix sentence test in quiet condition	See overview in section 3 (Figure 6)
	Sentence score in noise	Dutch matrix sentence test in noise condition	See overview in section 3 (Figure 6)

<u>Secondary study parameters/endpoints</u>

Additional secondary parameters for the clinical fit are shown in Table 10.

Table 10. Phase 3: secondary outcome measures.

Research question	Outcome measure	Measurement method	Timing of measurement(s)
Sound perception	Listening effort	Listening effort scaling	At week 52
Hearing quality of life	Hearing abilities	Speech-Spatial-Qualities of hearing scale (SSQ)	At week 52
	Quality of life	Health Utility Index Mark 3 (HUI-3)	At week 52
	Capability of self-development	ICEpop CAPability measure for Adults questionnaire (ICECAP-O)	At week 52

7.2 Randomization, blinding and treatment allocation

In this study a new type of trial design will be implemented, in which patients stand as their own control and treatment allocation is based on daily randomization. In total, the intervention phase in which randomization will be applied, will last around 3 months. In this period patients will be allocated each day to either the CI program with the conventional FAT or the experimental FAT with a ratio of 1:1. This type of randomization limits possible bias of systemic sound processor use over time. This might occur when patients systematically use their CI according to a set schedule, for example when subjects switch off their processor for weekly swimming or meditation sessions. For the allocation, a randomization sequence is created for each patient using Wolfram Mathematica 11.3. Counterbalancing will be applied between-subjects.

In constructing this randomization method two specific difficulties had to be tackled. First, patients may develop a preference for any program which is given first during the rehabilitation period. Based on an analysis of clinical data within Maastricht UMC+, two distinct time periods have roughly been defined in the learning curve of CI patients (Figure 8). It seems that the first 4 weeks of CI rehabilitation are characterized by a large improvement in speech understanding. On average, patients tend to show a steep learning curve within this time window and it can therefore be hypothesized that this first period of adaptation is indeed crucial in the learning process. In terms of randomization, it is therefore important to acknowledge that allocation to either the conventional

program or the experimental program during the first 4 weeks will have a major impact on the preference for one or the other FAT. Therefore, it has been defined that within this time window patients will not be allocated more than two consecutive days to the same program. After 4 weeks, CI rehabilitation tends to show a more flattened learning curve. From this point on, randomization has been restricted to no more than four consecutive days of allocating the same program.

Figure 8. Learning curve constructed with speech understanding scores (%) of a clinical data set from Maastricht UMC+ CI patients. Testing performed at 55 dB, 65 dB and 75 dB Sound Pressure Levels.

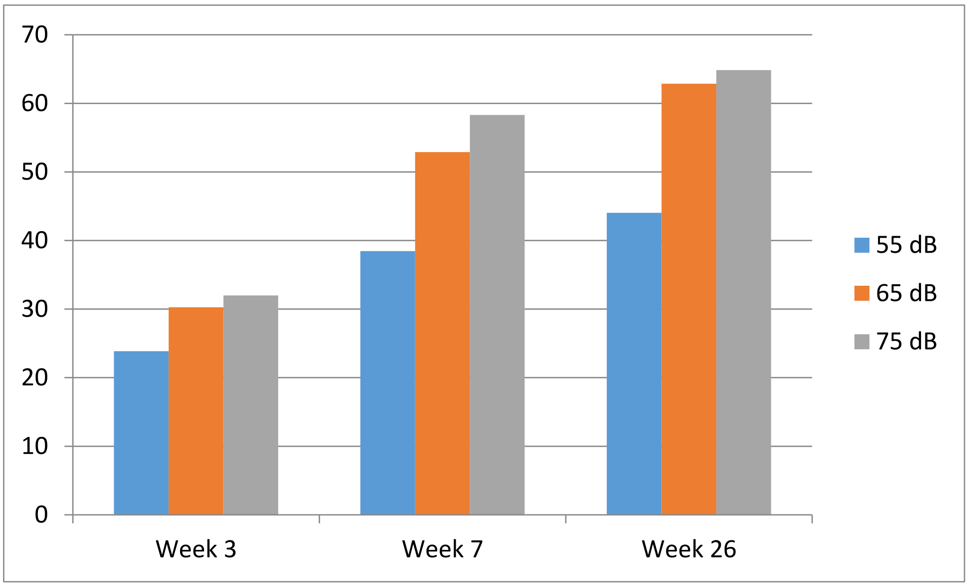

A second problem that had to be addressed in the randomization procedure, is the given that patients may start recognizing which settings corresponds to which processor channel and thereby develop a fixed preference and blinding is lost. For example, if channel 1 is programmed with the conventional FAT and channel 2 with the experimental FAT for the full rehabilitation period, then preference may be based more on prejudice than solely on daily assessment of sound quality. To overcome this problem, randomization has not only been constructed for the patient but also for the fitting clinician. This procedure determines whether processor programs, with corresponding FAT settings, will be switched or not. By applying this random chance at every fitting session during the daily randomization period patients will be shielded for developing knowledge of program assignment. This also limits the effect a non-compliance issue might have on the subject. For example, if they structurally forget to switch programs.

An example of a randomization schedule for the first 4 weeks of the rehabilitation period is shown in Figure 9. The output shows daily allocation to either using processor program 1 (red) or program 2 (blue) with a restriction of generating no more than two consecutive days with the same channel. For this test subject, the experimental FAT is programmed on channel 1 with the conventional FAT saved on channel 2 at baseline. At the 3rd and 4th fitting consultation however, FAT programs will be switched.

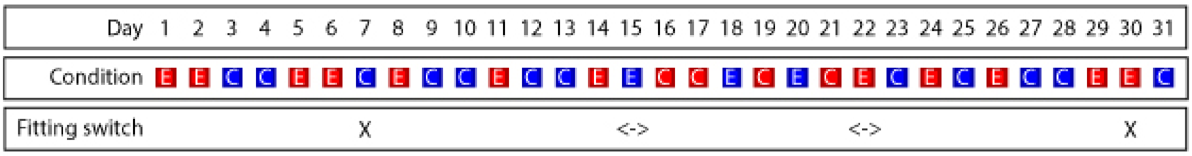

Figure 9. Example of a randomization schedule for the first 4 weeks of the study. Red box = first processor channel, blue box = second processor channel, E = experimental condition (Test), C = conventional condition (Control), X = no switch, <-> = switch.

Similar output is generated for a remainder of the rehabilitation period (Figure 10). During this time window, patients will not be allocated to use the same processor channel for more than four days in a row.

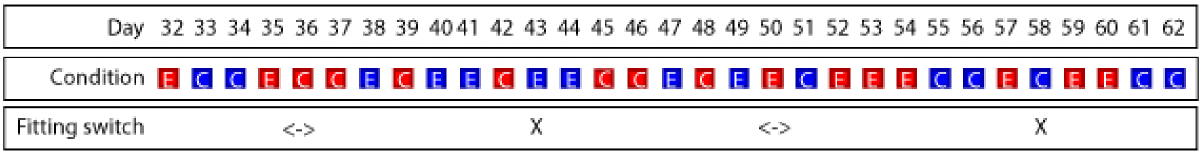

Figure 10. Example of a randomization schedule for weeks 5-12 of the study. Red box = first processor channel, blue box = second processor channel, E = experimental condition (Test), C = conventional condition (Control), X = no switch, <-> = switch.

Patients will be blinded as such that they are unaware which CI processor program will contain the experimental FAT at which point in time. Special attention will be given to this fact during the fitting procedures. Patients will not be given any visual or verbal cues that may lead to recognizing which channel contains the experimental settings. No information will be given by the clinician and computer screens that may contain information on fitting settings will be shielded to the patient. As discussed earlier, processor channels with corresponding FAT settings will also be switched regularly to prevent program assignment. Clinicians and data collectors will not be blinded as this will be practically impossible to do so.

7.3 Study procedures

7.3.1 General information

As described in section 3 (study design), the study can be classified in three different phases. During phase 1, patients will combine their CI rehabilitation with exposure to both the conventional and experimental program based on a daily randomized scheme. This time window is followed by a period of free choice in which patients have the liberty of choosing whatever program they prefer. Those subjects who retain a hearing aid in the contralateral ear will also be enrolled in phase 2, in which optimization of HA fitting will be performed. During phase 3, patients receive a clinical fit which will be based on the preferences they have obtained during the study period.

7.3.2 Imaging procedure

Patients will have a pre-operative CT and a post-operative CBCT scan. The CT scan is part of the regular clinical selection procedure. The additional CBCT scan is a quick (circa 30 seconds), comfortable and safe alternative to a CT scan and will be combined with ENT checkup, one week after implantation. CBCT has a very low radiation exposure (0.05 mSv). The radiation dose of this scan is as low as reasonably acceptable (ALARA) and lies well within the radiation limitations applicable within the Netherlands for this kind of extra radiation exposure. The study will also be submitted to the SBE (Radiation Protection Unit) for approval.

7.3.3 CI Fitting procedure

After surgical implantation, the CI sound processor has to be appropriately programmed and customized for the individual. This process of CI fitting ensures that the electrical pattern generated by the internal device in response to sound, yields an optimal auditory percept for the individual recipient. During fitting, thresholds for stimulation (T-level) as well as the most comfortable level of stimulation (M-level) will be determined as input for fitting the electrical dynamic range. Impedances between every electrode will be measured and calculated during each fitting appointment. Evoked Compound Action Potential (ECAP) on and between every electrode, will be measured at several time points, starting preoperatively as included in the standard clinical routine. Patients in this study will follow CI fitting according to normal clinical routine, with the exception that two programs with different FAT settings will be programmed instead of one. Also, BEPS+ research software will be used since the regular clinical software does not provide the full range of possibilities to adjust frequency allocation tables and activate phantom stimulation.

Phase 1 of the study starts with a period of 3 months in which patients will use the CI processor according to a daily randomization scheme, as can be seen in the study timeline in Figure 6. This scheme will be given to the patient after the first fitting session. After the randomization time window, a period of 3 months will follow in which the patient is free to use each program, any day. Testing procedures during this period will still be performed with both programs. At the end of phase 1, the patient decides whether the conventional or the experimental settings are preferred and complies to use only this selected program until the end of the study. If the patient chooses to retain a contralateral hearing aid, the HA fitting procedure as explained in 7.3.4. will be implemented.

7.3.4 HA fitting procedure

Patients will receive a Naída Link hearing aid during the first CI fitting session for the duration of the study. At this time, a normal HA fitting protocol will be performed in correspondence to clinical routine. A more extensive fitting will be done two weeks later based on Real-Ear Measurement (REM), which is also part of regular care. When study phase 1 is finished, and patients have shown to retain their bimodal hearing aid (a minimum of 50% of their total CI usage), then they will also be included for the experimental HA fitting procedure. Patients that did not use their hearing aid according to this criterium will have no intervention during phase 2 but will still follow the regular study outline. This means they will be enrolled in phase 3 after the normal time period of phase 2 has expired. In phase 2, the hearing aid is programmed based on the loudness growth experience by taking into account the dynamic range with regard to T- and M-level. This is in contrast to conventional fitting, where fitting is based on residual hearing thresholds alone. It is proposed that by tuning CI and HA in a same manner, an augmented bimodal experience can be achieved. Eight weeks later patients decide which HA program has their preference.

7.3.5 Further clinical rehabilitation

Patients will have consultations with a speech therapist and social worker, in correspondence with normal clinical routine and will be asked to practice daily at home for 1 hour, using the training material as handed out by the speech therapist. During these practice moments they are required to

wear only their CI, without contralateral hearing aid, to optimally train the new hearing ability. For the rest of the time they are allowed to wear both hearing aid and CI together.

7.3.6 Shared decision making and clinical follow-up

At the end of both phase 1 and 2, patients indicate their preference for either the conventional or the experimental settings. If desirable, the audiologist will aid the shared decision making process by sharing the patients test results. To avoid interference with decision making these results will not be shared any earlier with the patient. The preferred CI map, as created by BEPS+ during the first stage of the project, will be translated as closely as possible to the clinical software mapping in Soundwave in phase 3. A re-fitting of the contralateral hearing aid may also occur.

7.3.7 Test procedures

Audiometric testing

Audiometry
Aided (free-field) and unaided (headphones) tone audiometric thresholds will be evaluated for the electrical/acoustical hearing situations.

Dutch Consonant Nucleus Consonant (CNC) tests
Phoneme scoring (%correct) in quiet will be evaluated both aided (at 65 and 75dB SPL for the electrical/acoustical/combined hearing situations) and unaided at higher stimulation levels [70].

Dutch matrix sentence test
The Dutch Matrix sentence test [71–73], validated for repeated measures in CI-recipients, will be presented in quiet as well as in noise. First in quiet the percentage correct will be determined (test+retest) at a normal speech level (65dB SPL) in preparation for testing in noise. When testing in noise, an adaptive procedure is applied whereby the noise is fixed at a level of 65dB SPL while the speech level is varied. This results in the speech-reception-threshold (SRT), the signal-to-noise ratio at which the subject is able to still understand 50% of the sentences correctly. This will be performed for the electrical as well as the bimodal situation. When assessing the benefit of the contralateral hearing aid the noise will also be presented from spatially separated loudspeakers (±90°) in order to determine the head shadow and squelch effect [74]. This will be done with the Spatial Speech Perception In Noise (SSPIN) test.

Loudness scaling test
To estimate the course of loudness percept between minimal audible level and maximum comfortable level, a procedure is used that automatically adjusts the presentation levels to the subject's individual auditory dynamic range without employing any pre-measurement. The procedure uses repeated measurements and presents levels in randomized order. It has been named "ACALOS" (Adaptive Categorical LOudness Scaling) [75]. Results will provide information about the difference in loudness percept between CI and HA and will give input for optimizing the fitting of these hearing devices across the dynamic range.

Listening effort test
In a listening effort test subjects are asked to rate the effort it takes to listen to speech fragments in noise. For the rating a visual analogue scale (VAS) is used [76] and speech and noise (Dutch Matrix

Test) are presented from the same loudspeaker in front of the subject. Three relevant signal-to noise ratios will be tested repeatedly (5 times) and the mean effort level will be calculated.

Frequency selectivity
The ability to spectrally resolve frequency information is known to be related to speech understanding performance (in noise) [77,78]. Frequency selectivity will be monitored to test the ability of the patient to filter out one stimulus from the others on the basis of frequency. This will be done with the Spectral-temporally Modulated Ripple Test (SMRT).

<u>CI data measurements</u>

ECAP
The electrically Evoked Compound Action Potential (ECAP) reflects the response of auditory nerve fibers to stimulation of CI electrodes. In CI's made by Advanced Bionics, it is possible to measure ECAPs with a technique called Neural Response Imaging (NRI) [79]. It can be used to check electrode integrity and the ability of the neuron to give a response when stimulated. If the NRI response is substantially different on one electrode compared to another, there may be a problem with the electrode (most likely) or the neuron (less common). Action is also needed if no ECAP is recorded at all. We believe that these measurements (and their evolution over time) can add extra information to the electrode position estimations in relation to intracochlear structures.

<u>Questionnaires</u>

Patients satisfaction
At every fitting session, patients will be asked to rate their satisfaction with either the conventional or experimental program on a 10-point VAS scale.

Sound quality questionnaire
The perceived sound quality delivered by the different mappings of the CI and HA will be assessed by a sound quality questionnaire [41] based on the sound quality attributes by Boretzki [80]. Previously, Devocht et al. [41] translated the initial set of 21 descriptors by Boretzki to a Dutch set of 10 features for a study with bimodal CI users. The questionnaire asks patients to describe 'how a familiar speaker in quiet conditions sounds' by rating the set of 10 on a linear rule from 0 (not at all) to 10 (very). The set of predictors consists of the features voluminous, dull or damped, sharp, bright or harsh, tinny or metallic, shrill, hard, nasal, unclear or blurry and unpleasant.

Speech-Spatial-Qualities of hearing scale (SSQ)
The subjective hearing ability in daily life will be assessed by the short form of the Speech-Spatial-Qualities (SSQ) hearing scale [81,82]. It is designed to measure hearing disabilities across certain domains and intents to question hearing impairment and how those disabilities determine the experience of handicap. In total, it comprises 12 scored items which are rated on a scale from 0 (not at all) to 10 (perfectly).

Health Utility Index Mark 3 (HUI-3)
To generally assess the health related quality of life the Health Utility Index Mark 3 (HUI-3) will be used [83]. It is designed to measure general health status and is comprised of 8 attributes, namely vision, hearing, speech, ambulation, dexterity, emotion, cognition and pain.

ICEpop CAPability measure for Adults questionnaire (ICECAP-O)
The ICEpop CAPability measure for adults (ICECAP-A) [84] will be applied to measure to which extent patients are able to lead their lives the way they want to. It is focused more on wellbeing defined in a broader sense, rather than health. It comprises of the attributes attachment, security, role, enjoyment and control. At each attribute, subjects are asked to tick a box of which statement best describes their situation.

7.4 Withdrawal of individual subjects

Subjects can leave the study at any time for any reason if they wish to do so without any consequences. The investigator can decide to withdraw a subject from the study for urgent medical reasons. Also, subjects will specifically be removed from the study population if they do not comply with randomization procedures. A smooth transition to general clinical and audiological care will be ensured for every individual who will be withdrawn from the study. If applicable, specific attention will be given to properly addressing open adverse events (see section 8.4).

7.5 Replacement of individual subjects after withdrawal

Subjects will not be replaced after withdrawal.

7.6 Follow-up of subjects withdrawn from treatment

Patients that withdraw from the study will receive follow-up according to the standard CI-care program of CI-team South-East Netherlands.

7.7 Premature termination of the study

If premature termination of the study occurs, patients will directly enter phase 3 thereby receiving follow-up according to the standard CI-care program of CI-team South-East Netherlands. Participation, withdrawal, replacement, or premature termination of this study will not in any way affect the clinical care a patient receives from their CI-team.

8. SAFETY REPORTING

8.1 Temporary halt for reasons of subject safety

In accordance to section 10, subsection 4, of the WMO, the sponsor will suspend the study if there is sufficient ground that continuation of the study will jeopardise subject health or safety. The sponsor will notify the accredited METC without undue delay of a temporary halt including the reason for such an action. The study will be suspended pending a further positive decision by the accredited METC. The investigator will take care that all subjects are kept informed.

8.2 AEs, SAEs and SUSARs

8.2.1 Adverse events (AEs)

Adverse events are defined as any undesirable experience occurring to a subject during the study, whether or not considered related to [the investigational product/trial procedure/the experimental intervention]. All adverse events reported spontaneously by the subject or observed by the investigator or his staff will be recorded.

8.2.2 Serious adverse events (SAEs)

A serious adverse event is any untoward medical occurrence or effect that

- Results in death;
- Is life threatening (at the time of the event);
- Requires hospitalisation or prolongation of existing inpatients' hospitalization;
- Results in persistent or significant disability or incapacity;
- Is a congenital anomaly or birth defect; or
- Any other important medical event that did not result in any of the outcomes listed above due to medical or surgical intervention but could have been based upon appropriate judgement by the investigator.

An elective hospital admission will not be considered as a serious adverse event.

All SAEs will be reported as described by the CCMO SAE flow for medical research [85]. The study team will report the SAEs through the web portal ToetsingOnline to METC azM/UM with line listings. These are overview lists of SAEs which can be filed periodically, as described in WMO article 10, paragraph 2. Line listings will be reported before the end of each calendar year during the study period. All types of SAEs will be included in the lists.

8.2.3 Suspected unexpected serious adverse reactions (SUSARs)

Since the study does not involve medicinal products, this section is considered to be non-applicable in the case of the current study.

8.3 Annual safety report

Since the study does not involve medicinal products, this section is considered to be non-applicable in the case of the current study.

8.4 Follow-up of adverse events

All AEs will be followed until they have abated, or until a stable situation has been reached. Depending on the event, follow up may require additional tests or medical procedures as indicated, and/or referral to the general physician or a medical specialist.

SAEs need to be reported till the end of the study, as defined in the protocol.

8.5 [Data Safety Monitoring Board (DSMB) / Safety Committee]

The need for the set-up of a DSMB has been assessed and is considered not to add to the study. To come to this decision the EMEA guidelines on Data Monitoring committees were consulted (*http://www.emea.europa.eu*) and aspects such as indication, study intervention, study endpoints as well as study population were taken into consideration:

- By no means there is a life threatening disease or situation involved. The patients under investigation are severely hearing impaired patients and the main surgical intervention, cochlear implantation, will be performed as standard clinical care.
- The study does not involve under aged children or incompetent or mentally disabled patients. The study population consists of adult (>18 years old) hearing impaired patients who already are under the care of the CI-team of the Maastricht UMC+.
- There is no prior knowledge or strong suspicion that the intervention of the study has the potential to harm patients.
- The study design does not give reason for setting up a DSMB.

9. STATISTICAL ANALYSIS

In this study a new type of trial design will be implemented, in which patients stand as their own control and treatment allocation is based on daily randomization. Study parameters have been discussed in section 7.1 and will be reported in a quantitative manner. The outcomes for the different test conditions under investigation (see section 7.3) will descriptively be presented by stating mean, standard deviation, median/interquartile range and minimum/maximum values. Normality will be assessed by examining histograms and performing the Kolmogorov-Smirnov test. Depending on whether normality can be established, either nonparametric or parametric statistical tests will be performed.

9.1 Primary study parameter(s)

Objective outcome

Learning curves of speech understanding will be established by measuring the Area Under the Curve (AUC).

Subjective outcome

Patients fitting preference for either the conventional or the experimental program will be compared with a Chi-square test.

9.2 Secondary study parameter(s)

Statistical analysis for secondary parameters will be further evaluated in a separate statistical analyses plan which will be created before the database lock.

9.3 Interim analysis (if applicable)

An interim analysis will be performed on the stated preference of subjects when half of the subjects have indicated their preference (6 month interval). This analyses will be done to allow the manufacturer to decide on whether or not to start the early development of implementing the new procedures in standard clinical practice. This might benefit subjects who would prefer the test intervention to use it in daily life situations also after the end of the study.

10. ETHICAL CONSIDERATIONS

10.1 Regulation statement

The study will be conducted according to the principles of the Declaration of Helsinki, seventh revision (2013), and in accordance with the Medical Research Involving Human Subjects Act (WMO).

10.2 Recruitment and consent

Patients will be recruited from the existing CI patient cohort of the CI-team South-East Netherlands. Beforehand, these patients have already underwent a thorough screening procedure at Maastricht University Medical Center+ in which they were evaluated by a multidisciplinary audiological and medical team qualified for receiving a CI. During and after this procedure eligible patients will already be informed by the involved audiologist or screening coordinator about the possibility of participating in the study. If patients show interest and agree to receive further information, an information letter accompanied by a short informed consent form will be given directly or send by mail. A short introduction will also be given at a plenary session which is usually hosted by involved social workers and speech therapists. This will be done in a strictly informative way, thereby not coaching or persuading patients to take part in the study.

Subjects will be given time to consider their participation. When a patient is willing to participate in the study he or she can indicate this during the CI selection procedure or respond by e-mail to the researcher. The researcher will then make an appointment with the subject and have an oral discussion to be sure the study information is adequately understood. The researcher is also responsible to fill out the informed consent together with the subject according to CCMO guidelines. If there are still questions or unsure considerations, it will also be possible for the potential subjects to contact the researcher or if desired, the independent physician, the ENT-physician/surgeon or the involved audiologist. The informed consent will be signed at the latest one week before the operation (surgical placement of the CI) is scheduled. When the patient indicates he or she is not willing to participate in the study, or no response is received after receiving the information letter, the patient will be excluded as a possible participant.

When the anticipated goal of 30 participants completing the randomization period of the study is reached, no further inclusion will be conducted. If the minimum sample size of 20 participants is not reached within the inclusion period, recruitment may be extended.

10.3 Objection by minors or incapacitated subjects (if applicable)

This section is considered to be not applicable in this study since inclusion criteria ask for adult (>18 years of age) and capacitated subjects.

10.4 Benefits and risks assessment, group relatedness

The aim of the study is to improve the outcomes of electrical hearing through individual fitting based on natural hearing alignment. One may expect that based on these results the clinical practice of CI patients can be improved, both on an individual level and on a group basis. There are no substantial health risks specifically associated with study participation. There is one additional cone beam CT scan associated with a low radiation dosage. CE-marked hearing equipment (CI and HA) is used within the scope of standard care. Participation however takes time, effort and attention from

subjects. Specifically, patients will train two CI fitting programs over one in standard clinical care. This may either be a disadvantage or a benefit. In case it would be a disadvantage, the risk and burden would be limited to 6 months, after which the effects of the double fitting can be expected to wash out. Patients have to come to the hospital for extra test sessions. As a result of the study, subjects may or may not choose the continued use of the new fitting strategy of their hearing equipment to improve their performances as far as the standard clinical sound processors support it. In the case of promising results, manufacturer Advanced Bionics may apply the concept of natural fitting to clinical use thereby improving patient-related care in CI rehabilitation. Recruitment will be performed in a standard pool of CI-patients, thereby making study results directly applicable to clinical care.

10.5 Compensation for injury

The sponsor/investigator has a liability insurance which is in accordance with article 7 of the WMO.

The sponsor (also) has an insurance which is in accordance with the legal requirements in the Netherlands (Article 7 WMO). This insurance provides cover for damage to research subjects through injury or death caused by the study. The insurance applies to the damage that becomes apparent during the study or within 4 years after the end of the study.

10.6 Incentives (if applicable)

For each visit to the MUMC+ related to study participation that is not combined with regular clinical routine travelling expenses will be compensated for (0.19 euro per km or public transport).

11. ADMINISTRATIVE ASPECTS, MONITORING AND PUBLICATION

11.1 Handling and storage of data and documents

Data will be encrypted with a code (see also section F4 of the ABR-form) and stored. The key to the code is safeguarded in a file which is accessible for the researchers who participate in this project. Other persons that will get access to study data are a monitor and representatives from the national 'Inspectie voor Gezondheidszorg en Jeugd'. Data will not be stored longer than necessary. The Maastricht University Medical Center+ maintains strict requirements for ensuring the privacy of patients. Study results will be stored for 15 years after study termination, which is in agreement with the "General Data Protection Regulation (GDPR)" and the "International Converence on Harmonisation (ICH)/WMO Good Clinical practice (ICH GCP)".

11.2 Monitoring and Quality Assurance

A monitoring plan will be composed in agreement with Clinical Trial Center Maastricht.

11.3 Amendments

Amendments are changes made to the research after a favourable opinion by the accredited METC has been given. All amendments will be notified to the METC that gave a favourable opinion.

A 'substantial amendment' is defined as an amendment to the terms of the METC application, or to the protocol or any other supporting documentation, that is likely to affect to a significant degree:
the safety or physical or mental integrity of the subjects of the trial;
the scientific value of the trial;
the conduct or management of the trial; or
the quality or safety of any intervention used in the trial.

All amendments, both substantial and non-substantial, will be notified to the METC and to the competent authority.

11.4 Annual progress report

The sponsor/investigator will submit a summary of the progress of the trial to the accredited METC once a year. Information will be provided on the date of inclusion of the first subject, numbers of subjects included and numbers of subjects that have completed the trial, serious adverse events/ serious adverse reactions, other problems, and amendments.

11.5 Temporary halt and (prematurely) end of study report

The investigator/sponsor will notify the accredited METC of the end of the study within a period of 8 weeks. The end of the study is defined as the patient's last visit.

The sponsor will notify the METC immediately of a temporary halt of the study, including the reason of such an action.

In case the study is ended prematurely, the sponsor will notify the accredited METC within 15 days, including the reasons for the premature termination.

Within one year after the end of the study, the investigator/sponsor will submit a final study report

with the results of the study, including any publications/abstracts of the study, to the accredited METC.

11.6 Public disclosure and publication policy

Arrangements concerning public disposure and publication of research data have been made in arrangement with the CCMO statement on publication policy [86]. The study will be registered in a public trial registry before the first patient is recruited. For further information concerning the agreement between sponsor and financier, the contract between both parties can be consulted in section K3 of the research dossier.

12. STRUCTURED RISK ANALYSIS

12.1 Potential issues of concern - synthesis

There are no known health risks associated with participation in this study. Only the software used for CI fitting does not have a CE-marking. The risk of using BEPS+ however is considered to be non-significant as demonstrated in the risk-analysis of the IMDD as part of section D of the research dossier. No intolerable or undesirable risks are associated with this study and the risk does not differ from the risk in normal clinical routine. However, participation takes time and effort. Also, there may arise an undesirable situation after the study when patients prefer the experimental CI condition. Since this condition is programmed with non-clinical software, it has to be imitated with regular clinical software when the study is finished. Since this software does not have the same functionalities, there is a chance patients will be confronted with a slight decrease in hearing performance if the intervention was successful.

13. REFERENCES

1. OPCI. Aantal implantaties in Nederland [Internet]. 2016 [cited 4 Jul 2018]. Available: https://www.opciweb.nl/ci-centra/ci-centra-in-nederland/aantal-implantaties-in-nederland/).
2. Gifford RH, Dorman MF, Shallop JK, Sydlowski SA. Evidence for the expansion of adult cochlear implant candidacy. Ear Hear. 2010;31: 186–194. doi:10.1097/AUD.0b013e3181c6b831
3. Hughes MLM, Neff DDL, Simmons JL, Moeller MP. Performance Outcomes for Borderline Cochlear Implant Recipients With Substantial Preoperative Residual Hearing. Otol Neurotol. 2014;35: 1373–1384. Available: http://www.ncbi.nlm.nih.gov/pubmed/24662626
4. Turner C. Integration of acoustic and electrical hearing. J Rehabil Res Dev. 2008;45: 769–778. doi:10.1682/JRRD.2007.05.0065
5. Ching T, van Wanrooy E, Dillon H. Binaural-bimodal fitting or bilateral implantation for managing severe to profound deafness: a review. Trends Amplif. 2007;11: 161–192. Available: http://www.ncbi.nlm.nih.gov/pubmed/17709573
6. Schafer EC, Amlani AM, Paiva D, Nozari L, Verret S. A meta-analysis to compare speech recognition in noise with bilateral cochlear implants and bimodal stimulation. Int J Audiol. 2011;50: 871–880. Available: http://www.ncbi.nlm.nih.gov/pubmed/22103439
7. Incerti P V., Ching TYC, Cowan R. A Systematic Review of Electric-Acoustic Stimulation: Device Fitting Ranges, Outcomes, and Clinical Fitting Practices. Trends Amplif. 2013;17: 3–26. doi:10.1177/1084713813480857
8. Litovsky R. Development of the Auditory System. Handb Clin Neurol. 2015. pp. 55–72. doi:10.1016/S0166-2236(98)01239-9
9. Peretz I, Ayotte J, Zatorre RJ, Mehler J, Ahad P, Penhune VB, et al. Congenital Amusia: A disorder of fine-grained pitch discrimination. Neuron. 2002;33: 185–191. doi:10.1016/S0896-6273(01)00580-3
10. Zhou N, Xu L, Lee C-Y. The effects of frequency-place shift on consonant confusion in cochlear implant simulations. J Acoust Soc Am. 2010;128: 401–409. doi:10.1121/1.3436558
11. Siciliano CM, Faulkner A, Rosen S, Mair K. Resistance to learning binaurally mismatched frequency-to-place maps: implications for bilateral stimulation with cochlear implants. J Acoust Soc Am. 2010;127: 1645–1660. doi:10.1121/1.3293002
12. Goupell MJ, Stoelb C, Kan A, Litovsky RY. Effect of mismatched place-of-stimulation on the salience of binaural cues in conditions that simulate bilateral cochlear-implant listening. J Acoust Soc Am. 2013;133: 2272–2287.
13. Fitzgerald MB, Sagi E, Morbiwala T a, Tan C-T, Svirsky M a. Feasibility of real-time selection of frequency tables in an acoustic simulation of a cochlear implant. Ear Hear. 2013;34: 763–772. doi:10.1097/AUD.0b013e3182967534
14. Guérit F, Santurette S, Chalupper J, Dau T. Investigating Interaural Frequency-Place Mismatches via Bimodal Vowel Integration. Trends Hear. 2014;18: 1–10. doi:10.1177/2331216514560590
15. Reiss L a J, Ito R a, Eggleston JL, Liao S, Becker JJ, Lakin CE, et al. Pitch Adaptation Patterns in Bimodal Cochlear Implant Users: Over Time and After Experience. Ear Hear. 2014; 1–12. doi:10.1097/AUD.0000000000000114
16. Reiss L a J, Turner CW, Karsten S a., Gantz BJ. Plasticity in human pitch perception induced by tonotopically mismatched electro-acoustic stimulation. Neuroscience. IBRO; 2014;256: 43–52. doi:10.1016/j.neuroscience.2013.10.024

17. Vermeire K, Schatzer R, Punte a K, Van de Heyning P, Voormolen M, Visser D, et al. A frequency-place map for electrical stimulation in cochlear implants: Change over time. Hear Res. Elsevier B.V; 2015; doi:10.1016/j.heares.2015.03.011
18. Oxenham AJ, Bernstein JGW, Penagos H. Correct tonotopic representation is necessary for complex pitch perception. Proc Natl Acad Sci U S A. 2004;101: 1421–1425. doi:10.1073/pnas.0306958101
19. Greenwood DD. A cochlear frequency-position function for several species-29 years later. J Acoust Soc Am. 1990;87: 2592–2605.
20. Stakhovskaya O, Sridhar D, Bonham BH, Leake PA. Frequency Map for the Human Cochlear Spiral Ganglion: Implications for Cochlear Implants. J Assoc Res Otolaryngol. 2007;8: 220–233. doi:10.1007/s10162-007-0076-9
21. Landsberger DM, Svrakic M, Roland JT, Svirsky M. The Relationship Between Insertion Angles , Default Frequency Allocations , and Spiral Ganglion Place Pitch in Cochlear Implants. Ear Hear. 2015;36: e207-13.
22. Xu J, Xu S, Cohen L, Clark G. Cochlear view: postoperative radiography for cochlear implantation. Otol Neurotol. 2000;21: 49–56. Available: http://journals.lww.com/otology-neurotology/Abstract/2000/01000/Cochlear_View__Postoperative_Radiography_for.11.aspx
23. Escudé B, James C, Deguine O, Cochard N, Eter E, Fraysse B. The size of the cochlea and predictions of insertion depth angles for cochlear implant electrodes. Audiol Neurotol. 2006;11: 27–33. doi:10.1159/000095611
24. Finley CC, Holden T a, Holden LK, Whiting BR, Chole R a, Neely GJ, et al. Role of electrode placement as a contributor to variability in cochlear implant outcomes. Otol Neurotol. 2008;29: 920–8. doi:10.1097/MAO.0b013e318184f492
25. Macherey O, Deeks JM, Carlyon RP. Extending the Limits of Place and Temporal Pitch Perception in Cochlear Implant Users. J Assoc Res Otolaryngol. 2011;12: 233–251. doi:10.1007/s10162-010-0248-x
26. Saoji A a, Litvak LM. Use of “phantom electrode” technique to extend the range of pitches available through a cochlear implant. Ear Hear. 2010;31: 693–701. doi:10.1097/AUD.0b013e31820407f7
27. Nogueira W, Litvak LM, Saoji A a., Büchner A. Design and Evaluation of a Cochlear Implant Strategy Based on a “Phantom” Channel. PLoS One. 2015;10: e0120148. doi:10.1371/journal.pone.0120148
28. Boëx C, Baud L, Cosendai G, Sigrist A, Kós M-I, Pelizzone M. Acoustic to electric pitch comparisons in cochlear implant subjects with residual hearing. J Assoc Res Otolaryngol. 2006;7: 110–124. doi:10.1007/s10162-005-0027-2
29. Dorman MF, Spahr T, Gifford R, Loiselle L, McKarns S, Holden T, et al. An Electric Frequency-to-place Map for a Cochlear Implant Patient with Hearing in the Nonimplanted Ear. J Assoc Res Otolaryngol. 2007;8: 234–240. doi:10.1007/s10162-007-0071-1
30. Baumann U, Nobbe A. The cochlear implant electrode – pitch function. Hear Res. 2006;213: 34–42. doi:10.1016/j.heares.2005.12.010
31. Schatzer R, Vermeire K, Visser D, Krenmayr A. Electric-acoustic pitch comparisons in single-sided-deaf cochlear implant users: Frequency-place functions and rate pitch. Hear Res. 2014;309: 26–35. Available: http://www.sciencedirect.com/science/article/pii/S0378595513002761
32. Kalkman RK, Briaire JJ, Dekker DMT, Frijns JHM. Place pitch versus electrode location in a realistic computational model of the implanted human cochlea. Hear Res. Elsevier B.V;

2014;315: 10–24. doi:10.1016/j.heares.2014.06.003

33. Carlyon RP, Deeks JM, McKay CM. The upper limit of temporal pitch for cochlear-implant listeners: Stimulus duration, conditioner pulses, and the number of electrodes stimulated. J Acoust Soc Am. 2010;127: 1469–1478.
34. Carlyon RP, Lynch C, Deeks JM. Effect of stimulus level and place of stimulation on temporal pitch perception by cochlear implant users. J Acoust Soc Am. 2010;127: 2997–3008. doi:10.1121/1.3372711
35. Green T, Faulkner A, Rosen S. Frequency selectivity of contralateral residual acoustic hearing in bimodal cochlear implant users, and limitations on the ability to match the pitch of electric and acoustic stimuli. Int J Audiol. 2011/12/29. 2012;51: 389–398. doi:10.3109/14992027.2011.642010
36. Vermeire K, Nobbe A, Schleich P, Nopp P, Voormolen MH, Van de Heyning PH. Neural tonotopy in cochlear implants: An evaluation in unilateral cochlear implant patients with unilateral deafness and tinnitus. Hear Res. Elsevier B.V.; 2008;245: 98–106. doi:10.1016/j.heares.2008.09.003
37. Frijns JH, Briaire JJ, Grote JJ. The importance of human cochlear anatomy for the results of modiolus-hugging multichannel cochlear implants. Otol Neurotol. 2001;22: 340–349. doi:10.1097/00129492-200105000-00012
38. Carlyon RP, Macherey O, Frijns JHM, Axon PR, Kalkman RK, Boyle P, et al. Pitch Comparisons between Electrical Stimulation of a Cochlear Implant and Acoustic Stimuli Presented to a Normal-hearing Contralateral Ear. J Assoc Res Otolaryngol. 2010;11: 625–640. doi:10.1007/s10162-010-0222-7
39. Noble J, Labadie R, Gifford R, Dawant B. Image-guidance enables new methods for customizing cochlear implant stimulation strategies. IEEE Trans neural Syst Rehabil Eng. 2013;21: 820–829. Available: http://ieeexplore.ieee.org/xpls/abs_all.jsp?arnumber=6482251
40. Devocht EMJ, George ELJ, Janssen AML, Stokroos RJ. Bimodal Hearing Aid Retention after Unilateral Cochlear Implantation. Audiol Neurotol. 2015;20: 383–393. doi:10.1159/000439344
41. Devocht EMJ, Janssen AML, Chalupper J, Stokroos RJ, George ELJ. The Benefits of Bimodal Aiding on Extended Dimensions of Speech Perception : Intelligibility , Listening Effort, and Sound Quality. Trends Hear. 2017;20. doi:10.1177/2331216517727900
42. Devocht E, Dees G, Arts R, Smits J, George E, van Hoof M, et al. Revisiting place-pitch match in CI listeners using 3D imaging analysis. Ann Otol Rhinol Laryngol. 2015;submitted.
43. Gaylor JM, Raman G, Chung M, Lee J, Rao M, Lau J, et al. Cochlear Implantation in Adults. JAMA Otolaryngol - Head Neck Surg. 2013;139: 265–272. doi:10.1001/jamaoto.2013.1744
44. Ah-See KW, Molony NC, Maran AGD. Trends in randomized controlled trials in ENT: a 30-year review. J Laryngol Otol. Cambridge University Press; 1997;111: 611–613.
45. van Schoonhoven J, Sparreboom M, van Zanten BGA, Scholten RJPM, Mylanus EAM, Dreschler WA, et al. The Effectiveness of Bilateral Cochlear Implants for Severe-to-Profound Deafness in Adults : A Systematic Review. Otol Neurotol. 2013;34: 190–8. doi:10.1097/MAO.0b013e3181e3d62c
46. Sparreboom M, van Schoonhoven J, van Zanten BGA, Scholten RJPM, Mylanus EAM, Grolman W, et al. The effectiveness of bilateral cochlear implants for severe-to-profound deafness in children: a systematic review. Otol Neurotol. LWW; 2010;31: 1062–1071.
47. Zeng F-G. Challenges in improving cochlear implant performance and accessibility. IEEE Trans Biomed Eng. IEEE; 2017;64: 1662–1664.

48. Bredberg G. Cellular Pattern and Nerve Supply of the Human Organ of Corti. Acta Otolaryngol. 1968;Suppl 236: 1–138. Available: http://scholar.google.com/scholar?hl=en&btnG=Search&q=intitle:Cellular+pattern+and+nerve+supply+of+the+human+organ+of+Corti#3

49. Kawano A, Seldon HL, Clark GM, Lee Seldon H, Clark GM. Computer-aided three-dimensional reconstruction in human cochlear maps: measurement of the lengths of organ of Corti, outer wall, inner wall, and Rosenthal's canal. Ann Otol Rhinol Laryngol. 1996;105: 701–9. Available: http://www.ncbi.nlm.nih.gov/pubmed/8800056

50. Alexiades G, Dhanasingh A, Jolly C. Method to Estimate the Complete and Two-Turn Cochlear Duct Length. Otol Neurotol. 2015;36: 904–907.

51. Brendel M, Buechner A, Krueger B, Frohne-Buechner C, Lenarz T. Evaluation of the Harmony soundprocessor in combination with the speech coding strategy HiRes 120. Otol Neurotol. 2008;29: 199–202. doi:10.1097/mao.0b013e31816335c6

52. Ambrosch P, Müller-Deile J, Aschendorff A, Laszig R, Boermans PP, Frijns J, et al. European Adult Multi-Centre HiRes ® 120 Study — An Update on 65 Subjects. Cochlear Implants Int. 2010;11: 406–411. doi:10.1179/146701010X12671177204183

53. Saoji A, Litvak L, Boyle P. SPAN: Improved Current Steering on the Advanced Bionics CII and HiRes90K System. Cochlear Implants Int. 2010;11: 465–468. doi:10.1179/146701010X12671177204381

54. Robinson K, Summerfield a Q. Adult auditory learning and training. Ear and hearing. 1996. p. 51S–65S. doi:10.1097/00003446-199617031-00006

55. Tyler RS, Summerfield a Q. Cochlear implantation: relationships with research on auditory deprivation and acclimatization. Ear and hearing. 1996. p. 38S–50S. doi:10.1097/00003446-199617031-00005

56. Grasmeder ML, Verschuur CA, Batty VB. Optimizing frequency-to-electrode allocation for individual cochlear implant users. J Acoust Soc Am. 2014;136: 3313–3324. doi:10.1121/1.4900831

57. Svirsky M a., Talavage TM, Sinha S, Neuburger H, Azadpour M. Gradual adaptation to auditory frequency mismatch. Hear Res. Elsevier B.V.; 2015;322: 163–170. doi:10.1016/j.heares.2014.10.008

58. Nogaki G, Fu Q-J, Galvin JJ. Effect of training rate on recognition of spectrally shifted speech. Ear Hear. 2007;28: 132–140. doi:10.1097/AUD.0b013e3180312669

59. Li T, Galvin JJ, Fu Q-J. Interactions between unsupervised learning and the degree of spectral mismatch on short-term perceptual adaptation to spectrally shifted speech. Ear Hear. 2009;30: 238–249. doi:10.1097/AUD.0b013e31819769ac

60. Devocht EM, Stokroos RJ, Janssen AML, Chalupper J, George EL. The Benefits of Bimodality on Extended Dimensions of Speech Perception: Intelligibility, Effort and Quality. 2016;

61. Scherf FW a C, Arnold LP. Exploring the clinical approach to the bimodal fitting of hearing aids and cochlear implants: results of an international survey. Acta Otolaryngol. 2014;134: 1151–1157. doi:10.3109/00016489.2014.914244

62. Yehudai N, Shpak T, Most T, Luntz M. Functional Status of Hearing Aids in Bilateral-Bimodal Users. Otol Neurotol. 2013;Epub. doi:10.1097/MAO.0b013e3182898131

63. Thiese MS. Observational and interventional study design types; an overview. Biochem medica Biochem medica. Medicinska naklada; 2014;24: 199–210.

64. Fleiss JL. Statistical methods for rates and proportions. 2nd ed. New York, NY: John Wiley and Sons; 1981.

65. Cohen J. Quantitative methods in psychology: A power primer. Psychol Bull. 1992;112: 155–159.
66. Lerman J. Statistics Study design in clinical research : sample size estimation and power analysis. Canedian J Anesth. 1996;43: 184–191.
67. Rosner B. Estimation of sample size and power for comparing two binomial proportions. Fundam Biostat 7th ed Bost Brooks/Cole. 2011; 381–390.
68. Berger MPF, Imbos TJ, Janssen MPE. Methodologie en statistiek 2. Universitaire pers Maastricht; 2000.
69. Dees G, Smits JJ, Janssen AML, Hof JR, Gazibegovic D, Hoof M van, et al. A Mid-scala Cochlear Implant Electrode Design Achieves a Stable Post-surgical Position in the Cochlea of Patients Over Time—A Prospective Observational Study. Otol Neurotol. Wolters Kluwer; 2018;39: e231–e239.
70. Bosman AJ. Speech perception by the hearing impaired. Rijksuniversiteit Utrecht; 1989.
71. Houben R, Koopman J, Luts H, Wagener KC, van Wieringen A, Verschuure H, et al. Development of a Dutch matrix sentence test to assess speech intelligibility in noise. Int J Audiol. 2014;53: 760–3. doi:10.3109/14992027.2014.920111
72. Theelen-van den Hoek FL, Houben R, Dreschler WA. Investigation into the applicability and optimization of the Dutch matrix sentence test for use with cochlear implant users. Int J Audiol. 2014;53: 817–28. doi:10.3109/14992027.2014.922223
73. Houben R, Dreschler WA. Optimization of the Dutch Matrix Test by Random Selection of Sentences From a Preselected Subset. Trends Hear. 2015;19: 1–10. doi:10.1177/2331216515583138
74. Dillon H. Hearing Aids, Second Edition [Internet]. Thieme Medical Publishers; 2012. Available: https://books.google.nl/books?id=e9l%5C_sW3TOz8C
75. Brand T, Hohmann V. An adaptive procedure for categorical loudness scaling. J Acoust Soc Am. 2002;112: 1597–1604.
76. Schulte M, Wagener K, Vormann M, Dillier N, Büchner M. D-7-4: Report Tests for listening effort. In: Shulte M, editor. Hearing in the Communiction Society. Public rep. 2008. pp. 1–24.
77. Anderson ES, Nelson D a, Kreft H, Nelson PB, Oxenham AJ. Comparing spatial tuning curves, spectral ripple resolution, and speech perception in cochlear implant users. J Acoust Soc Am. 2011;130: 364–75.
78. Zhang T, Spahr AJ, Dorman MF, Saoji A. Relationship between auditory function of nonimplanted ears and bimodal benefit. Ear Hear. 2013;34: 133–141. doi:10.1097/AUD.0b013e31826709af
79. Koch DB, Overstreet EH. Neural Response Imaging: Measuring Auditory-Nerve Responses from the Cochlea with the HiResolution™ Bionic Ear System. 2014.
80. Boretzki M. Quantification of significant sound quality attributes in the context of hearing instrument fine tuning. Phonak Hear Syst Focus #25. 1999; 3–11. Available: http://www.phonakpro.com/content/dam/phonak/b2b/C_M_tools/Library/focus/en/028_0603_02_focus_25.pdf
81. Gatehouse S, Noble W. The Speech , Spatial and Qualities of Hearing Scale (SSQ). Int J Audiol. 2004;43: 85–99. Available: http://www.ncbi.nlm.nih.gov/pubmed/15035561
82. Noble W, Jensen NS, Naylor G, Bhullar N, Akeroyd M a. A short form of the Speech, Spatial and Qualities of Hearing scale suitable for clinical use: the SSQ12. Int J Audiol. 2013;52: 409–12. doi:10.3109/14992027.2013.781278

83. Furlong WJ, Feeny DH, Torrance GW, Barr RD. The Health Utilities Index (HUI) system for assessing health-related quality of life in clinical studies. Ann Med. 2001;33: 375–84. Available: http://www.ncbi.nlm.nih.gov/pubmed/11491197

84. Al-Janabi H, Flynn TN, Coast J. Development of a self-report measure of capability wellbeing for adults: The ICECAP-A. Qual Life Res. 2012;21: 167–176. doi:10.1007/s11136-011-9927-2

85. CCMO. Ongewenste voorvallen flow WMO-plichtig onderzoek Onderzoek met medische hulpmiddelen [Internet]. 2016. Available: http://www.ccmo.nl/attachments/files/flow-ongewenste-voorvallen-versie12-onderzoek-met-medische-hulpmiddelen.pdf

86. CCMO statement on publication policy [Internet]. 2002. Available: http://www.ccmo.nl/attachments/files/ccmo-statement-publicatiebeleid-3-02-en.pdf

87. van den Boogert T, van Hoof M, Handschuh S, Glueckert R, Guinand N, Guyot J-P, et al. Optimization of 3D-Visualization of Micro-Anatomical Structures of the Human Inner Ear in Osmium Tetroxide Contrast Enhanced Micro-CT Scans. Front Neuroanat. Frontiers Media SA; 2018;12.
