# Supplementary material for: Evaluating hearing performance with cochlear implants within the same patient using daily randomization and imaging-based fitting - The ELEPHANT study
Source: Trials. 2020 Jun 23;21:564. doi: 10.1186/s13063-020-04469-x (PMC7310427; doi:10.1186/s13063-020-04469-x)
Supplement: Supplementary file 2 — Additional file 2. Case report form. [file 13063_2020_4469_MOESM2_ESM.pdf]

Case Report Form (CRF)

Study number: METC 18-028
Study title: ELEctric Place-pitched Hearing Achieves Natural Tonotopy (ELEPHANT)
Study site: Maastricht MUMC +

ELEPHANT-study

CRF Instructions

1. Verify if informed consent form (separate IC document) is signed by both patient and researcher before inclusion starts. The IC form should be locked and stored separate from this CRF. A signed copy of the IC form is given to the patient. An IC note is made to the patient's electronic medical dossier (SAP).
2. All questions in this CRF should be filled in, preferably with black ink.
3. The patient identification number (defined at p. 4) should be noted on every page of the CRF.
4. If data needs to be corrected, then these revisions should be provided with addition of a name and signature of the researcher. Corrected data should be crossed through but needs to stay readable. No correction fluid should be used.
5. When a test condition has been assessed, the corresponding box should be checked by an X.
6. If a test condition is not applicable, and thereby not assessed, the corresponding box should be crossed out.
7. If a test condition is applicable, but was not assessed, the corresponding box should be checked by an O.
8. Dates should be filled in using the format date/month/year: DD/MM/(20)YY

Glossary

<u>CI: cochlear implant</u>

- Standard program:
  frequency-allocation table is mapped conventionally, following clinical routine, based on a one-size fits-all principle, programmed on either processor Δ or O.
- Natural program:
  frequency-allocation table is mapped experimentally, following individual natural tonotopy, based on imaging data, programmed on either processor Δ or O.
- Preferred program :
  either standard or natural map, as decided by shared decision making during visit F14 (week 26).

<u>HA: hearing aid</u>

- Bimodal fit:
  settings that are programmed conventionally, following clinical gain rules based on patient's audiogram.
- Loudness fit:
  settings that are programmed experimentally, based on the acoustic loudness growth between threshold (T) and maximum (M) level.

<u>Overall</u>

- Clinical program:
  CI frequency-allocation table or HA settings that are mapped according to the obtained preferences during the study period, but programmed with clinical software.

INFORMED CONSENT

**Subject ID:**	E	P		
**Year of birth:**				

Date of subject's written informed consent: …………/……………/……………

Inclusion Criteria

	Yes	No*
adult (>18 years of age) and meeting the Dutch standard CI criteria		
proficient speaker of Dutch language		
post-lingual onset of profound deafness (> 4 years of age)		
subject receives an Advanced Bionics HiRes Ultra implant with Midscala electrode and an Advanced Bionics processor		
prepared to use study specific hearing aid (Phonak) for the duration of the study (in case of contralateral HA)		
rehabilitation at MUMC+ for the first year after surgery regarding CI as well as HA		
active participation in trial related procedures such as daily randomization and regular testing		
agreed to participate in this part of the study (by informed consent)		

*If any inclusion criteria are ticked no then the patient is not eligible for the study.

Exclusion Criteria

	Yes*	No
physical or non-physical contraindications for MRI or CT imaging		
additional disabilities that may prevent active participation**		
cochlear or neural abnormalities that could affect outcome measures and/or compromise the placement of the electrode array as assessed by the CI surgeon		
active participation in another prospective clinical trial		
pregnancy		
requirement of electric-acoustic activation prior to the first year follow-up		
having received a cochlear implant earlier (e.g. explantation or bilateral implantation)		

* If any exclusion criteria are ticked yes then the patient is not eligible for the study.

** If there are indications that the mental abilities to comply with the study procedures are insufficient, additional screening will be performed with the Mini-Mental State Examination. Patients will be excluded from the study when the resulting score is lower than 24;

BASELINE DATA

LEFT hearing situation

• Etiology of deafness	☐ Unknown/Progressive<br>☐ Other:
• Onset of profound deafness (age)	
• Current HA-use?	Yes / No
○ If yes, since age	
○ If yes, brand and type device	
○ If yes, age current device	years
○ If yes, frequency of usage	< 5 / 5-10 / > 10 hours/day

RIGHT hearing situation

• Etiology of deafness	☐ Unknown/Progressive<br>☐ Other:
• Onset of profound deafness (age)	
• Current HA-use?	Yes / No
○ If yes, since age	
○ If yes, brand and type device	
○ If yes, age current device	years
○ If yes, frequency of usage	< 5 / 5-10 / > 10 hours/day

Tinnitus Questionnaire (TQ)

• Total score	
• Grade	1 / 2 / 3 / 4

Overall

• Education level	☐ None<br>☐ Primary school<br>☐ Special education<br>☐ LBO/LTS<br>☐ ULO/MBO/MAVO<br>☐ HAVO/VWO<br>☐ HBO/HEAO/HTS<br>☐ Higher science education
• Native language	Dutch / other:
○ If other, Dutch language level	Very good / good / average / limited
• Current use of medication	Yes / No
○ If yes, type, dose and indication	

1. Pre-fit phase

1.1. P1/P2: Candidacy visits (and pre-OP, if applicable)

1.1.1. IMAGING

CT performed? ☐ Yes, date: ..../......./........ ☐ No

MRI performed? ☐ Yes, date: ..../......./........ ☐ No

Remarks

1.1.2. TONE AUDIOMETRY

	Unaided		HA		Unaided retest	
Date	......./......./........		......./......./........		......./......./........	
Ear	L	R	L	R	L	R
Completed?	☐	☐	☐	☐	☐	☐

Remarks

1.1.3. SPEECH AUDIOMETRY

	Unaided		HA		Unaided retest	
Date	......./......./........		......./......./........		......./......./........	
Ear	L	R	L	R	L	R
Completed?	☐	☐	☐	☐	☐	☐

Remarks

1.1.4. REM

REM performed? ☐ Yes, date: ..../......./........ ☐ No, because..

HA refit necessary? ☐ No ☐ Yes, refit on date: ..../......./........

Retest after refit? ☐ No ☐ Yes, retest on date: ..../......./........

	Tone audio		Speech audio	
	HA		HA	
Ear	L	R	L	R
Completed?	☐	☐	☐	☐

Remarks

1.1.5. EARMOULD

Adequate earmould available for contralateral ear? ☐ Yes, date: ……./……./…….. ☐ No, describe in remarks

If not, prescription given? ☐ Yes, date: ……./……./…….. ☐ No

Remarks

1.1.6. QUESTIONNAIRES

	Given to subject	Returned and checked
Date	……./……./……	……./……./……
SSQ-12 (I07)	☐	☐
HUI-3 (I08)	☐	☐
ICECAP-A (09)	☐	☐

Remarks

1.1.7. HARDWARE

Patient choice for CI color? ☐ Silver gray ☐ Chestnut ☐ Sand beige ☐ Velvet black ☐ Alpine white

Patient choice for HA color?

- ☐ Silver gray
- ☐ Chestnut
- ☐ Sand beige
- ☐ Velvet black
- ☐ Graphite grey

Remarks

1.1.8. PROTOCOL DEVIATIONS

Were there any protocol deviations? ☐ Yes ☐ No

Remarks

General remarks

1.2. P3: Visit OR

Date: …………/……………/……………

1.2.1. IMPLANTATION REPORT

Implantation report (I01) completed and signed by surgeon? ☐ Yes ☐ No

Intraoperative measurements performed? ☐ Yes ☐ No

Particularities that will influence CI fitting? ☐ No ☐ Yes, describe in remarks and note on CRF in section 2.1.1 and 2.2.2!

Remarks

1.2.2. ADVERSE EVENTS

Checked and acted on adverse events? ☐ Yes ☐ No

Remarks

General remarks

1.3. P4: Visit CBCT

Date: …………/…………../…………..

1.3.1. VISIT ENT

Any particularities? ☐ No ☐ Yes, describe:

……………………………………………………………………………………

……………………………………………………………………………………

1.3.2. CBCT SCAN

CBCT scan performed? ☐ Yes ☐ No, because……………………… → EARLY TERMINATION!

Scan quality adequate? ☐ Yes ☐ No, new scan performed on ……./……/…….

Particularities that will influence CI fitting? ☐ No ☐ Yes, describe in remarks and note on CRF in section 2.1.1 and 2.2.2!

Remarks

1.3.3. EARMOULD

Adequate earmould available for contralateral ear?

☐ Yes ☐ No, describe steps to be taken before visit F1:

……………………………………………………………………………………

……………………………………………………………………………………

1.3.4. PROTOCOL DEVIATIONS

Were there any protocol deviations? ☐ Yes ☐ No

Remarks

1.3.5. DEVICE DEFICIENCIES

Were there any device deficiencies? ☐ Yes ☐ No

Remarks

1.3.6. ADVERSE EVENTS

Checked and acted on adverse events? ☐ Yes ☐ No

Remarks

General remarks

2. First phase

2.1. Pre-fitting preparation

2.1.1. MAPPING

Mapping performed? ☐ Yes, date: ……./……./…….. ☐ No

Electrodes extracochlear? ☐ No ☐ Yes, el………………………

Electrodes folded over in tip? ☐ No ☐ Yes, el……………………..

Impedances within normal range (1.0 kΩ ≤ value ≤ 30.0 kΩ)?

El number	1	2	3	4	5	6	7	8	9	10	11	12	13	14	15	16
Y(es)/N(o)																

Electrodes to be turned off (based on impedances)? ☐ No ☐ Yes, el………………………

➔ To be applied in standard AND natural map!

➔ Note on section 2.2.2 and first fitting form!

Number of channels mapped < 8598 Hz (excluding phantom)? ☐ ≥ 8 ☐ < 8 → EARLY TERMINATION!

Remarks

2.1.2. DAILY RANDOMIZATION

Randomization schedule created? ☐ Yes, date: ……./……./…….. ☐ No

Any particularities? ☐ No ☐ Yes, describe:

……………………………………………………………………………………

……………………………………………………………………………………

Remarks

2.1.3. TEST RANDOMIZATION

Randomization of test conditions and word lists performed?

☐ Yes, date: ……./……./…….. ☐ No

Any particularities? ☐ No ☐ Yes, describe:

……………………………………………………………………………………

…………………………………………………………………………………

Remarks

2.1.4. HARDWARE

Patient kit ready? ☐ Yes, date: ……./……./…….. ☐ No

Patient processor stored safely? ☐ Yes, date: ……./……./…….. ☐ No

Contralateral earmould available? ☐ Yes, date: ……./……./…….. ☐ No

	CI		HA
Device	Research CI Naida Q70 Δ	Research Naida Q70 O	Research Naida Link
Serial number	…………………………………	…………………………………	…………………………………
Color	…………………………………	…………………………………	…………………………………

Remarks

2.1.5. SOFTWARE

Measurement template ready in BEPS+? ☐ Yes, date: ……./……./…….. ☐ No

Natural template ready in BEPS+? ☐ Yes, date: ……./……./…….. ☐ No

Audiogram ready in Phonak Target? ☐ Yes, date: ……./……./…….. ☐ No

Remarks

2.2. Visit F1 (wk 0)

Date: ………../…………../…………..

Bimodal user: ☐ Yes ☐ No, because………………………………………………………………………..

2.2.1. TONE AUDIOMETRY

	Unaided	
Ear	L	R
Completed?	☐	☐

Decided to start with EAS? ☐ No ☐ Yes → EARLY TERMINATION!

Remarks

2.2.2. SPEECH AUDIOMETRY

	Unaided	
Ear	L	R
Completed?	☐	☐

Remarks

2.2.3. FITTING

Particularities to be taken into account during fitting:

from impedance measurements? ☐ No ☐ Yes, ……………………………………………..

from ECAP measurements? ☐ No ☐ Yes, ……………………………………………..

from CBCT scan? ☐ No ☐ Yes, ……………………………………………..

Fitting form (i02) completed? ☐ No ☐ Yes

Particularities relevant for next fitting? ☐ No ☐ Yes, see fitting form.

Remarks

BREAK

2.2.4. SPEECH AUDIOMETRY

	Test			Retest	Test			Retest	Test			Retest
Listening	CI			CI	CI			CI	HA			HA
CI processor	Δ			Δ	O			O	CI turned off			
CI program	……				……				CI turned off			
HA program	HA turned off								Bimodal fit			
Level (dB SPL)	75	65	55	65	75	65	55	65	75	65	55	65
Completed?	☐	☐	☐	☐	☐	☐	☐	☐	☐	☐	☐	☐

Remarks

☐ Additional testing performed with number lists

2.2.5. MATRIX IN QUIET

	Practice		Test	Retest	Test	Retest
Listening	CI		CI	CI	CI	CI
CI processor	Δ / O		Δ	Δ	O	O
CI program	……		……		……	
HA program	HA turned off					
N sentences	10	10	10	10	10	10
Word list nr.	……	……	……	……	……	……
Order	1		……	……	……	……
Completed?	☐	☐	☐	☐	☐	☐

Remarks

☐ Could not be performed (too difficult for patient)

2.2.6. MATRIX IN NOISE

	Practice		Test	Retest	Test	Retest
Listening	CI		CI	CI	CI	CI
CI processor	Δ / O		Δ	Δ	O	O
CI program	……		……		……	
HA program	HA turned off					
N sentences	10	10	10	10	10	10
Word list nr.	……	……	……	……	……	……
Order	1		……	……	……	……
Completed?	☐	☐	☐	☐	☐	☐

Remarks

☐ Not performed, due to matrix in quiet SRT score = < 50%

2.2.7. PREFERENCE SCALES

Preference scales (i03) completed? ☐ Yes ☐ No

2.2.8. DATA MANAGEMENT CHECK-UP

Digital data complete and correctly archived on network drive? ☐ Yes ☐ No

→ If no, describe steps to be taken:

……

……

Source documents correctly archived in filing cabinet? ☐ Yes ☐ No

→ If no, describe steps to be taken:

……

……

Remarks

2.2.9. PROTOCOL DEVIATIONS

Were there any protocol deviations? ☐ Yes ☐ No

Remarks

2.2.10. DEVICE DEFICIENCIES

Were there any device deficiencies?	☐ Yes	☐ No

Remarks

2.2.11. ADVERSE EVENTS

Checked and acted on adverse events?	☐ Yes	☐ No
Any signs of tinnitus?	☐ Yes	☐ No
If yes, on what side(s)	☐ Left	☐ Right
If yes, to what extent (VAS 0-10)?	Left: ………..	Right: ………….
Any signs of vertigo?	☐ Yes	☐ No
Any signs of oscillopsia?	☐ Yes	☐ No

Remarks

General remarks

2.3. Visit F2 (week 1)

Date: ..../............../..............

Bimodal user: ☐ Yes ☐ No, because..

2.3.1. PREFERENCE SCALES

Preference scales (i03) completed? ☐ Yes ☐ No

2.3.2. FITTING

Particularities from previous fitting to be taken into account? ☐ No ☐ Yes, see fitting form

Fitting form (i02) completed? ☐ Yes ☐ No

Particularities relevant for next fitting? ☐ No ☐ Yes, note on fitting form

Compliance forms completed? ☐ Yes ☐ No

ECAP completed? ☐ Yes ☐ No

Remarks

2.3.3. SPEECH THERAPY

Particularities from speech therapy to be taken into account? ☐ No ☐ Yes, note here

....

Remarks

BREAK

2.3.4. SPEECH AUDIOMETRY

	Test			Retest	Test			Retest
Listening	CI			CI	CI			CI
CI processor	Δ			Δ	O			O
CI program	......				......			
HA program	HA turned off							
Level (dB SPL)	75	65	55	65	75	65	55	65
Completed?	☐	☐	☐	☐	☐	☐	☐	☐

Remarks

☐ Additional testing performed with number lists

2.3.5. MATRIX IN QUIET

	Practice		Test	Retest	Test	Retest
Listening	CI		CI	CI	CI	CI
CI processor	Δ / O		Δ	Δ	O	O
CI program	……		……		……	
HA program	HA turned off					
N sentences	10	10	10	10	10	10
Word list nr.	……	……	……	……	……	……
Order	1		……	……	……	……
Completed?	☐	☐	☐	☐	☐	☐

Remarks

2.3.6. MATRIX IN NOISE

	Practice		Test	Retest	Test	Retest
Listening	CI		CI	CI	CI	CI
CI processor	Δ / O		Δ	Δ	O	O
CI program	……		……		……	
HA program	HA turned off					
N sentences	10	10	10	10	10	10
Word list nr.	……	……	……	……	……	……
Order	1		……	……	……	……
Completed?	☐	☐	☐	☐	☐	☐

Remarks

☐ Not performed, due to matrix in quiet SRT score = < 50%

2.3.7. PROTOCOL DEVIATIONS

Were there any protocol deviations? ☐ Yes ☐ No

Remarks

SUBJECT ID:___________ SEEN BY MONITOR: ___________________ DATE: _____/_____/_______

2.3.8. DEVICE DEFICIENCIES

Were there any device deficiencies? ☐ Yes ☐ No

Remarks

2.3.9. ADVERSE EVENTS

Checked and acted on adverse events? ☐ Yes ☐ No

Remarks

General remarks

2.4. Visit F3 (week 2)

Date: ………../…………../…………..

Bimodal user: ☐ Yes ☐ No, because……………………………………………………………………….

2.4.1. TONE AUDIOMETRY

	CI		HA
Processor	Δ	O	Bimodal fit
CI program	……	……	CI off
Completed?	☐	☐	☐

Remarks

2.4.2. REM

Test completed? ☐ No ☐ Yes

Changes made to HA settings as a result of REM? ☐ No ☐ Yes, see fitting form

Remarks

2.4.3. PREFERENCE SCALES

Preference scales (i03) completed? ☐ Yes ☐ No

2.4.4. FITTING

Particularities from previous fitting to be taken into account? ☐ No ☐ Yes, see fitting form

Fitting form (i02) completed? ☐ Yes ☐ No

Particularities relevant for next fitting? ☐ No ☐ Yes, note on fitting form

Compliance forms completed? ☐ Yes ☐ No

Remarks

2.4.5. SPEECH THERAPY

Particularities from speech therapy to be taken into account? ☐ No ☐ Yes, note here

………

Remarks

BREAK

SUBJECT ID:___________ SEEN BY MONITOR: ___________________ DATE: _____/_____/_______

2.4.6. QUESTIONNAIRES

	Given to subject	To be returned
Date	……./……./……	Visit F4
Sound quality descriptors (I11)	☐	

Remarks

2.4.7. SPEECH AUDIOMETRY

	Test			Retest	Test			Retest	Test			Retest
Listening	CI			CI	CI			CI	HA			HA
CI	Δ			Δ	O			O	CI turned off			
CI program	……				……				CI turned off			
HA program	HA turned off								Bimodal fit			
Level (dB	75	65	55	65	75	65	55	65	75	65	55	65
Completed?	☐	☐	☐	☐	☐	☐	☐	☐	☐	☐	☐	☐

Remarks

☐ Additional testing performed with number lists

2.4.8. MATRIX IN QUIET

	Practice		Test	Retest	Test	Retest
Listening	CI		CI	CI	CI	CI
CI processor	Δ / O		Δ	Δ	O	O
CI program	……		……		……	
HA program	HA turned off					
N sentences	10	10	10	10	10	10
Word list nr.	……	……	……	……	……	……
Order	1		……	……	……	……
Completed?	☐	☐	☐	☐	☐	☐

Remarks

2.4.9. MATRIX IN NOISE

	Practice		Test	Retest	Test	Retest
Listening	CI		CI	CI	CI	CI
CI processor	Δ / O		Δ	Δ	O	O
CI program	……		……		……	
HA program	HA turned off					
N sentences	10	10	10	10	10	10
Word list nr.	……	……	……	……	……	……
Order	1		……	……	……	……
Completed?	☐	☐	☐	☐	☐	☐

Remarks

☐ Not performed, due to matrix in quiet SRT score = < 50%

2.4.10. PROTOCOL DEVIATIONS

Were there any protocol deviations? ☐ Yes ☐ No

Remarks

2.4.11. DEVICE DEFICIENCIES

Were there any device deficiencies? ☐ Yes ☐ No

Remarks

2.4.12. ADVERSE EVENTS

Checked and acted on adverse events? ☐ Yes ☐ No

Remarks

General remarks

2.5. Visit F4 (week 3)

Date: ..../............../..............

Bimodal user: ☐ Yes ☐ No, because...

2.5.1. PREFERENCE SCALES

Preference scales (i03) completed? ☐ Yes ☐ No

2.5.2. FITTING

Particularities from previous fitting to be taken into account? ☐ No ☐ Yes, see fitting form

Fitting form (i02) completed? ☐ Yes ☐ No

Particularities relevant for next fitting? ☐ No ☐ Yes, note on fitting form

Compliance forms completed? ☐ Yes ☐ No

Consequences of CI compliance measurements, based on calculations (fitting form) and cut-off points (Appendix Compliance):

☐ Study continuation ☐ Early termination and transfer to clinical care

Remarks

2.5.3. SPEECH THERAPY

Particularities from speech therapy to be taken into account? ☐ No ☐ Yes, note here

....

Remarks

2.5.4. SPEECH AUDIOMETRY

	Test			Retest	Test			Retest	Test			Retest
Listening	CI			CI	CI			CI	HA			HA
CI processor	Δ			Δ	O			O	CI turned off			
CI program	......				......				CI turned off			
HA program	HA turned off								Bimodal fit			
Level (dB SPL)	75	65	55	65	75	65	55	65	75	65	55	65

Completed?	☐	☐	☐	☐	☐	☐	☐	☐	☐	☐	☐	☐

	Test			Retest	Test			Retest
Listening	CIHA			CIHA	CIHA			CIHA
CI processor	Δ			Δ	O			O
CI program	……				……			
HA program	Bimodal fit							
Level (dB SPL)	55	65	75	65	55	65	75	65
Completed?	☐	☐	☐	☐	☐	☐	☐	☐

Remarks

2.5.5. MATRIX IN QUIET

	Practice		Test	Retest	Test	Retest
Listening	CI		CI	CI	CI	CI
CI processor	Δ / O		Δ	Δ	O	O
CI program	……		……		……	
HA program	HA turned off					
N sentences	10	10	10	10	10	10
Word list nr.	……	……	……	……	……	….
Order	1		……	……	……	……
Completed?	☐	☐	☐	☐	☐	☐

Remarks

BREAK

2.5.6. QUESTIONNAIRES

	Returned and checked?
Date	......./......./......
Sound quality descriptors (I11)	☐

Remarks

2.5.7. MATRIX IN NOISE

	Practice		Test	Retest	Test	Retest	Test	Retest	Test	Retest
Listening	CI / CIHA		CI	CI	CIHA	CIHA	CI	CI	CIHA	CIHA
CI processor	Δ / O		Δ	Δ	Δ	Δ	O	O	O	O
CI program	......		......		......		......		......	
HA program	Off / bim		HA off		Bimodal	Bimodal	HA off		Bimodal	Bimodal
N sentences	10	10	10	10	10	10	10	10	10	10
Word list nr.	......	......	......	......	......	....	......	......	....	....
Order	......		......	......	......	......	......	......	......	......
Completed?	☐	☐	☐	☐	☐	☐	☐	☐	☐	☐
**Test result** (SRT in dB SNR)	......	......	......	......	......	......	......	......	......	......
**Mean result** (SRT in dB SNR)	......		......		......		......		......	

Remarks

☐ Not performed, due to matrix in quiet SRT score = < 50%

2.5.8. PROTOCOL DEVIATIONS

Were there any protocol deviations? ☐ Yes ☐ No

Remarks

2.5.9. DEVICE DEFICIENCIES

Were there any device deficiencies? ☐ Yes ☐ No

Remarks

2.5.10. ADVERSE EVENTS

Checked and acted on adverse events? ☐ Yes ☐ No

Remarks

General remarks

2.6. Visit F5 (week 4)

Date: …………/…………../…………..

Bimodal user: ☐ Yes ☐ No, because…………………………………………………………………

2.6.1. SPEECH THERAPY

Particularities from speech therapy to be taken into account? ☐ No ☐ Yes, note here

……

Remarks

2.6.2. PREFERENCE SCALES

Preference scales (i03) completed? ☐ Yes ☐ No

2.6.3. CHECK-UP FITTING

Preference scales (i03) completed? ☐ Yes ☐ No

Fitting form datalogging & impedances (i02) completed? ☐ Yes ☐ No

Compliance forms completed? ☐ Yes ☐ No

Remarks

2.6.4. SPEECH AUDIOMETRY

	Test			Retest	Test			Retest
Listening	CI			CI	CI			CI
CI processor	Δ			Δ	O			O
CI program	……				……			
HA program	HA turned off							
Level (dB SPL)	75	65	55	65	75	65	55	65
Completed?	☐	☐	☐	☐	☐	☐	☐	☐

Remarks

2.6.5. MATRIX IN QUIET

	Practice		Test	Retest	Test	Retest
Listening	CI		CI	CI	CI	CI
CI processor	Δ / O		Δ	Δ	O	O
CI program	……		……		……	
HA program	HA off					
N sentences	10	10	10	10	10	10
Word list nr.	……	……	……	……	……	….
Order	1		2	3	4	5
Completed?	☐	☐	☐	☐	☐	☐

Remarks

2.6.6. MATRIX IN NOISE

	Practice		Test	Retest	Test	Retest
Listening	CI		CI	CI	CI	CI
CI processor	Δ / O		Δ	Δ	O	O
CI program	……		……		……	
HA program	HA off					
N sentences	10	10	10	10	10	10
Word list nr.	……	……	……	……	……	….
Order	1		2	3	4	5
Completed?	☐	☐	☐	☐	☐	☐
**Test result** (SRT in dB SNR)	……	……	……	……	……	……
**Mean result** (SRT in dB SNR)			……		……	

Remarks

☐ Not performed, due to matrix in quiet SRT score = < 50%

BREAK

2.6.7. LISTENING EFFORT

	Practice	Test	Test	Test	Test
Listening	CI	CI	CI	CIHA	CIHA
CI processor	Δ / O	Δ	O	Δ	O
CI program	……	……	……	……	……
HA program	Off / bimodal	HA off		Bimodal fit	Bimodal fit
N	1	5	5	5	5
SRT input (in dB SNR)	……. ……. ……. ……. ……. …….	……. ……. ……. ……. ……. …….	……. ……. ……. ……. ……. …….	……. ……. ……. ……. ……. …….	……. ……. ……. ……. ……. …….
Order	……	……	……	……	……
Completed?	☐	☐	☐	☐	☐

Remarks

2.6.8. LOUDNESS SCALING

	Test	Test	Test
Listening	CI	CI	HA
CI processor	Δ	O	
CI program	……	……	CI off
HA program	HA off		Bimodal fit
N frequencies	4	4	4
Order	……	……	……
Completed?	☐	☐	☐

Remarks

2.6.9. SMRT

	Test	Retest	Test	Retest
Listening	CI	CI	CI	CI
CI processor	Δ	Δ	O	O
CI program	……		……	
HA program	HA turned off			
Completed?	☐	☐	☐	☐

Remarks

2.6.10. PROTOCOL DEVIATIONS

Were there any protocol deviations? ☐ Yes ☐ No

Remarks

2.6.11. DEVICE DEFICIENCIES

Were there any device deficiencies? ☐ Yes ☐ No

Remarks

2.6.12. ADVERSE EVENTS

Checked and acted on adverse events? ☐ Yes ☐ No

Remarks

General remarks

2.7. Visit F6 (week 5)

Date: …………/…………../…………..

Bimodal user: ☐ Yes ☐ No, because……………………………………………………………

2.7.1. SPEECH THERAPY

Particularities from speech therapy to be taken into account? ☐ No ☐ Yes, note here

……

Remarks

2.7.2. PREFERENCE SCALES

Preference scales (i03) completed? ☐ Yes ☐ No

2.7.3. CHECK-UP FITTING

Fitting form datalogging & impedances (i02) completed? ☐ Yes ☐ No

Compliance forms completed? ☐ Yes ☐ No

Remarks

BREAK

2.7.4. SPEECH AUDIOMETRY

	Test			Retest	Test			Retest
Listening	CI			CI	CI			CI
CI processor	Δ			Δ	O			O
CI program	……				……			
HA program	HA turned off							
Level (dB SPL)	75	65	55	65	75	65	55	65
Completed?	☐	☐	☐	☐	☐	☐	☐	☐

Remarks

2.7.5. MATRIX IN QUIET

	Practice		Test	Retest	Test	Retest
Listening	CI		CI	CI	CI	CI
CI processor	Δ / O		Δ	Δ	O	O
CI program	……		……		……	
HA program	HA turned off					
N sentences	10	10	10	10	10	10
Word list nr.	……	……	……	……	……	……
Order	1		2	3	4	5
Completed?	☐	☐	☐	☐	☐	☐

Remarks

2.7.6. MATRIX IN NOISE

	Practice		Test	Retest	Test	Retest
Listening	CI		CI	CI	CI	CI
CI processor	Δ / O		Δ	Δ	O	O
CI program	……		……		……	
HA program	HA turned off					
N sentences	10	10	10	10	10	10
Word list nr.	……	……	……	……	……	……
Order	1		……	……	……	……
Completed?	☐	☐	☐	☐	☐	☐

Remarks

☐ Not performed, due to matrix in quiet SRT score = < 50%

2.7.7. PROTOCOL DEVIATIONS

Were there any protocol deviations? ☐ Yes ☐ No

Remarks

2.7.8. DEVICE DEFICIENCIES

Were there any device deficiencies? ☐ Yes ☐ No

Remarks

2.7.9. ADVERSE EVENTS

Checked and acted on adverse events? ☐ Yes ☐ No

Remarks

General remarks

2.8. Visit F7 (week 6)

Date: …………/……………/……………

Bimodal user: ☐ Yes ☐ No, because……………………………………………………………

2.8.1. TONE AUDIOMETRY

	CI	
Program	Δ	O
CI program	……	……
Completed?	☐	☐

Remarks

2.8.2. PREFERENCE SCALES

Preference scales (i03) completed? ☐ Yes ☐ No

2.8.3. FITTING

Particularities from previous fitting to be taken into account? ☐ No ☐ Yes, see fitting form

Particularities relevant for next fitting? ☐ No ☐ Yes, note on fitting form

Fitting form (i02) completed? ☐ Yes ☐ No

Compliance forms completed? ☐ Yes ☐ No

Consequences of CI compliance measurements, based on calculations (fitting form) and cut-off points (Appendix Compliance):

☐ Study continuation ☐ Removed from CI per protocol population

☐ Early terminated and transferred to clinical care

Consequences of HA compliance measurements, based on calculations (fitting form) and cut-off points (Appendix Compliance):

☐ Study continuation ☐ No bimodal testing at F10 → Check 'No bimodal user at 2.10'

Remarks

2.8.4. SPEECH THERAPY

Particularities from speech therapy to be taken into account? ☐ No ☐ Yes, note here

………

Remarks

BREAK

2.8.5. SPEECH AUDIOMETRY

	Test			Retest	Test			Retest
Listening	CI			CI	CI			CI
CI processor	Δ			Δ	O			O
CI program	……				……			
HA program	HA turned off							
Level (dB SPL)	75	65	55	65	75	65	55	65
Completed?	☐	☐	☐	☐	☐	☐	☐	☐

Remarks

2.8.6. MATRIX IN QUIET

	Practice		Test	Retest	Test	Retest
Listening	CI		CI	CI	CI	CI
CI processor	Δ / O		Δ	Δ	O	O
CI program	……		……		……	
HA program	HA turned off					
N sentences	10	10	10	10	10	10
Word list nr.	……	……	……	……	……	……
Order	1		……	……	……	……
Completed?	☐	☐	☐	☐	☐	☐

Remarks

2.8.7. MATRIX IN NOISE

	Practice		Test	Retest	Test	Retest
Listening	CI		CI	CI	CI	CI
CI processor	Δ / O		Δ	Δ	O	O
CI program	……		……		……	
HA program	HA turned off					
N sentences	10	10	10	10	10	10
Word list nr.	……	……	……	……	……	……
Order	1		……	……	……	……
Completed?	☐	☐	☐	☐	☐	☐

Remarks

2.8.8. PROTOCOL DEVIATIONS

Were there any protocol deviations? ☐ Yes ☐ No

Remarks

2.8.9. DEVICE DEFICIENCIES

Were there any device deficiencies? ☐ Yes ☐ No

Remarks

2.8.10. ADVERSE EVENTS

Checked and acted on adverse events? ☐ Yes ☐ No

Remarks

General remarks

SUBJECT ID:___________ SEEN BY MONITOR: ___________________ DATE: _____/_____/_______

2.9. Visit F8 (week 7)

Date: …………/…………../…………..

Bimodal user: ☐ Yes ☐ No, because……………………………………………………………

2.9.1. SPEECH THERAPY

Particularities from speech therapy to be taken into account? ☐ No ☐ Yes, note here

………

Remarks

2.9.2. PREFERENCE SCALES

Preference scales (i03) completed? ☐ Yes ☐ No

2.9.3. CHECK-UP FITTING

Fitting form datalogging & impedances (i02) completed? ☐ Yes ☐ No

Compliance forms completed? ☐ Yes ☐ No

Remarks

BREAK

2.9.4. SPEECH AUDIOMETRY

	Test			Retest	Test			Retest
Listening	CI			CI	CI			CI
CI processor	Δ			Δ	O			O
CI program	……				……			
HA program	HA turned off							
Level (dB SPL)	75	65	55	65	75	65	55	65
Completed?	☐	☐	☐	☐	☐	☐	☐	☐

Remarks

2.9.5. MATRIX IN QUIET

	Practice		Test	Retest	Test	Retest
Listening	CI		CI	CI	CI	CI
CI processor	Δ / O		Δ	Δ	O	O
CI program	……		……		……	
HA program	HA off					
N sentences	10	10	10	10	10	10
Word list nr.	……	……	……	……	……	….
Order	1		……	……	……	……
Completed?	☐	☐	☐	☐	☐	☐

Remarks

2.9.6. MATRIX IN NOISE

	Practice		Test	Retest	Test	Retest
Listening	CI		CI	CI	CI	CI
CI processor	Δ / O		Δ	Δ	O	O
CI program	……		……		……	
HA program	HA turned off					
N sentences	10	10	10	10	10	10
Word list nr.	……	……	……	……	……	……
Order	1		……	……	……	……
Completed?	☐	☐	☐	☐	☐	☐

Remarks

2.9.7. PROTOCOL DEVIATIONS

Were there any protocol deviations? ☐ Yes ☐ No

Remarks

2.9.8. ADVERSE EVENTS

Checked and acted on adverse events? ☐ Yes ☐ No

Remarks

General remarks

2.10. Visit F9 (week 8)

Date: ………../…………./…………

Bimodal user: ☐ Yes ☐ No, because……………………………………………………………

2.10.1. SPEECH THERAPY

Particularities from speech therapy to be taken into account? ☐ No ☐ Yes, note here

……

Remarks

2.10.2. PREFERENCE SCALES

Preference scales (i03) completed? ☐ Yes ☐ No

2.10.3. CHECK-UP FITTING

Fitting form datalogging & impedances (i02) completed? ☐ Yes ☐ No

Compliance forms completed? ☐ Yes ☐ No

ECAP measured? ☐ Yes ☐ No

Remarks

2.10.4. SPEECH AUDIOMETRY

	Test			Retest	Test			Retest
Listening	CI			CI	CI			CI
CI processor	Δ			Δ	O			O
CI program	……				……			
HA program	HA turned off							
Level (dB SPL)	75	65	55	65	75	65	55	65
Completed?	☐	☐	☐	☐	☐	☐	☐	☐

Remarks

2.10.5. MATRIX IN QUIET

	Practice		Test	Retest	Test	Retest
Listening	CI		CI	CI	CI	CI
CI processor	Δ / O		Δ	Δ	O	O
CI program	……		……		……	
HA program	HA off					
N sentences	10	10	10	10	10	10
Word list nr.	……	……	……	……	……	….
Order	1		……	……	……	……
Completed?	☐	☐	☐	☐	☐	☐

Remarks

2.10.6. MATRIX IN NOISE

	Practice		Test	Retest	Test	Retest
Listening	CI		CI	CI	CI	CI
CI processor	Δ		Δ	Δ	O	O
CI program	……		……		……	
HA program	HA turned off					
N sentences	10	10	10	10	10	10
Word list nr.	……	……	……	……	……	……
Order	1		2	3	4	5
Completed?	☐	☐	☐	☐	☐	☐

Remarks

2.10.7. PROTOCOL DEVIATIONS

Were there any protocol deviations? ☐ Yes ☐ No

Remarks

2.10.8. DEVICE DEFICIENCIES

Were there any device deficiencies? ☐ Yes ☐ No

Remarks

2.10.9. ADVERSE EVENTS

Checked and acted on adverse events? ☐ Yes ☐ No

Remarks

General remarks

2.11. Visit F10 (week 10)

Date: …………/…………./………….

Bimodal user: ☐ Yes ☐ No, because……………………………………………………………

2.11.1. SPEECH THERAPY

Particularities from speech therapy to be taken into account? ☐ No ☐ Yes, note here

……

Remarks

2.11.2. PREFERENCE SCALES

Preference scales (i03) completed? ☐ Yes ☐ No

2.11.3. CHECK-UP FITTING

Fitting form datalogging & impedances (i02) completed? ☐ Yes ☐ No

Compliance forms completed? ☐ Yes ☐ No

Remarks

2.11.4. QUESTIONNAIRES

	Given to subject	To be returned
Date	……./……./……	Visit F11
Sound quality descriptors (I11)	☐	

Remarks

2.11.5. SPEECH AUDIOMETRY

	Test			Retest	Test			Retest	Test			Retest
Listening	CI			CI	CI			CI	HA			HA
CI processor	Δ			Δ	O			O	CI turned off			
CI program	……				……				CI turned off			
HA program	HA turned off								Bimodal fit			
Level (dB SPL)	75	65	55	65	75	65	55	65	75	65	55	65

Completed?	☐	☐	☐	☐	☐	☐	☐	☐	☐	☐	☐	☐
	Test			Retest	Test			Retest				
Listening	CIHA			CIHA	CIHA			CIHA				
CI processor	Δ			Δ	O			O				
CI program	……				……							
HA program	Bimodal fit											
Level (dB SPL)	55	65	75	65	55	65	75	65				
Completed?	☐	☐	☐	☐	☐	☐	☐	☐				

Remarks

BREAK

2.11.6. MATRIX IN QUIET

	Practice		Test	Retest	Test	Retest
Listening	CI		CI	CI	CI	CI
CI processor	Δ / O		Δ	Δ	O	O
CI program	……		……		……	
HA program	HA turned off					
N sentences	10	10	10	10	10	10
Word list nr.	……	……	……	……	……	……
Order	1		……	……	……	……
Completed?	☐	☐	☐	☐	☐	☐

Remarks

2.11.7. MATRIX IN NOISE

	Practice		Test	Retest	Test	Retest	Test	Retest	Test	Retest
Listening	CI / CIHA		CI	CI	CIHA	CIHA	CI	CI	CIHA	CIHA
CI processor	Δ / O		Δ	Δ	Δ	Δ	O	O	O	O
CI program	……		……		……		……		……	
HA program	Off / bimodal		HA turned off		Bimodal fit	Bimodal fit	HA turned off		Bimodal fit	Bimodal fit
N sentences	10	10	10	10	10	10	10	10	10	10
Word list	……	……	……	……	……	……	……	……	……	……
Order	……		……	……	……	……	……	……	……	……
Completed?	☐	☐	☐	☐	☐	☐	☐	☐	☐	☐
**Test result** (SRT in dB SNR)	……	……	……	……	……	……	……	……	……	……
**Mean result** (SRT in dB SNR)	……		……		……		……		……	

Remarks

BREAK

2.11.8. MATRIX IN NOISE SPATIAL

	Test	Retest	Test	Retest	Test	Retest	Test	Retest
Listening	CI	CI	CI	CI	CI	CI	CI	CI
Spatial	S0NCI	S0NCI	S0NHA	S0NHA	S0NCI	S0NCI	S0NHA	S0NHA
CI processor	Δ	Δ	Δ	Δ	O	O	O	O
CI program	……		……		……		……	
HA program	HA turned off							
N sentences	10	10	10	10	10	10	10	10
Hammerfall	……	……	……	……	……	……	……	……
Word list	……	……	……	……	……	……	……	……
Order	……	……	……	……	……	……	……	……
Completed?	☐	☐	☐	☐	☐	☐	☐	☐
	Test	Retest	Test	Retest	Test	Retest	Test	Retest
Listening	CIHA	CIHA	CIHA	CIHA	CIHA	CIHA	CIHA	CIHA
Spatial	S0NCI	S0NCI	S0NHA	S0NHA	S0NCI	S0NCI	S0NHA	S0NHA
CI processor	Δ	Δ	Δ	Δ	O	O	O	O
CI program	……		……		……		……	
HA program	Bimodal fit	Bimodal fit	Bimodal fit	Bimodal fit	Bimodal fit	Bimodal fit	Bimodal fit	Bimodal fit
N sentences	10	10	10	10	10	10	10	10
Preset	……	……	……	……	……	……	……	……
Word list	……	……	……	……	……	……	……	……
Order	……	……	……	……	……	……	……	……
Completed?	☐	☐	☐	☐	☐	☐	☐	☐

Remarks

2.11.9. PROTOCOL DEVIATIONS

Were there any protocol deviations? ☐ Yes ☐ No

Remarks

2.11.10. DEVICE DEFICIENCIES

Were there any device deficiencies? ☐ Yes ☐ No

Remarks

2.11.11. ADVERSE EVENTS

Checked and acted on adverse events? ☐ Yes ☐ No

Remarks

General remarks

2.12. Visit F11 (week 12)

Date: …………/…………./………….

Bimodal user: ☐ Yes ☐ No, because……………………………………………………………

2.12.1. LISTENING EFFORT

	Practice	Test	Test	Test	Test
Listening	CI / CIHA	CI	CI	CIHA	CIHA
CI processor	Δ / O	Δ	O	Δ	O
CI program	……	……	……	……	……
HA program	Off / bimodal	HA off		Bimodal fit	Bimodal fit
N	1	5	5	5	5
SRT input (in dB SNR)<br>-> See visit F10	……. ……. ……<br>……. ……. …….	…… …… ……<br>…… …… ……	…… …… ……<br>…… …… ……	…… …… ……<br>…… …… ……	…… …… ……<br>…… …… ……
Order	……	……	……	……	……
Completed?	☐	☐	☐	☐	☐

Remarks

2.12.2. LOUDNESS SCALING

	Test	Test	Test
Listening	CI	CI	HA
CI processor	Δ	O	
CI program	……	……	CI off
HA program	HA off		Bimodal fit
N frequencies	4	4	4
Order	……	……	……
Completed?	☐	☐	☐

Remarks

2.12.3. SMRT

	Test	Retest	Test	Retest
Listening	CI	CI	CI	CI
CI processor	Δ	Δ	O	O
CI program	……		……	
HA program	HA turned off			
Completed?	☐	☐	☐	☐

Remarks

2.12.4. PREFERENCE SCALES

Preference scales (i03) completed? ☐ Yes ☐ No

2.12.5. FITTING

Particularities from previous fitting to be taken into account? ☐ No ☐ Yes, see fitting form

Fitting form (i02) completed? ☐ Yes ☐ No

Particularities relevant for next fitting? ☐ No ☐ Yes, note on fitting form

Compliance forms completed? ☐ Yes ☐ No

Consequences of CI compliance measurements, based on calculations (fitting form) and cut-off points (Appendix Compliance):

☐ Study continuation ☐ Removed from CI per protocol population

☐ Early terminated and transferred to clinical care

Remarks

2.12.6. SPEECH THERAPY

Particularities from speech therapy to be taken into account? ☐ No ☐ Yes, note here

……

Remarks

BREAK

2.12.7. SPEECH AUDIOMETRY

	Test			Retest	Test			Retest
Listening	CI			CI	CI			CI
CI processor	Δ			Δ	O			O
CI program	……				……			
HA program	HA turned off							
Level (dB SPL)	75	65	55	65	75	65	55	65
Completed?	☐	☐	☐	☐	☐	☐	☐	☐

Remarks

2.12.8. MATRIX IN QUIET

	Practice		Test	Retest	Test	Retest
Listening	CI		CI	CI	CI	CI
CI processor	Δ / O		Δ	Δ	O	O
CI program	……		……		……	
HA program	HA off					
N sentences	10	10	10	10	10	10
Word list nr.	……	……	……	……	……	….
Order	1		……	……	……	……
Completed?	☐	☐	☐	☐	☐	☐

Remarks

2.12.9. MATRIX IN NOISE

	Practice		Test	Retest	Test	Retest
Listening	CI		CI	CI	CI	CI
CI processor	Δ / O		Δ	Δ	O	O
CI program	……		……		……	
HA program	HA off					
N sentences	10	10	10	10	10	10
Word list nr.	……	……	……	……	……	….
Order	1		……	……	……	……
Completed?	☐	☐	☐	☐	☐	☐
**Test result** (SRT in dB SNR)	……	……	……	……	……	……
**Mean result** (SRT in dB SNR)			……		……	

Remarks

BREAK

2.12.10. QUESTIONNAIRES

	Returned and checked?
Date	……./……./……
Sound quality descriptors (I11)	☐

Remarks

2.12.11. PROTOCOL DEVIATIONS

Were there any protocol deviations? ☐ Yes ☐ No

Remarks

SUBJECT ID:____________ SEEN BY MONITOR: ____________________ DATE: _____/_____/_______

2.12.12. DEVICE DEFICIENCIES

Were there any device deficiencies? ☐ Yes ☐ No

Remarks

2.12.13. ADVERSE EVENTS

Checked and acted on adverse events? ☐ Yes ☐ No

Remarks

General remarks

2.13. Visit F12 (week 16)

Date: ………../…………./…………

Bimodal user: ☐ Yes ☐ No, because……………………………………………………………

2.13.1. PREFERENCE SCALES

Preference scales (i03) completed? ☐ Yes ☐ No

2.13.2. CHECK-UP FITTING

Fitting form datalogging & impedances (i02) completed? ☐ Yes ☐ No

Compliance forms completed? ☐ Yes ☐ No

Remarks

2.13.3. SPEECH AUDIOMETRY

	Test			Retest	Test			Retest
Listening	CI			CI	CI			CI
CI processor	Δ			Δ	O			O
CI program	……				……			
HA program	HA turned off							
Level (dB SPL)	75	65	55	65	75	65	55	65
Completed?	☐	☐	☐	☐	☐	☐	☐	☐

Remarks

2.13.4. MATRIX IN QUIET

	Practice		Test	Retest	Test	Retest
Listening	CI		CI	CI	CI	CI
CI processor	Δ / O		Δ	Δ	O	O
CI program	……		……		……	
HA program	HA off					
N sentences	10	10	10	10	10	10
Word list nr.	……	……	……	……	……	….
Order	1		……	……	……	……
Completed?	☐	☐	☐	☐	☐	☐

Remarks

2.13.5. MATRIX IN NOISE

	Practice		Test	Retest	Test	Retest
Listening	CI		CI	CI	CI	CI
CI processor	Δ / O		Δ	Δ	O	O
CI program	……		……		……	
HA program	HA turned off					
N sentences	10	10	10	10	10	10
Word list nr.	……	……	……	……	……	……
Order	1		……	……	……	……
Completed?	☐	☐	☐	☐	☐	☐

Remarks

2.13.6. DATA MANAGEMENT CHECK-UP

Digital data complete and correctly archived on network drive? ☐ Yes ☐ No

→ If no, describe steps to be taken:

……

……

Source documents correctly archived in filing cabinet? ☐ Yes ☐ No

→ If no, describe steps to be taken:

……

……

Remarks

2.13.7. PROTOCOL DEVIATIONS

Were there any protocol deviations? ☐ Yes ☐ No

Remarks

2.13.8. DEVICE DEFICIENCIES

Were there any device deficiencies? ☐ Yes ☐ No

Remarks

2.13.9. ADVERSE EVENTS

Checked and acted on adverse events? ☐ Yes ☐ No

Any signs of tinnitus? ☐ Yes ☐ No

If yes, on what side(s) ☐ Left ☐ Right

If yes, to what extent (VAS 0-10)? Left: ……….. Right: …………..

Any signs of vertigo? ☐ Yes ☐ No

Any signs of oscillopsia? ☐ Yes ☐ No

Remarks

General remarks

2.14. Visit F13 (week 20)

Date: ………../…………./…………

Bimodal user: ☐ Yes ☐ No, because…………………………………………………………………

2.14.1. PREFERENCE SCALES

Preference scales (i03) completed? ☐ Yes ☐ No

2.14.2. FITTING

Particularities from previous fitting to be taken into account? ☐ No ☐ Yes, see fitting form

Fitting form (i02) completed? ☐ Yes ☐ No

Particularities relevant for next fitting? ☐ No ☐ Yes, note on fitting form

Compliance forms completed? ☐ Yes ☐ No

Remarks

2.14.3. SPEECH AUDIOMETRY

	Test			Retest	Test			Retest
Listening	CI			CI	CI			CI
CI processor	Δ			Δ	O			O
CI program	……				……			
HA program	HA turned off							
Level (dB SPL)	75	65	55	65	75	65	55	65
Completed?	☐	☐	☐	☐	☐	☐	☐	☐

Remarks

2.14.4. MATRIX IN QUIET

	Practice		Test	Retest	Test	Retest
Listening	CI		CI	CI	CI	CI
CI processor	Δ / O		Δ	Δ	O	O
CI program	……		……		……	
HA program	HA turned off					
N sentences	10	10	10	10	10	10
Word list nr.	……	……	……	……	……	……
Order	1		……	……	……	……

Completed?	☐	☐	☐	☐	☐	☐

Remarks

2.14.5. MATRIX IN NOISE

	Practice		Test	Retest	Test	Retest
Listening	CI		CI	CI	CI	CI
CI processor	Δ / O		Δ	Δ	O	O
HA program	HA turned off					
N sentences	10	10	10	10	10	10
Word list nr.	……	……	……	……	……	……
Order	1		……	……	……	……
Completed?	☐	☐	☐	☐	☐	☐

Remarks

2.14.6. PROTOCOL DEVIATIONS

Were there any protocol deviations? ☐ Yes ☐ No

Remarks

2.14.7. DEVICE DEFICIENCIES

Were there any device deficiencies? ☐ Yes ☐ No

Remarks

2.14.8. ADVERSE EVENTS

Checked and acted on adverse events? ☐ Yes ☐ No

Remarks

General remarks

2.15. Visit F14 (week 26)

Date: ..../............../..............

Bimodal user: ☐ Yes ☐ No, because..

2.15.1. TONE AUDIOMETRY

	CI		HA	Unaided	
Processor/Ear	Δ	O	Bimodal fit	L	R
CI program	......	......	CI off	CI off	CI off
Completed?	☐	☐	☐	☐	☐

Remarks

2.15.2. SPEECH AUDIOMETRY

	Unaided	
Ear	L	R
Completed?	☐	☐

Remarks

2.15.3. PREFERENCE SCALES

Preference scales (i03) completed? ☐ Yes ☐ No

2.15.4. FITTING

Program choice after shared decision making: ☐ Standard program ☐ Natural program

Fitting form (i02) completed? ☐ Yes ☐ No

Compliance forms completed? ☐ Yes ☐ No

Consequences of HA compliance measurements, based on calculations (fitting form) and cut-off points (Appendix Compliance):

☐ Study continuation ☐ Removed from HA population/bimodal group?

ECAP completed? ☐ Yes ☐ No

Spare processor taken in and stored? ☐ No ☐ Yes -> S/N: ....

Remarks

2.15.5. QUESTIONNAIRES

	Given to subject	To be returned
Date	......./......./......	Visit F15
SSQ-12 (I07)	☐	
HUI-3 (I08)	☐	
ICECAP-A (09)	☐	
Sound quality descriptors (I11)	☐	

Remarks

BREAK

2.15.6. PROTOCOL DEVIATIONS

Were there any protocol deviations? ☐ Yes ☐ No

Remarks

2.15.7. DEVICE DEFICIENCIES

Were there any device deficiencies? ☐ Yes ☐ No

Remarks

2.15.8. ADVERSE EVENTS

Checked and acted on adverse events? ☐ Yes ☐ No

Remarks

General remarks

2.16. Visit F15 (week 26)

Date: …………/……………/……………

Bimodal user: ☐ Yes ☐ No

2.16.1. SPEECH AUDIOMETRY

	Test			Retest	Test			Retest	Test			Retest
Listening	CI			CI	HA			HA	CIHA			CIHA
CI processor	Preferred			Preferred	CI turned off				Preferred			Preferred
CI program	……				CI turned off				……			
HA program	HA turned off				Bimodal fit				Bimodal fit			Bimodal fit
Level (dB SPL)	75	65	55	65	75	65	55	65	75	65	55	65
Completed?	☐	☐	☐	☐	☐	☐	☐	☐	☐	☐	☐	☐

Remarks

2.16.2. MATRIX IN QUIET

	Practice		Test	Retest
Listening	CI		CI	CI
CI processor	Preferred		Preferred	Preferred
CI program	……		……	
HA program	HA turned off			
N sentences	10	10	10	10
Word list nr	……	……	……	……
Order	1		2	3
Completed?	☐	☐	☐	☐

Remarks

2.16.3. MATRIX IN NOISE

	Practice		Test	Retest	Test	Retest
Listening	CI / CIHA		CI	CI	CIHA	CIHA
CI processor	Preferred		Preferred	Preferred	Preferred	Preferred
CI program	……		……		……	
HA program	Off / bimodal		HA turned off		Bimodal fit	Bimodal fit
N sentences	10	10	10	10	10	10
Word list nr	……	……	……	……	……	……
Order	……		……	……	……	……
Completed?	☐	☐	☐	☐	☐	☐
**Test result** (SRT in dB SNR)	……	……	……	……	……	……
**Mean result** (SRT in dB SNR)			……		……	

Remarks

BREAK

2.16.4. QUESTIONNAIRES

	Returned and checked
Date	……./……./……
SSQ-12 (I07)	☐
HUI-3 (I08)	☐
ICECAP-A (09)	☐
Sound quality descriptors (I11)	☐

Remarks

2.16.5. MATRIX IN NOISE SPATIAL

	Test	Retest	Test	Retest	Test	Retest	Test	Retest
Listening	CI	CI	CI	CI	CIHA	CIHA	CIHA	CIHA
Spatial	S0NCI	S0NCI	S0NHA	S0NHA	S0NCI	S0NCI	S0NHA	S0NHA
CI processor	Preferred	Preferred	Preferred	Preferred	Preferred	Preferred	Preferred	Preferred
CI program	……		……		……		……	
HA program	HA turned off				Bimodal fit	Bimodal fit	Bimodal fit	Bimodal fit
N sentences	10	10	10	10	10	10	10	10
Hammerfall Preset	……	……	……	……	……	……	……	……
Word list nr	……	……	……	……	……	……	……	……
Order	……	……	……	……	……	……	……	……
Completed?	☐	☐	☐	☐	☐	☐	☐	☐

Remarks

BREAK

2.16.6. LISTENING EFFORT

	Practice	Test	Test
Listening	CI / CIHA	CI	CIHA
CI processor	Preferred	Preferred	Preferred
CI program	……	……	……
HA program	Off / bimodal	HA turned off	Bimodal fit
SRT input (in dB SNR)	…….. …….. ……..<br>…….. …….. ……..	…….. …….. ……..<br>…….. …….. ……..	…….. …….. ……..<br>…….. …….. ……..
Order	……	……	……
Completed?	☐	☐	☐

Remarks

2.16.7. LOUDNESS SCALING

	Test	Test
Listening	CI	HA
CI processor	Preferred	CI turned off
CI program	……	CI turned off
HA program	HA turned off	Bimodal fit
N questions	4	4
Word list nr	……	……
Order	……	……
Completed?	☐	☐

Remarks

2.16.8. SMRT

	Test	Retest	Test	Retest
Listening	CI	CI	HA	HA
CI processor	Preferred	Preferred	CI turned off	
CI program	……		CI turned off	
HA program	HA turned off		Bimodal fit	
Completed?	☐	☐	☐	☐

Remarks

2.16.9. PROTOCOL DEVIATIONS

Were there any protocol deviations? ☐ Yes ☐ No

Remarks

2.16.10. DEVICE DEFICIENCIES

Were there any device deficiencies? ☐ Yes ☐ No

Remarks

2.16.11. ADVERSE EVENTS

Checked and acted on adverse events? ☐ Yes ☐ No

Remarks

General remarks

3. Second phase

3.1. Visit S1 (week 30)

Date: …………/……………/……………

Bimodal user: ☐ Yes ☐ No, because……………………………………………………………

3.1.1. PREFERENCE SCALES

Preference scales (i04) completed? ☐ Yes ☐ No

3.1.2. FITTING

Particularities from previous fitting to be taken into account? ☐ No ☐ Yes, see fitting form

Fitting form (i02) completed? ☐ Yes ☐ No

Compliance forms completed? ☐ Yes ☐ No

Particularities relevant for next fitting? ☐ No ☐ Yes, note on fitting form

Remarks

3.1.3. QUESTIONNAIRES

	Given to subject	To be returned
Date	……./……./……	Visit T1
SSQ-12 (I07)	☐	
HUI-3 (I08)	☐	
ICECAP-A (09)	☐	
Sound quality descriptors (I11)	☐	

Remarks

3.1.4. SPEECH AUDIOMETRY

	Test			Retest	Test			Retest	Test			Retest
Listening	CI			CI	HA			HA	CIHA			CIHA
CI processor	Preferred			Preferred	CI turned off				Preferred			Preferred
CI program	……				CI turned off				……			
HA program	HA turned off				Loudness fit				Loudness fit			Loudness fit
Level (dB SPL)	75	65	55	65	75	65	55	65	75	65	55	65
Completed?	☐	☐	☐	☐	☐	☐	☐	☐	☐	☐	☐	☐

Remarks

3.1.5. MATRIX IN QUIET

	Practice		Test	Retest
Listening	CI		CI	CI
CI processor	Preferred		Preferred	Preferred
CI program	……		……	
HA program	HA turned off			
N sentences	10	10	10	10
Word list nr	……	……	……	……
Order	1		2	3
Completed?	☐	☐	☐	☐

Remarks

3.1.6. MATRIX IN NOISE

	Practice		Test	Retest	Test	Retest
Listening	CI / CIHA		CI	CI	CIHA	CIHA
CI processor	Preferred		Preferred	Preferred	Preferred	Preferred
CI program	……		……		……	
HA program	Off / loudness		HA turned off		Loudness fit	Loudness fit
N sentences	10	10	10	10	10	10
Word list nr	……	……	……	……	……	……
Order	……		……	……	……	……
Completed?	☐	☐	☐	☐	☐	☐
**Test result** (SRT in dB SNR)	……	……	……	……	……	……
**Mean result** (SRT in dB SNR)			……		……	

Remarks

BREAK

3.1.7. MATRIX IN NOISE SPATIAL

	Test	Retest	Test	Retest	Test	Retest	Test	Retest
Listening	CI	CI	CI	CI	CIHA	CIHA	CIHA	CIHA
Spatial	S0NCI	S0NCI	S0NHA	S0NHA	S0NCI	S0NCI	S0NHA	S0NHA
CI processor	Preferred	Preferred	Preferred	Preferred	Preferred	Preferred	Preferred	Preferred
CI program	……		……		……		……	
HA program	HA turned off				Loudness fit	Loudness fit	Loudness fit	Loudness fit
N sentences	10	10	10	10	10	10	10	10
Hammerfall Preset	……	……	……	……	……	……	……	……
Word list nr	……	……	……	……	……	……	……	……
Order	……	……	……	……	……	……	……	……
Completed?	☐	☐	☐	☐	☐	☐	☐	☐

Remarks

3.1.8. LISTENING EFFORT

	Practice	Test	Test
Listening	CI / CIHA	CI	CIHA
CI processor	Preferred	Preferred	Preferred
CI program	……	……	……
HA program	Off / Loudness fit	HA turned off	Loudness fit
SRT input (in dB SNR)	…….. …….. ……..<br>…….. …….. ……..	…….. …….. ……..<br>…….. …….. ……..	…….. …….. ……..<br>…….. …….. ……..
Order	……	……	……
Completed?	☐	☐	☐

Remarks

3.1.9. LOUDNESS SCALING

	Test	Test
Listening	CI	HA
CI processor	Preferred	CI turned off
CI program	……	CI turned off
HA program	HA turned off	Loudness fit
N questions	4	4
Word list nr	……	……
Order	……	……
Completed?	☐	☐

Remarks

3.1.10. DATA MANAGEMENT CHECK-UP

Digital data complete and correctly archived on network drive? ☐ Yes ☐ No

→ If no, describe steps to be taken:

……

……

Source documents correctly archived in filing cabinet? ☐ Yes ☐ No

→ If no, describe steps to be taken:

……

……

Remarks

3.1.11. PROTOCOL DEVIATIONS

Were there any protocol deviations? ☐ Yes ☐ No

Remarks

3.1.12. DEVICE DEFICIENCIES

Were there any device deficiencies? ☐ Yes ☐ No

Remarks

3.1.13. ADVERSE EVENTS

Checked and acted on adverse events?	☐ Yes	☐ No
Any signs of tinnitus?	☐ Yes	☐ No
If yes, on what side(s)	☐ Left	☐ Right
If yes, to what extent (VAS 0-10)?	Left: ………..	Right: ………….
Any signs of vertigo	☐ Yes	☐ No
Any signs of oscillopsia?	☐ Yes	☐ No

Remarks

General remarks

4. Third phase

4.1. Visit T1 (week 34)

Date: …………/…………./………….

Bimodal user: ☐ Yes ☐ No, because………………………………………………………………

4.1.1. TONE AUDIOMETRY

	HA
Program	Loudness fit
Completed?	☐

Remarks

4.1.2. SPEECH AUDIOMETRY

	Test			Retest	Test			Retest	Test			Retest
Listening	CI			CI	HA			HA	CIHA			CIHA
CI processor	Preferred			Preferred	CI turned off				Preferred			Preferred
CI program	……				CI turned off				……			
HA program	HA turned off				Loudness fit				Loudness fit			Loudness fit
Level (dB SPL)	75	65	55	65	75	65	55	65	75	65	55	65
Completed?	☐	☐	☐	☐	☐	☐	☐	☐	☐	☐	☐	☐

Remarks

4.1.3. MATRIX IN QUIET

	Practice		Test	Retest
Listening	CI		CI	CI
CI processor	Preferred		Preferred	Preferred
CI program	……		……	
HA program	HA turned off			
N sentences	10	10	10	10
Word list nr	……	……	……	……
Order	1		2	3
Completed?	☐	☐	☐	☐

Remarks

4.1.4. MATRIX IN NOISE

	Practice		Test	Retest	Test	Retest
Listening	CI / CIHA		CI	CI	CIHA	CIHA
CI processor	Preferred		Preferred	Preferred	Preferred	Preferred
CI program	……		……		……	
HA program	Off / loudness		HA turned off		Loudness fit	Loudness fit
N sentences	10	10	10	10	10	10
Word list nr.	……	……	……	……	……	……
Order	……	……	……	……	……	……
Completed?	☐	☐	☐	☐	☐	☐
**Test result** (SRT in dB SNR)	……		……	……	……	……
**Mean result** (SRT in dB SNR)			……		……	

Remarks

4.1.5. QUESTIONNAIRES

	Returned and checked
Date	……./……./…...
SSQ-12 (I07)	☐
HUI-3 (I08)	☐
ICECAP-A (09)	☐
Sound quality descriptors (I11)	☐

Remarks

BREAK

4.1.6. MATRIX IN NOISE SPATIAL

	Test	Retest	Test	Retest	Test	Retest	Test	Retest
Listening	CI	CI	CI	CI	CIHA	CIHA	CIHA	CIHA
Spatial	S0NCI	S0NCI	S0NHA	S0NHA	S0NCI	S0NCI	S0NHA	S0NHA
CI processor	Preferred	Preferred	Preferred	Preferred	Preferred	Preferred	Preferred	Preferred
CI program	……		……		……		……	
HA program	HA turned off				Loudness fit	Loudness fit	Loudness fit	Loudness fit
N sentences	10	10	10	10	10	10	10	10
Hammerfall Preset	……	……	……	……	……	……	……	……
Word list nr	……	……	……	……	……	……	……	……
Order	……	……	……	……	……	……	……	……
Completed?	☐	☐	☐	☐	☐	☐	☐	☐

Remarks

4.1.7. LISTENING EFFORT

	Practice	Test	Test
Listening	CI / CIHA	CI	CIHA
CI processor	Preferred	Preferred	Preferred
CI program	……	……	……
HA program	Off / loudness	HA turned off	Loudness fit
SRT input (in dB SNR)	…….. …….. …….. …….. …….. …….	…….. …….. …….. …….. …….. ……..	…….. …….. …….. …….. …….. ……..
Order	……	……	……

Completed?	☐	☐	☐

Remarks

4.1.8. LOUDNESS SCALING

	Test	Test
Listening	CI	HA
CI processor	Preferred	CI turned off
CI program	……	CI turned off
HA program	HA turned off	Loudness fit
N questions	4	4
Word list nr	……	……
Order	……	……
Completed?	☐	☐

Remarks

4.1.9. SMRT

	Test	Retest
Listening	HA	HA
CI processor	CI turned off	
CI program	CI turned off	
HA program	Loudness fit	
Completed?	☐	☐

Remarks

BREAK

4.1.10. PREFERENCE SCALES

Preference scales (i04 + i05) completed? ☐ Yes ☐ No

4.1.11. FITTING

Particularities from previous fitting to be taken into account? ☐ No ☐ Yes, ……………………………….

……

Fitting form (i02) completed? ☐ Yes ☐ No

Particularities relevant for next fitting? ☐ No ☐ Yes, describe here and note on CRF's next visit

……

Remarks

4.1.12. QUESTIONNAIRES

	Given to subject	To be returned
Date	……./……./……	Visit T2
SSQ-12 (I07)	☐	
HUI-3 (I08)	☐	
ICECAP-A (09)	☐	
Sound quality descriptors (I11)	☐	

Remarks

4.1.13. SPEECH AUDIOMETRY

	Test			Retest
Listening	CI			CI
CI processor	Clinical			Clinical
CI program	……			
HA program	HA turned off			
Level (dB SPL)	75	65	55	65
Completed?	☐	☐	☐	☐

Remarks

4.1.14. MATRIX IN QUIET

	Practice		Test	Retest
Listening	CI		CI	CI
CI processor	Clinical program		Clinical program	Clinical program
CI program	……		……	
HA program	HA turned off			
N sentences	10	10	10	10
Word list nr.	……		……	……
Order	1		2	3
Completed?	☐	☐	☐	☐

Remarks

4.1.15. MATRIX IN NOISE

	Practice		Test	Retest
Listening	CI		CI	CI
CI processor	Clinical program		Clinical program	Clinical program
CI program	……		……	
HA program	HA turned off			
N sentences	10	10	10	10
Word list nr	……		……	……
Order	1		2	3
Completed?	☐	☐	☐	☐

Remarks

4.1.16. DATA MANAGEMENT CHECK-UP

Digital data complete and correctly archived on network drive? ☐ Yes ☐ No

→ If no, describe steps to be taken:

……

……

Source documents correctly archived in filing cabinet? ☐ Yes ☐ No

→ If no, describe steps to be taken:

……

……

Remarks

4.1.17. PROTOCOL DEVIATIONS

Were there any protocol deviations? ☐ Yes ☐ No

Remarks

4.1.18. DEVICE DEFICIENCIES

Were there any device deficiencies? ☐ Yes ☐ No

Remarks

4.1.19. ADVERSE EVENTS

Checked and acted on adverse events? ☐ Yes ☐ No

Any signs of tinnitus? ☐ Yes ☐ No

If yes, on what side(s) ☐ Left ☐ Right

If yes, to what extent (VAS 0-10)? Left: ……….. Right: ………….

Any signs of vertigo? ☐ Yes ☐ No

Any signs of oscillopsia? ☐ Yes ☐ No

Remarks

General remarks

4.2. Visit T2 (week 52)

Date: …………/…………../…………..

Bimodal user: ☐ Yes ☐ No, because…………………………………………………………………

4.2.1. TONE AUDIOMETRY

	CI	HA	Unaided	
Processor/Ear	Clinical	Clinical	L	R
Program	……	CI off	CI off	CI off
Completed?	☐	☐	☐	☐

Remarks

4.2.2. SPEECH AUDIOMETRY

	Test			Retest	Test			Retest	Test			Retest
Listening	CI			CI	HA			HA	CIHA			CIHA
CI processor	Clinical			Clinical	CI turned off				Clinical			Clinical
CI program	……				……				……			
HA program	HA turned off				Clinical fit				Clinical fit			Clinical fit
Level (dB SPL)	75	65	55	65	75	65	55	65	75	65	55	65
Completed?	☐	☐	☐	☐	☐	☐	☐	☐	☐	☐	☐	☐

Remarks

BREAK

4.2.3. QUESTIONNAIRES

	Returned and checked
Date	……./……./…...
SSQ-12 (I07)	☐

HUI-3 (I08)	☐
ICECAP-A (09)	☐
Sound quality descriptors (I11)	☐

Remarks

4.2.4. MATRIX IN QUIET

	Practice		Test	Retest
Listening	CI		CI	CI
CI processor	Clinical program		Clinical program	Clinical program
CI program	……		……	
HA program	HA turned off			
N sentences	10	10	10	10
Word list nr	……	……	……	……
Order	1		2	3
Completed?	☐	☐	☐	☐

Remarks

4.2.5. MATRIX IN NOISE

	Practice		Test	Retest	Test	Retest
Listening	CI / CIHA		CI	CI	CIHA	CIHA
CI processor	Clinical		Clinical	Clinical	Clinical	Clinical
CI program	……		……		……	
HA program	Off / clinical		HA turned off		Clinical	Clinical
N sentences	10	10	10	10	10	10
Word list nr	…	…	……	……	……	……
Order	……		……	……	……	……
Completed?	☐		☐	☐	☐	☐
**Test result** (SRT in dB SNR)	……	……	……	……	……	……
**Mean result** (SRT in dB SNR)			……		……	

Remarks

4.2.6. LISTENING EFFORT

	Practice	Test	Test
Listening	CI / CIHA	CI	CIHA
CI processor	Clinical program	Clinical program	Clinical program
CI program	……	……	……
HA program	Off / clinical program	HA turned off	Clinical program
SRT input (in dB SNR)	…….. …….. …….. …….. …….. ……..	…….. …….. …….. …….. …….. ……..	…….. …….. …….. …….. …….. ……..
Order	……	……	……
Completed?	☐	☐	☐

Remarks

BREAK

4.2.7. PREFERENCE SCALES

Preference scales (i04 + i06) completed? ☐ Yes ☐ No

4.2.8. FITTING

Particularities from previous fitting to be taken into account? ☐ No ☐ Yes, ...

....

Fitting form (i02) completed? ☐ Yes ☐ No

CI research processor given back and stored? ☐ Yes ☐ No

HA loaner given back and stored? ☐ Yes ☐ No

Patient informed about HA reimbursement and acted accordingly? ☐ Yes ☐ No

Remarks

4.2.9. PROTOCOL DEVIATIONS

Were there any protocol deviations? ☐ Yes ☐ No

Remarks

4.2.10. DATA MANAGEMENT CHECK-UP

Digital data complete and correctly archived on network drive? ☐ Yes ☐ No

→ If no, describe steps to be taken:

…….

…….

Source documents correctly archived in filing cabinet? ☐ Yes ☐ No

→ If no, describe steps to be taken:

…….

…….

Remarks

4.2.11. DEVICE DEFICIENCIES

Were there any device deficiencies? ☐ Yes ☐ No

Remarks

4.2.12. ADVERSE EVENTS

Checked and acted on adverse events? ☐ Yes ☐ No

Any signs of tinnitus? ☐ Yes ☐ No

If yes, on what side(s) ☐ Left ☐ Right

If yes, to what extent (VAS 0-10)? Left: ……….. Right: ………….

Any signs of vertigo? ☐ Yes ☐ No

Any signs of oscillopsia? ☐ Yes ☐ No

Remarks

General remarks

End of study

Date of termination of participation in the study:

Date: ………..../……………/……………

Did there occur early termination? ☐ Yes ☐ No

If yes, specify the reason of early termination.

☐ (Serious) adverse event ☐ Retraction of informed consent

☐ Protocol deviation ☐ Did not conform to inclusion criteria

☐ No effect of CI ☐ Discompliance to CI randomization scheme

☐ Discompliance to other study procedures

☐ ………
………
………

INVESTIGATOR STATEMENT

I am confident that the information supplied in this case record form is complete and accurate data. I confirm that the study was conducted in accordance with the protocol and any protocol amendments and that written informed consent was obtained prior to the study.

Principal investigator:

Principal investigator's Signature:

Date of signature (DD-MM-YYYY):

(SERIOUS) ADVERSE EVENTS (SA/SAE)

Serious (see *)	Severity	Relationship to study treatment	Action taken regarding study intervention	Outcome of AE
1 = YES<br>2 = NO	1 = MILD<br>2 = MODERATE<br>3 = SEVERE	1 = DEFINITELY related<br>2 = POSSIBLY related<br>3 = NOT related	1 = NONE<br>2 = DISCONTINUED Permanently<br>3 = DISCONTINUED Temporarily	1 = RESOLVED, no sequel<br>2 = AE still PRESENT – NOT treated<br>3 = AE still PRESENT – Treated<br>4 = RESIDUAL effects present – NOT treated<br>5 = RESIDUAL effects present – Treated<br>6 = UNKNOWN

* Criteria for SAE: Any untoward medical occurence that at any dose (1) results in death, (2) is life-threatening, (3) requires inpatient hospitalisation or prolongation of existing hospitalization, (4) results in persistent or significant disability incapacity or (5) is a congenital anomaly/birth defect.
If serious = YES → complete SAE form!!!

Nr.	Date	Adverse Event	Start date	Stop date	Serious?	Severity	Relationship	Action	Outcome	Initials + Sign
1.										
2.										
3.										
4.										
5.										
6.										
7.										

SUBJECT ID:____________ SEEN BY MONITOR: _____________________ DATE: _____/_____/________

8.										
9.										
10.										
11.										
12.										
13.										
14.										
15.										
16.										
17.										
18.										

PROTOCOL DEVIATIONS

Nr.	Date	Deviation
1.		
2.		
3.		
4.		
5.		
6.		
7.		
8.		
9.		
10.		
11.		

SUBJECT ID:____________ SEEN BY MONITOR: _____________________ DATE: _____/_____/________

PROTOCOL DEVIATIONS

Nr.	Date	Deviation
1.		
2.		
3.		
4.		
5.		
6.		
7.		
8.		
9.		
10.		
11.		

SUBJECT ID:____________ SEEN BY MONITOR: _____________________ DATE: _____/_____/________

CHANGES IN MEDICATION USE

Medication	Start date of use	End date of use	Indication	Subscribed due to (S)AE?

COMPLIANCE

See SOP Compliance for methods and fitting forms for calculations.

CI

In the table below it is shown to what extent subjects can show non-compliance to the CI randomization schedule before consequences take effect.

	F4	F7	F11
Study continuation			
Removed from per protocol population		When 2x >15%	When 2x >15%
Transferred to clinical care	When >40% x 1	When 2x >25% or >40% x 1 or 1x total usage time <360 min/d	When 2x >25% or >40% x 1 or 1x total usage time <360 min/d

The compliance result that is calculated on visit F11, and includes the time window between F7 and F11, counts double since its time window is also double as long as compared to the results calculated on visits F4 and F7.

HA

In the table below it is shown to what extent subjects can show non-compliance before they will be removed from the HA population.

	F7	F14
Study continuation		
No bimodal testing at visit F10	When < 25%	
Removed from HA population		When < 50%

FORMS

Form nr	Contents
i01	Operation Room
i02	Fitting Form
i03	Preference scale for randomization phase
i03	Preference scale for whole study period except randomization phase
i05	Preference scale for bimodal phase
i06	Preference scale for clinical phase
i07	SSQ questionnaire
i08	HUI questionnaire
i09	ICECAP questionnaire
i10	Sound quality questionnaire
i11	CI compliance form
i12	HA compliance form
