# Supplementary material for: Evaluating hearing performance with cochlear implants within the same patient using daily randomization and imaging-based fitting - The ELEPHANT study
Source: Trials. 2020 Jun 23;21:564. doi: 10.1186/s13063-020-04469-x (PMC7310427; doi:10.1186/s13063-020-04469-x)
Supplement: Supplementary file 3 — Additional file 3. Time and events schedule. [file 13063_2020_4469_MOESM3_ESM.pdf]

							FIRST PHASE															SECOND PHASE	THIRD PHASE	
**CI**			candidacy	preop	OR	ENT	Daily randomization											Free Choice		Shared decision		Preferred	Clinical fit	Final fit
**HA (contra)**							first fit		REM													Optimize	Choice	
Timeline		# visit code	-	-	-	-	F1	F2	F3	F4	F5	F6	F7	F8	F9	F10	F11	F12	F13	F14	F15	S1	T1	T2
		# weeks			-4	-3	0	1	2	3	4	5	6	7	8	10	12	16	20	26A	26B	30	34	52
		# months									1		1,5				3			6	6	7	8	12
		relative time cfr. previous visit	var	-1M	-	+9D	+4W	+1W	+1W	+1W	+1W	+1W	+1W	+1W	+1W	+2W	+2W	+4W	+4W	+6W		+4W	+4W	+18W
		window cfr. previous visit	var	±1M	-	±3D	±3W	±5D	±5D	±5D	±5D	±5D	±5D	±5D	±5D	±1W	±1W	±2W	±2W	±2W		±2W	±2W	±4W
Clinical rehabilitation		Audiologist (fitting CI/HA)					**150**[1]	**75**[2]	**90**[3]	**75**[2]			**75**[2]				**60**[2]			**45**[4]		**60**[5]	**60**[6]	**60**[7]
		Speech therapist (rehab)						45	45	45	45	45	45	45	45	45	45							
		Social worker (consult)									60									60				60
Audiometry	unaided	Thresholds + CNC	20	20			**20**													**20**				20
	HA	Aided thresholds	10^{E}						$\mathbf{5^{E}}$											$\mathbf{5^{E}}$			$\mathbf{5^{C}}$	5^{D}
	CI	Aided thresholds							$\mathbf{10^{A}}$				$\mathbf{10^{A}}$							$\mathbf{5^{A*}}$				5^{D}
REM	HA	Aided	15						**15**															
Imaging		CT/MRI	x																					
		CBCT				**x**																		
CI-data		M-T levels					0	0	0				0				0			0				
		Datalogging						0	0	0	**2**	**2**	0	**2**	**2**	**2**	**0**	**2**	**2**	0		**0**	0	0
		Impedances			x		0	0	0	0	**2**	**2**	0	**2**	**2**	**2**	**0**	**2**	**2**	0		**0**	0	0
		Cross-impedances			**x**			**0**							**0**					**0**				
		ECAP			**x**			**20**							**20**					**20**				
Basic tests	CI	CNC					$\mathbf{10^{A}}$	$\mathbf{10^{A}}$	$\mathbf{10^{A}}$	$\mathbf{10^{A}}$	$\mathbf{10^{A}}$	$\mathbf{10^{A}}$	$\mathbf{10^{A}}$	$\mathbf{10^{A}}$	$\mathbf{10^{A}}$	$\mathbf{10^{A}}$	$\mathbf{10^{A}}$	$\mathbf{10^{A}}$	$\mathbf{10^{A}}$		$\mathbf{5^{A*}}$	$\mathbf{5^{B}}$	$\mathbf{10^{C,D}}$	$\mathbf{5^{D}}$
	CI	Matrix quiet					$\mathbf{20^{A}}$	$\mathbf{20^{A}}$	$\mathbf{20^{A}}$	$\mathbf{20^{A}}$	$\mathbf{20^{A}}$	$\mathbf{20^{A}}$	$\mathbf{20^{A}}$	$\mathbf{20^{A}}$	$\mathbf{20^{A}}$	$\mathbf{20^{A}}$	$\mathbf{20^{A}}$	$\mathbf{20^{A}}$	$\mathbf{20^{A}}$		$\mathbf{10^{A*}}$	$\mathbf{10^{B}}$	$\mathbf{20^{C,D}}$	$\mathbf{10^{D}}$
	CI	Matrix noise					$\mathbf{20^{A}}$	$\mathbf{20^{A}}$	$\mathbf{20^{A}}$	$\mathbf{20^{A}}$	$\mathbf{20^{A}}$	$\mathbf{20^{A}}$	$\mathbf{20^{A}}$	$\mathbf{20^{A}}$	$\mathbf{20^{A}}$	$\mathbf{20^{A}}$	$\mathbf{20^{A}}$	$\mathbf{20^{A}}$	$\mathbf{20^{A}}$		$\mathbf{10^{A*}}$	$\mathbf{10^{B}}$	$\mathbf{20^{C,D}}$	$\mathbf{10^{D}}$
	HA	CNC	15^{E}				$\mathbf{5^{E}}$		$\mathbf{5^{E}}$	$\mathbf{5^{E}}$						$\mathbf{5^{E}}$					$\mathbf{5^{E}}$	$\mathbf{5^{B}}$	$\mathbf{5^{C}}$	$\mathbf{5^{D}}$
	CIHA	CNC								$\mathbf{10^{A}}$						$\mathbf{10^{A}}$					$\mathbf{5^{A*}}$	$\mathbf{5^{B}}$	$\mathbf{5^{C}}$	$\mathbf{5^{D}}$
	CIHA	Matrix noise								$\mathbf{20^{A}}$						$\mathbf{20^{A}}$					$\mathbf{10^{A*}}$	$\mathbf{10^{B}}$	$\mathbf{10^{C}}$	$\mathbf{10^{D}}$
Summed up estimated time basic tests			*15*				*55*	*50*	*55*	*85*	*50*	*50*	*50*	*50*	*50*	*85*	*50*	*50*	*50*		*45*	*45*	*70*	*45*
Extended tests	CI + CIHA	Matrix noise spatial														$\mathbf{80^{A}}$					$\mathbf{40^{A*}}$	$\mathbf{40^{B}}$	$\mathbf{40^{C}}$	
	CI + CIHA	Listening effort									$\mathbf{24^{A}}$						$\mathbf{24^{A}}$				$\mathbf{12^{A*}}$	$\mathbf{12^{B}}$	$\mathbf{12^{C}}$	$\mathbf{12^{D}}$
	CI + HA	Loudness scaling									$\mathbf{18^{A}}$						$\mathbf{18^{A}}$				$\mathbf{12^{A*}}$	$\mathbf{12^{B}}$	$\mathbf{12^{C}}$	
	CI	SMRT									$\mathbf{20^{A}}$						$\mathbf{20^{A}}$				$\mathbf{20^{A*}}$		$\mathbf{10^{C}}$	
Questionnaires		SSQ-12		x																A*			C	D
		HUI-3		x																A*			C	D
		ICECAP-A		x																A*			C	D
		Preference scales					A	A	A	A	A	A	A	A	A	A	A	A	A	A*			C	D
		Sound quality								A							A			A*			C	D
Compliance checks		CI								x			x				x							
		HA											x							x				
Trial administration		Adverse events			x	x	x	x	x	x	x	x	x	x	x	x	x	x	x	x	x	x	x	x
		Extended adverse events					x											x				x	x	x
		Data management check-up					x											x				x	x	x
		Device deficiencies			x	x	x	x	x	x	x	x	x	x	x	x	x	x	x	x	x	x	x	x
Invoice travel expences																				x				x
Total estimated time (in minutes) per visit (excluding questionnaires and imaging)			60	20	NA	NA	225	190	220	205	217	99	180	99	119	214	217	54	54	119	165	169	199	207
Reference time standard clinical rehabilitation			*60*	*20*	*NA*	*NA*	*120*	*105*	*120*	*105*	*105*	*45*	*120*	*45*	*45*	*45*	*45*	*0*	*0*	*195*		*0*	*0*	*195*

var = variable moment

extra visit (not combined with clinical visit)

deviant from clinical rehabilitation (e.g. additional test, visit takes more time)

Test conditions

A = measure outcome with standard AND natural FAT (HA = bimodal fit)
A*= measure with preferred FAT (standard OR natural (HA = bimodal fit)
B = measure with HA loudness fit in acute setting (CI=preferred FAT)
C = measure with HA loudness fit after acclimatisation (CI=preferred FAT)
D = measure with CI and HA in clinical fit
E = *candidacy* : measure both hearing aids seperately and in case of a bilateral HA user also bilateral for CNC; *postop* : measure HA contra
0 = time included in fitting CI

Test	testing properties	# administrations per testing property	Estimated time
**Thresholds**	250-8000Hz	1x	5 min per ear/condition
**CNC**	55 dB, 65 dB, 75dB	1x (retest 65 dB)	5 min per ear/condition
**Matrix Quiet**	65dB	2x (test-retest)	10 min per condition
**Matrix Noise**	N65dB, Svariable	2x (test-retest)	10 min per condition
**Matrix noise spatial**	S0NCI, S0NHA	2x (test-retest)	20 min per condition
**Listening effort**	6 SNR	5x per SNR	6 min per condition
**Loudness scaling (Acalos)**	5 noise frequencies	1x	6 min per condition
**SMRT**	Frequency selectivity test	2x (test-retest)	10 min per condition

Fitting legend

[1] Initial CI-fitting in week 1 according to clinical practices, including impedance measurement and determination of M-T levels, subsequently initial fitting of HA contra. In case of ipsilateral residual hearing: try-out EAS live and decide whether or not to continuate study.
[2] CI-fitting according to clinical practices, including impedance measurement, datalogging and determination of M-T levels, followed by fitting of HA contra
[3] CI-fitting according to clinical practices, including impedance measurement, datalogging and determination of M-T levels, followed by extensive fitting of HA contra based on REM
[4] CI evaluation fitting according to clinical practices, including impedance measurement, datalogging and determination of M-T levels, followed by evaluation of HA fitting
[5] HA optimization fitting, CI fitting limited to check of impedances and datalogging
[6] Programming of preferred HA fitting at the end of testing, programming of preferred CI fitting with Soundwave instead of BEPS+
[7] CI evaluation fitting according to clinical practices, including impedance measurement and datalogging, followed by evaluation of HA fitting
